# Dietary analysis reveals differences in the prey use of two sympatric bat species

**DOI:** 10.1002/ece3.8472

**Published:** 2021-12-16

**Authors:** Olga Heim, Anna I. E. Puisto, Ilari Sääksjärvi, Dai Fukui, Eero J. Vesterinen

**Affiliations:** ^1^ Faculty of Life and Medical Sciences Doshisha University Kyotanabe Japan; ^2^ Centre for Population Health Research University of Turku Turku Finland; ^3^ Biodiversity Unit University of Turku Turku Finland; ^4^ The University of Tokyo Hokkaido Forest The University of Tokyo Furano Japan; ^5^ Department of Biology University of Turku Turku Finland

**Keywords:** Chiroptera, DNA metabarcoding, Japan, *Murina ussuriensis*, *Myotis ikonnikovi*, University of Tokyo Hokkaido Forest

## Abstract

One mechanism for morphologically similar and sympatric species to avoid competition and facilitate coexistence is to feed on different prey items within different microhabitats. In the current study, we investigated and compared the diet of the two most common and similar‐sized bat species in Japan—*Murina ussuriensis* (Ognev, 1913) and *Myotis ikonnikovi* (Ognev, 1912)—to gain more knowledge about the degree of overlap in their diet and their foraging behavior. We found that both bat species consumed prey from the orders of Lepidoptera and Diptera most frequently, while the proportion of Dipterans was higher in the diet of *M*. *ikonnikovi*. Furthermore, we found a higher prey diversity in the diet of *M*. *ikonnikovi* compared to that of *M*. *ussuriensis* that might indicate that the former is a more generalist predator than the latter. In contrast, the diet of *M*. *ussuriensis* contained many Lepidopteran families. The higher probability of prey items likely captured via gleaning to occur in the diet of *M*. *ussuriensis* in contrast to *M*. *ikonnikovi* indicates that *M*. *ussuriensis* might switch between aerial‐hawking and gleaning modes of foraging behavior. We encourage further studies across various types of habitats and seasons to investigate the flexibility of the diet composition and foraging behavior of these two bat species.

## Graphical abstract

The two common, but understudied Japanese bat species *Murina ussuriensis* and *Myotis ikonnikovi* consumed prey from the orders of Lepidoptera and Diptera most frequently. Furthermore, we found a higher prey diversity in the diet of *M*. *ikonnikovi* compared to that of *M*. *ussuriensis* that might indicate that the former is a more generalist predator than the latter. Our results also indicate that *M*. *ussuriensis* might switch between aerial‐hawking and gleaning modes of foraging behavior, while *M*. *ikonnikovi* seems to use predominantly aerial hawking.

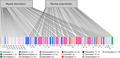

## 1 INTRODUCTION

One of the prominent research fields in ecology is the study of species coexistence mechanisms that explain how resources are allocated and coexistence of, for example, morphologically similar and sympatric species is facilitated (Chesson, 2000; Gómez‐Llano et al., 2021). The group of bats is an interesting target group in that respect, as they are a very specious, diverse group and many species are in part or obligatory insectivorous. Several interesting studies have been published that investigated how morphologically similar, sympatric bat species coexist (Arrizabalaga‐Escudero et al., 2018; Geipel et al., 2021; Novella‐Fernandez et al., 2020; Roeleke et al., 2018).

Many studies were conducted in Europe or the Americas, whereas studies on bats in Asia are rare. To close this knowledge gap, this study aims at investigating the diet of Japanese bat species. *Murina ussuriensis* (Ognev, 1913) and *Myotis ikonnikovi* (Ognev, 1912) are two very common, sympatric and similar‐sized bat species in Japan that are thought to strongly depend on the availability of forests. They also occur in South and North Korea, Northern China, and Far East Russia, although the range of *M*. *ikonnikovi* extends further west across Mongolia, the southern center of Russia, and Kazakhstan (Fukui et al., 2019; Stubbe et al., 2008). Both species are listed as of “least concern” in the IUCN red list (Fukui et al., 2019; Zhigalin et al., 2020), although their population trends are decreasing (*M*. *ikonnikovi*) or unknown (*M*. *ussuriensis*) and further research on their habitat use and ecology is encouraged (Fukui et al., 2019; Stubbe et al., 2008). Regarding their habitat use, we know that these two species occur predominantly in deciduous or mixed forests, sometimes in proximity to rivers (Fukui et al., 2019; Kim et al., 2014; Sato et al., 2011; Stubbe et al., 2008; Sugai et al., 2011), with *M*. *ussuriensis* being presumably more dependent on forests as foraging habitats than *M*. *ikonnikovi* (Fukui et al., 2019; Stubbe et al., 2008). They usually roost in tree cavities, in crevices under tree bark, in manmade structures or, in the case of *M*. *ussuriensis*, also within dead leaves (Fukui et al., 2012; Ohdachi et al., 2015) during summer. Both species move only locally, based on results of population genetics (Flanders et al., 2016) and radio‐tracking data (Jo, 2015). For example, nursing *M*. *ussuriensis* females were found to frequently change roosts that were located within a maximum distance of 335 m from each other (Fukui et al., 2012). *Myotis ikonnikovi* was also reported to move no further than about 360 m on a daily basis (Jo, 2015).

These two species appear to be similar in their habitat use, but there are indications that they might use different microhabitats. Both bat species are similar in size (forearm length: *M*. *ussuriensis*: ~30 mm, *M*. *ikonnikovi*: ~33 mm, Ohdachi et al., 2015), but the wing area of *M*. *ussuriensis* has a lower aspect ratio and more rounded wingtips than *M*. *ikonnikovi* (*M*. *ussuriensis*: mean aspect ratio = 5.6, mean wingtip shape index = 4.5, *M*. *ikonnikovi*: mean aspect ratio = 5.9, mean wingtip shape index = 1.8, values were obtained from 7 and 13 individuals, respectively, please refer to Fukui et al. (2011) for further details, Ohdachi et al., 2015), which would enable it to fly more slowly and be more maneuverable through cluttered habitats and to glean prey from the ground or leaves in hovering flight (Norberg and Rayner, 1987). The reported foraging behavior of *M*. *ikonnikovi* that forage on emerging aquatic insects above rivers by aerial hawking (Fukui et al., 2006) and of *M*. *ussuriensis* that forage in slow flight and glean prey from the ground or other surfaces in hovering flight (Jo, 2015) appears to be congruent with the differences in wing morphology.

Slight differences in echolocation call characteristics also exist: Although both species use FM calls (frequency modulated), the bandwidth of calls from *M*. *ussuriensis* is larger while the peak frequency is higher and the call duration is smaller compared to the calls of *M*. *ikonnikovi* (Fukui et al., 2004, 2015). The intensity of calls from *M*. *ussuriensis* is also lower compared to calls from *M*. *ikonnikovi* (Fukui et al., 2004). In summary, the low‐intensity, broadband, and short calls of *M*. *ussuriensis* are well adapted for detecting arthropods in highly cluttered habitats such as forest understory (Neuweiler, 1989; Schnitzler et al., 2003). The calls of *M*. *ikonnikovi*, in contrast, are less well adapted to such habitats and might be better suited for foraging in narrow or edge space (Schnitzler et al., 2003). Therefore, we hypothesize that minor differences in wing morphology and echolocation call characteristics might lead to differential microhabitat use (Siemers & Schnitzler, 2004), thereby enabling these two bat species to evade interspecific competition for resources. However, accurate dietary information for these bat species is currently lacking and is needed to confirm the above assumptions on microhabitat use.

Only one study so far, to our knowledge, has investigated the diet composition of these two bat species using a morphological approach (Sato & Katsuta, 2018). According to that study, *M*. *ussuriensis* consumed mainly prey from the order of Coleoptera and to a lesser degree from orders of Lepidoptera and Orthoptera and a few other orders. In contrast, *M*. *ikonnikovi* consumed mainly prey from the orders of Coleoptera and Lepidoptera and to a lesser degree from the order of Hemiptera including less frequently consumed prey from other orders. To complement the picture on diet composition of the two bat species, this study aims to investigate, describe, and compare the diet composition of *M*. *ussuriensis* and *M*. *ikonnikovi* using DNA metabarcoding, which works well for bats (Pompanon et al., 2012). We expect to find differences in the diet composition between the two bat species as a consequence of the above‐mentioned differences in wing morphology and echolocation call characteristics. In particular, because *M*. *ussuriensis* is thought to also rely on gleaning as a foraging mode, we expect to find a higher proportion of prey items that might have been captured in this mode in the diet of *M*. *ussuriensis* than in the diet of *M*. *ikonnikovi*. Furthermore, we expect to find a higher proportion of insects that are associated with aquatic habitats in the diet of *M*. *ikonnikovi* compared to *M*. *ussuriensis* as the former were observed to forage at such habitats. The findings of this study are expected to complement and expand our knowledge of the diet composition of these two bat species along with existing morphological analyses.

## 2 MATERIAL AND METHODS

### 2.1 Study area

The study area is the University of Tokyo Hokkaido Forest (UTHF: 43˚10’–21’N, 142˚23’–41’E, 190–1,459 m a.s.l.) located in Furano, the center of Hokkaido island, that is situated southwest of the Tokachidake mountain range and the upper area of the Sorachi river within the Ishikari river basin. The forest's area of 22,717 ha is characterized by natural hemiboreal forest with coniferous and broad‐leaved tree species. Dominant tree species are Sakhalin fir (*Abies sachalinensis*), Yezo spruce (*Picea jezoensis*), Japanese linden (*Tilia japonica*), and painted maple (*Acer pictum* var. *mono*). The mean annual temperature and precipitation was 6.5ºC and 1279 mm, respectively, from 2001 to 2018 (http://www.uf.a.u‐tokyo.ac.jp/research_division/data/kishou/index.html). Daily average temperatures during this study's sampling periods ranged from 4.6°C to 20.5°C in May, the coolest month of the study period, and from 14.0°C to 25.8°C in August, the warmest month of the year (http://www.uf.a.u‐tokyo.ac.jp/research_division/data/kishou/index.html). The monthly precipitation ranged between 62.0 mm and 407.5 mm from May to September (http://www.uf.a.u‐tokyo.ac.jp/research_division/data/kishou/index.html).

### 2.2 Bat trapping and fecal sampling

**FIGURE 1:**
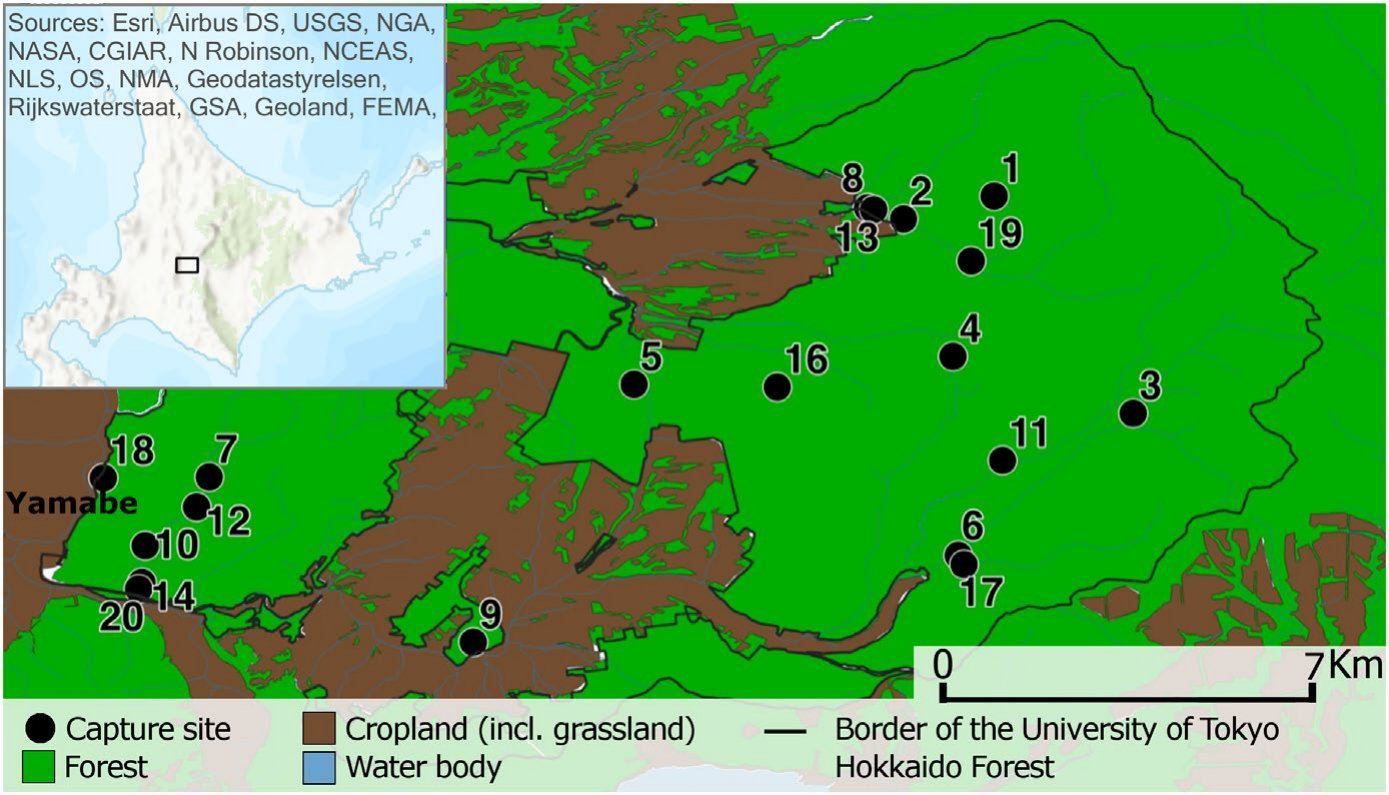
The main map shows the locations of trapping sites within the University of Tokyo Hokkaido Forest, while the inset map shows the location of the study area within Hokkaido. Main map data source: National Land Numerical Information (land utilization tertiary polygon data) provided from the Japanese Ministry of Land, Infrastructure, Transport and Tourism (https://nlftp.mlit.go.jp/ksj/index.html; accessed: 2021/01/21) was edited to create this map

Trapping sites (*n*= 19) were located within mixed forests with conifer and broad‐leaved trees southeast of the UTHF (Figure 1). From these, nine sites lay within forests located close (<1 km distance) to arable fields (5 sites: 2, 6, 8, 13, 17), a river (3 sites: 9, 14, 20) and an urban area (one site: 18). Trapping was conducted at each site once, except at site 1 that was revisited once in July 2017. Bats were caught with permission from the Ministry of Environment (Nos. 21‐27‐0077, 21‐28‐0087, and 21‐29‐0131) and Hokkaido Prefecture (Nos. 26–35 and 57), Japan, following the “Regulations and manual of animal experiments in the University of Tokyo” and the “Guidelines of the Mammal Society of Japan for the use of wild mammals in research” (https://www.mammalogy.jp/guideline.html). We used harp traps and mist nets with acoustic lures (Sussex AutoBat; Hill & Greenaway, 2005) to capture the bats during the night. We determined the sex, reproductive status, and age (adult or juvenile) for all individuals, measured their weight and forearm length, and collected fecal samples either directly from the individual while handling or from clean cotton bags used to hold bats. Bats were released immediately after fecal samples were obtained during handling or kept up to 30 min. Fecal pellets were subsequently stored in 70% ethanol at −20°C until further laboratory analyses.

### 2.3 Laboratory work

We let the ethanol that was covering the fecal pellets evaporate as much as possible before extracting DNA using the QIAamp PowerFecal DNA Kit (Qiagen/MoBio cat. nr 12830‐50, Qiagen, Hilden, Germany). We followed the extraction manual (MoBio “Protocol: Detailed”; version 12192013) with modifications as in Heim et al. (2019). The DNA was eluted into 100 μl of C6 buffer as recommended in the protocol and stored at –20°C until subsequent analysis.

We used a single bat‐specific primer pair targeting the DNA barcode region in the mitochondrial *cytochrome oxidase subunit I* (SFF‐145f: 5’‐GTHACHGCYCAYGCHTTYGTAATAAT‐3’ and SFF‐351r: 5’‐CTCCWGCRTGDGCWAGRTTTCC‐3’; Walker et al., 2016) to confirm the bat species in the fecal samples. Successful PCR products were purified using A’SAP clean kit (product nr 80350, ArcticZymes, Trømssa, Norway) and sequenced by Sanger sequencing at Macrogen Europe (Macrogen Inc., Seoul, South Korea). Sequences were trimmed for poor quality regions, and primers were removed as described in Sorvari et al. (2012) using Geneious R6 (Kearse et al., 2012). Trimmed sequences were identified using BOLD systems (Ratnasingham & Hebert, 2007) and blasted against the GenBank database (Altschul et al., 1990).

We used two primer pairs to amplify the potential arthropod prey. Firstly, we applied widely utilized primers targeting the so‐called DNA barcode region in the COI gene (ZBJ‐ArtF1c: 5’‐AGATATTGGAACWTTATATTTTATTTTTGG‐3’ and ZBJ‐ArtR2c: 5’‐WACTAATCAATTWCCAAATCCTCC‐3’; Zeale et al., 2011; this dataset was abbreviated later as COI). Secondly, we amplified the 16S gene region, which is one of the most common markers for animal DNA barcoding next to COI; this region is more conserved than COI, so 16S primers usually amplify a larger set of taxa (Ins16S‐1F: 5’‐TRRGACGAGAAGACCCTATA‐3’ and Ins16S‐1Rshort: 5’‐ACGCTGTTATCCCTAARGTA‐3’; Clarke et al., 2014; the fourth last nucleotide in the reverse primer changed from G to R as used in Kaunisto et al., 2017; this data set was abbreviated as 16S). PCR blanks (containing sterile water instead of a sample) were included for each batch (see Appendix S5: A1 for further information).

The PCR and library construction followed Kaunisto et al. (2020). In summary, the first‐step PCR reactions included linker‐tagged, locus‐specific primers targeting either the COI or 16S gene, and the second‐step PCR followed directly, including Illumina‐specific adapters with a unique dual‐index combination for every single reaction and the same linker‐tags as the first‐step PCRs. The first‐step PCRs were set up as technical duplicates, and both duplicates had unique index combinations in the second PCR stage. The samples were then pooled by equal volume and purified using SPRI beads as in Vesterinen et al. (2016). Sequencing was performed on the Illumina MiSeq platform (Illumina Inc., San Diego, California, USA) by the Turku Centre for Biotechnology, Turku, Finland, using v2 chemistry with 300 cycles and 2 × 150 bp paired‐end read length.

### 2.4 Bioinformatics and prey list construction

The reads were uploaded directly from the sequencing facility to the CSC Taito server (IT Center for Science, www.csc.fi) for trimming and further analysis. Trimming and quality control of the sequences were conducted according to Kaunisto et al. (2017). Consequently, paired‐end reads were merged and trimmed for quality using program USEARCH (Edgar, 2010). Primers were removed using the Python program Cutadapt (Martin, 2011). The reads were then collapsed into unique sequences (discarding haplotypes with abundance <10). Chimeras were removed and reads were clustered into ZOTUs (Zero‐radius Operational Taxonomic Units) using USEARCH ‘unoise3’ algorithm (Edgar, 2016) and then mapped back to the original trimmed reads to establish the total number of reads in each sample using VSEARCH ‘search_exact’ algorithm (Rognes et al., 2016).

ZOTUs were assigned to species using slightly different methods depending on the dataset. For COI, ZOTUs were identified using the Python software Bold‐retriever (Vesterinen et al., 2016) or by local BLAST (Altschul et al., 1990) against all Arthropoda sequences downloaded from BOLD (Ratnasingham & Hebert, 2007). For 16S, we used BLAST (without low‐complexity filtering or masking) against all sequences in the GenBank nt database. Further taxa names (BIN, species name or higher taxonomy) were retrieved for both COI and 16S using a custom script written for this purpose. See details on ZOTU clustering in Appendix S5: A2.

We used different criteria for including prey species in the data depending on the dataset. For COI, (1) at least 10 reads of the final assigned prey species were required to be present in the final data, and (2) sequence similarity with the reference sequence had to be at least 98% for the OTU to be given species assignation, and lower hits were assigned to higher taxa (96%–98% to family level and 90%–96% to order level). For 16S, (1) at least 10 reads of the final assigned prey species were required to be present in the final data, and (2) sequence similarity with the reference sequence had to be at least 90%, (3) alignment length at least 100 bp to the target region, and (4) E‐value lower than 0.0000001. We assigned the ZOTUs to taxa as accurately as possible and confirmed that all the prey species were recorded either from Hokkaido or from Japan (including Hokkaido) based on the information given in the literature (please find the complete list of literature in the Appendix S5: A3). We furthermore checked whether the next higher taxonomic group occurs in Hokkaido or Japan if a given insect species was found to occur elsewhere. We consequently obtained a prey list containing species, genera, subfamilies, and families that occur within Hokkaido or Japan (Appendix S1).

### 2.5 Data analysis

#### 2.5.1 Data preparation

We removed samples with data deemed unreliable (<1000 reads) prior to further analyses. Furthermore, samples with overall more than 1000 reads but no reads for prey species were excluded from the diet analysis.

We combined the prey item lists obtained from COI and 16S markers to analyze and compare the diet composition within and between bat species. We removed one of two occurrences to avoid counting prey item presence twice in cases where insect species were identified by both markers within the same sample.

#### 2.5.2 Calculation of species richness estimators and accumulation curves

The read count for each insect prey in all samples was transformed into incidence data. To estimate to what degree the prey richness in the bat species’ diet was covered by our sampling effort, these data were used to compute species‐level accumulation curves and the ICE estimator (Lee & Chao, 1994) based on 100 permutations using EstimateS (Version 9, Colwell, 2013). The incidence counts of species within a genus, family, and order, respectively, were summed up for the computation of genus‐, family‐, and order‐level accumulation curves (e.g., 5 for genus A, if it contained 5 species or 10 for family X, if there were 10 species within the respective number of genera). The ACE estimator was used in this case (Chao & Lee, 1992).

#### 2.5.3 Calculation of indices

We calculated the percentage of occurrence (POO; Deagle et al., 2019) for each prey species by summing up the respective presence counts over all samples of a bat species and dividing this count by the total sum of prey occurrences (Appendix S2) to compare the diet composition between bat species. Therefore, the sum of all POOs within a bat species equals 1.

Based on the POO data, we calculated the effective number of species (Hill, 1973) across the *q*‐values of 0, 1, and 2 (function hill_div, package hilldiv, Alberdi & Gilbert, 2019). We investigate the influence of rare prey items on the diversity of the total diet by changing the *q*‐value. We also computed more classical diversity measures like the Simpson and Shannon diversity indices for a convenient comparability among different studies. The effective number of species was calculated as *^q^D* = ${\left(\sum_{i=1}^{S}p_{i}^{q}\right)}^{1/\left(1‐q\right)}$ with *p_i_* as the proportion of prey item *i* in the diet, *q* as a value for the order of diversity and *S* as the number of species (insect species in our case). Simpson index was calculated as 1–*D* with *D* = Σ*p_i_* ^2^, where *p_i_* is the proportion of prey item *i* in the diet. The Shannon index was calculated as *H’* = –Σ (*p_i_*) × log_e_ *p_i_*.

We also investigated and compared the numbers of prey items across single samples between bat species by using the unpaired Wilcoxon rank‐sum test (stats, R Core Team, 2020), in addition to the overall diversity of the bat species’ diet.

Furthermore, we calculated Levin's niche breadth index (package spaa_0.2.2, Zhang, 2016) *B* = 1/Σ*p_i_* ^2^ to investigate the degree of diet specialization for each bat species’ diet. The values of this index range from 1—the minimal breadth—to a maximum of the total number of—in our case—consumed insect species. We normalized the respective values to a range between 0, which indicates a highly specialized diet, and 1, in which case every resource available or sampled was consumed, to simplify the interpretation of this index.

We then investigated the degree of dietary overlap between the bat species’ diets using the Pianka's niche overlap index (package spaa_0.2.2, Zhang, 2016) *O_jk_* = Σ*p_ij_ p_ik_*/√Σ*p_ij_* ^2^ Σ*p_ik_* ^2^, where *p_ij_* is the proportion of prey item *i* in the diet of species *j* and *k* in the case of *p_ik_*. We created a Venn Diagram on the species, genus, family, and order level (venn.diagram from package VennDiagram_1.6.20, Chen, 2018) to further visualize the dietary overlap of more important prey items (consumed more than once) between the two bat species. Additionally, we used a Plotweb graph (function plotweb, package bipartite_2.15, Dorman et al., 2008, 2009) to visualize how families of more important prey items were partitioned between the diets of both bat species.

All of the above calculations and statistical tests were conducted in R (R Core Team, 2020) unless otherwise stated.

#### 2.5.4 Predominant mode of foraging

We predicted that the diet of *M*. *ussuriensis* should contain a higher proportion of prey caught via gleaning than the diet of *M*. *ikonnikovi* based on wing morphology and echolocation call characteristics. To test this prediction, we followed the method used by Novella‐Fernandez et al. (2020) and classified the prey items into categories of *volant* (e.g., Chironomidae) versus *non*‐*volant* (e.g., Salticidae) and subsequently classified the *volant* prey items based on their diel activity patterns into categories of *nocturnal* (e.g., Noctuidae) or *diurnal* (e.g., Muscidae). The classification was carried out on the family or a finer taxonomic level and was based on published information (please find the list of citations in Appendix S5: A4 and a table of the classified prey items in the Appendix S3). Following this classification, those prey items that are volant and nocturnal have a high probability of being caught via aerial hawking, while volant but inactive or nonvolant prey items have a high probability of being caught via gleaning (from ground or leaf surfaces). However, nocturnal and volant insects that are resting can be caught via gleaning, too, while, for example, ballooning spiders can also be caught via aerial hawking. Therefore, this classification is meant to reflect the likelihood of each prey item being caught via gleaning or aerial hawking, and this analysis is meant to serve as an estimate of potential differences in foraging styles between the two bat species.

We counted the occurrences of prey items belonging to each category for each sample from both bat species after the final classification of prey items into the categories of the likely capture mode *aerial hawking* or *gleaning* (or *undecided* if a clear classification was not possible). We then modeled the number of prey items that are likely to be caught via gleaning relative to the number of prey items likely caught via aerial hawking as a function of the bat species in a generalized linear mixed effect model (package lme4_1.1‐26, Bates et al., 2015) assuming a binomial error distribution and added a sample‐ID as a random effect term. Hereafter, prey items classified as *undecided* were left out of the analysis. The fit of this model was examined graphically (functions from DHARMa_0.3.3.0, Hartig, 2020), and the model's overall significance was tested against the respective null model using parametric bootstrapping (pbkrtest_0.5‐0.1, Halekoh & Højsgaard, 2014). The statistical comparison between the factor levels was done based on estimated marginal means (emmeans_1.5.4, Lenth, 2019).

In addition to this analysis, we have checked whether prey items from the order of Lepidoptera were most likely consumed as caterpillars or as adults. For this, we focused only on those prey items that were identified to species level and that were consumed more than once by either bat species. Based on the information of the prey species, we have obtained phenological data and matched the months when mainly adults are present at our study site (Hokkaido) with the months when the respective prey species was found in the diet of the respective bat species (Appendix S5: A5).

#### 2.5.5 Pest status check of consumed Lepidoptera and Coleoptera

We mainly searched the Crop Protection Compendium (CABI, 2021) database and published literature (Appendix S4) to estimate whether both bat species also consumed insects that are considered to be agricultural pests. We focused this analysis on prey items that were identified to species level and belonged to the orders of Coleoptera and Lepidoptera, because these two orders are known to contain pest species and both bat species consumed prey items from these orders relatively frequently.

## 3 RESULTS

### 3.1 Sequencing

The Illumina sequencing for the COI dataset yielded 797,129 and 758,312 reads for *M*. *ussuriensis* and *M*. *ikonnikovi*, respectively. For the 16S dataset, we retrieved 1,225,068 and 638,739 reads for *M*. *ussuriensis* and *M*. *ikonnikovi*. The technical PCR replicates were found to be nearly identical, and the number of reads per each replicate for each sample correlated very well (COI: *R* ^2^ = 0.849, 16S: *R* ^2^ = 0.995; Appendix S5: A6). The negative extraction controls and PCR blanks yielded only a few (<0.001% of total reads) for each dataset. The reads originating from bats in the 16S dataset were used to confirm the bat species identity.

The final prey COI data consisted of 786,887 reads (average per sample 16,059 ± 10,821) for *M*. *ussuriensis* and 758,312 reads (average per sample 16,851 ± 9,215) for *M*. *ikonnikovi*. The16S dataset consisted of 1,202,289 reads for *M*. *ussuriensis* (average per sample 22,265 ± 11,070 reads) and 637,162 reads (average per sample 15,929 ± 10,230 reads) for *M*. *ikonnikovi*. After removing the predator‐related sequences, 917,272 prey reads for *M*. *ussuriensis* and 634,153 prey reads for *M*. *ikonnikovi* remained.

### 3.2 Sampling effort

We collected samples from a total of 55 individuals of *M*. *ussuriensis* (28 females, 28 males) and 45 individuals of *M*. *ikonnikovi* (21 females, 21 males) from 2015 to 2017. We had a final dataset of 53 samples for *M*. *ussuriensis* and 45 samples for *M*. *ikonnikovi* (Table 1) after the removal of two samples—one from an adult *M*. *ussuriensis* male that was contaminated by another bat species (DNA from two bat species was found in this sample) and a second one from another adult *M*. *ussuriensis* male that had too few prey‐specific reads in both 16S and COI datasets. Most of the samples were collected in July, August, and September, which represents the summer season in Japan.

**TABLE 1:** Overview shows the number of analyzed samples and their distribution across bat species, sexes (f = female, m = male), age classes (ad = adult, j = juvenile, NA = not available), sampling years, and the capture sites (Figure 1)

Sex	Age	Year	*Murina ussuriensis*		*Myotis ikonnikovi*	
			*n*	Sites	*n*	Sites
f	ad	2015	11	3,4,5,7	10	1,2,3,4
		2016	2	11,13	2	10
		2017	12	1,14,17,19,20	6	1,14,16,17
	j	2015	2	6	1	5
		2016	0	NA	2	10,13
		2017	1	20	0	NA
m	ad	2015	13	1,3,4,5,6,8	6	1,2,3,8
		2016	8	9,10,11,12	1	11
		2017	4	1,16,18	1	1
	j	2015	0	NA	11	5,6,7,8
		2016	0	NA	1	13
		2017	0	NA	1	20
	NA	2016	0	NA	3	10

The sampling effort in the present study covered the order‐level prey richness to 95% and 79% and the family‐level prey richness to 71% and 72% for *M*. *ussuriensis* and *M*. *ikonnikovi*, respectively (Appendix S5: A7). However, the prey richness on the genus and species level was represented to a lesser degree for *M*. *ussuriensis* (COI: 30% and 16S: 22%) than for *M*. *ikonnikovi* (COI: 57% and 16S: 47%) within the present set of samples.

### 3.3 Diet diversity and composition

**FIGURE 2:**
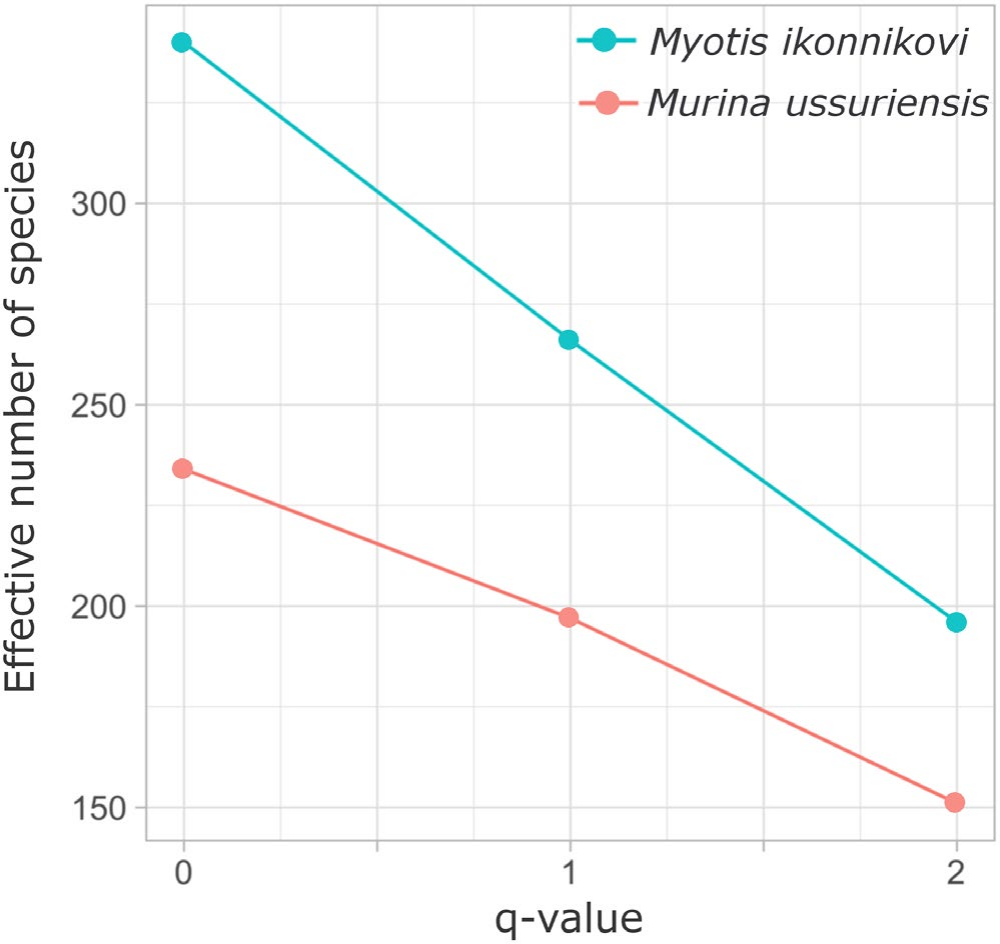
Effective number of species for the diet of *Murina ussuriensis* and *Myotis ikonnikovi* across *q*‐values. The effective number of species was calculated based on the percentage of occurrence values (POO) of prey items in the whole diet of the investigated bat species. At a *q*‐value of 0, each prey item that occurred in the respective bat specie's diet is counted. At a *q*‐value of 1, the effective number of species reflects the number of equally frequent prey items in the respective diet. With a *q*‐value of 2, a higher emphasis is placed on those prey items that were consumed more often

**FIGURE 3:**
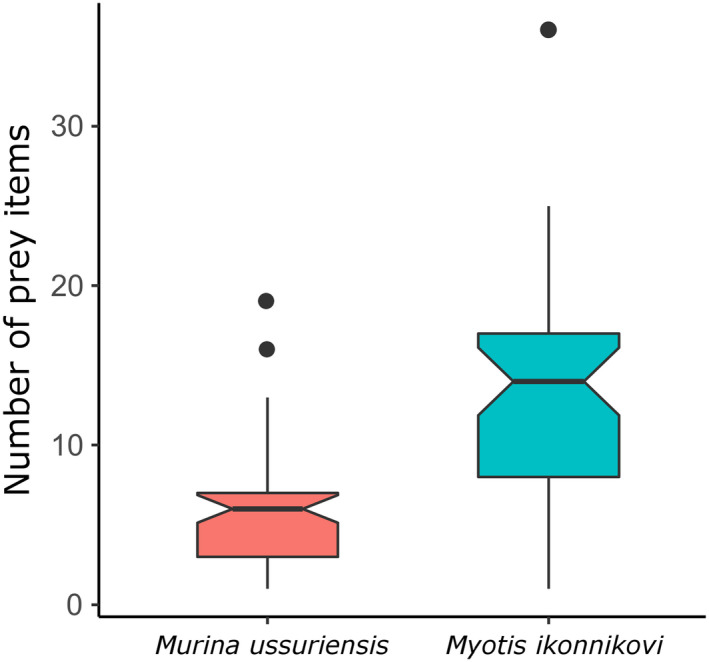
Number of prey items across single samples is shown for both bat species

**FIGURE 4:**
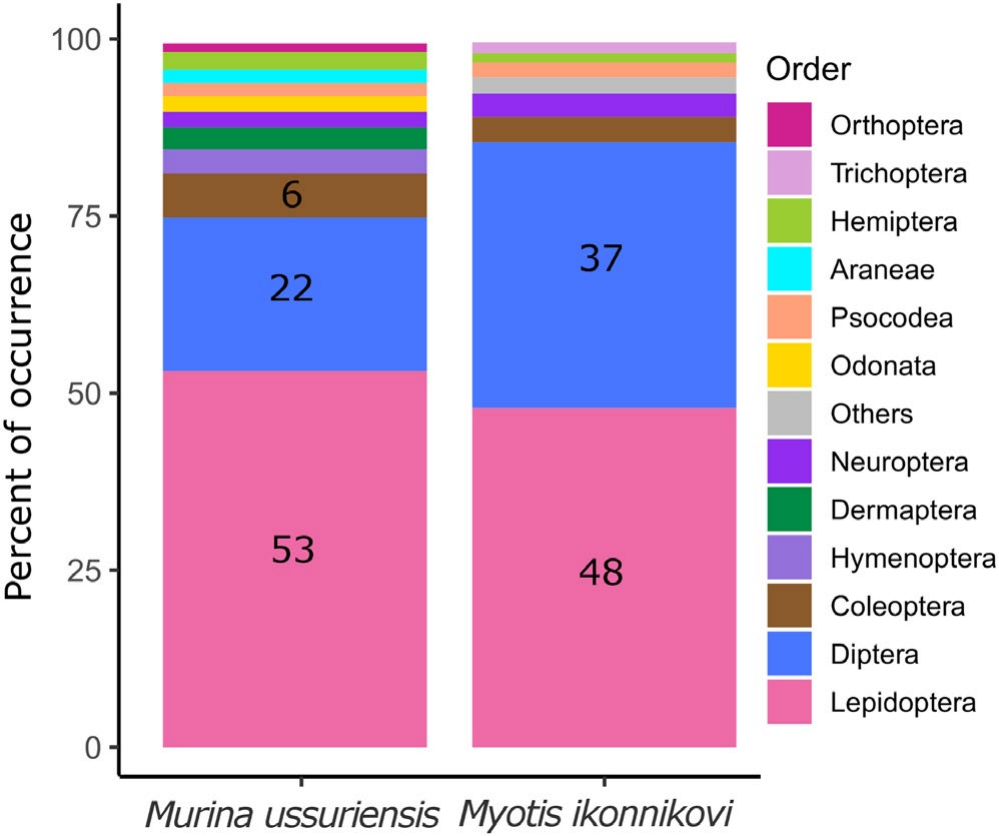
Percentage of occurrence values for prey items on order level in the core diet (prey consumed more than once) of *Myotis ikonnikovi* (*n* = 45) and *Murina ussuriensis* (*n* = 53). Orders that were represented in the diet to <0.5% were plotted as “Others” (*M*. *ikonnikovi*: Ephemeroptera, Mecoptera, Thysanoptera)

*Myotis ikonnikovi* consumed a total of 340 prey items across a range of at least 16 insect orders, while 234 prey items across 13 orders were found in the diet of *M*. *ussuriensis* (Figure 2, *q*‐value = 0). Shannon (*H*) and Simpson Index (*D*) values were accordingly higher for the diet of *M*. *ikonnikovi* compared to that of *M*. *ussuriensis* (*H*_Mik_ = 5.6, *H*_Mus_ = 5.3; *D*_Mik_ = 0.995, *D*_Mus_ = 0.993). The normalized Levin's niche breadth indicated furthermore that, although both bat species had a specialized diet to some degree, *M*. *ikonnikovi* was a more generalist predator compared to *M*. *ussuriensis* (*B*_Mik_ = 0.397, *B*_Mus_ = 0.304). This appears to also be true for the diet composition of individual bats, because we found a significantly higher median number of 14 prey items per sample for *M*. *ikonnikovi* compared to the median of six prey items per sample for *M*. *ussuriensis* (Wilcoxon unpaired rank‐sum test: *W* = 395, *p* < .001, Figure 3). We found that, despite the large diversity of prey items, both bat species consumed those prey items most frequently that belonged to the orders of Lepidoptera (*M*. *ussuriensis*: 53%, *M*. *ikonnikovi*: 48%) and Diptera with *M*. *ikonnikovi* consuming prey from the order Diptera more often (37%) than *M*. *ussuriensis* (22%, Figure 4).

We found that the effective number of species (*q* > 0) for each bat species’ diet decreased, as did the difference in diversity between the bat species’ diets when considering prey items that were consumed more than once (termed “core diet” in the following). At a *q*‐value of 2, the effective number of species for the diet of *M*. *ikonnikovi* remained with a value of 196 higher than the effective number of species of 151 for the diet of *M*. *ussuriensis*. The core diet of both bat species was also composed of prey across an equal number of 12 orders for *M*. *ussuriensis* and *M*. *ikonnikovi*, respectively (Figure 4). However, the composition and the proportions of the different orders within the core diets differed between both species. For example, the orders with less frequently occurring prey across all samples (all except Diptera, Lepidoptera and Coleoptera) took up a larger portion of the core diet of *M*. *ussuriensis* than of the diet of *M*. *ikonnikovi* (Figure 4).

### 3.4 Overlap of diets between bat species

**FIGURE 5:**
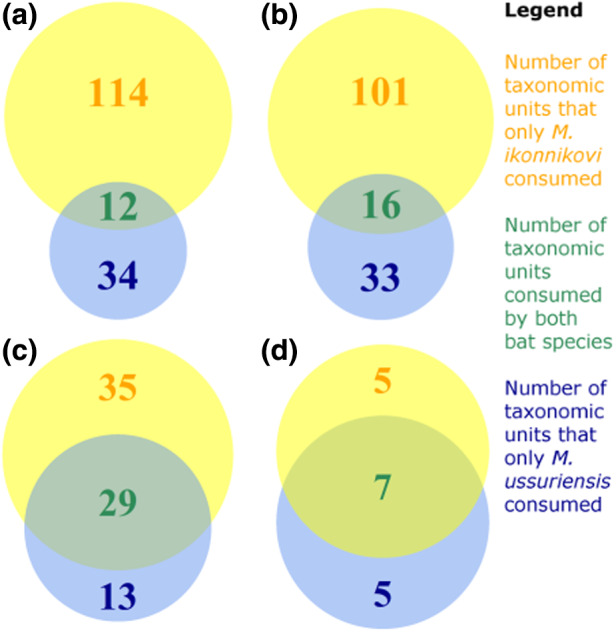
Venn diagrams show the overlap of (a) prey species, (b) genera, (c) families, and (d) orders between the “core” diets of *Murina ussuriensis* and *Myotis ikonnikovi*. For this graph, only those taxonomic units were considered that were consumed by each bat species more than once

A Pianka's index value of 0.31 (with a value of 1 for a complete overlap of diet composition) indicates that the two bat species did not overlap strongly in their total diet composition on the level of insect species. We found a slightly higher overlap based on Pianka's index value of 0.35 (Figure 5a) when we considered the core diet.

On a higher taxonomic level, we found that the two bat species shared more important prey items that belonged to 16 genera, 29 families, and 7 orders (Figure 5).

**FIGURE 6:**
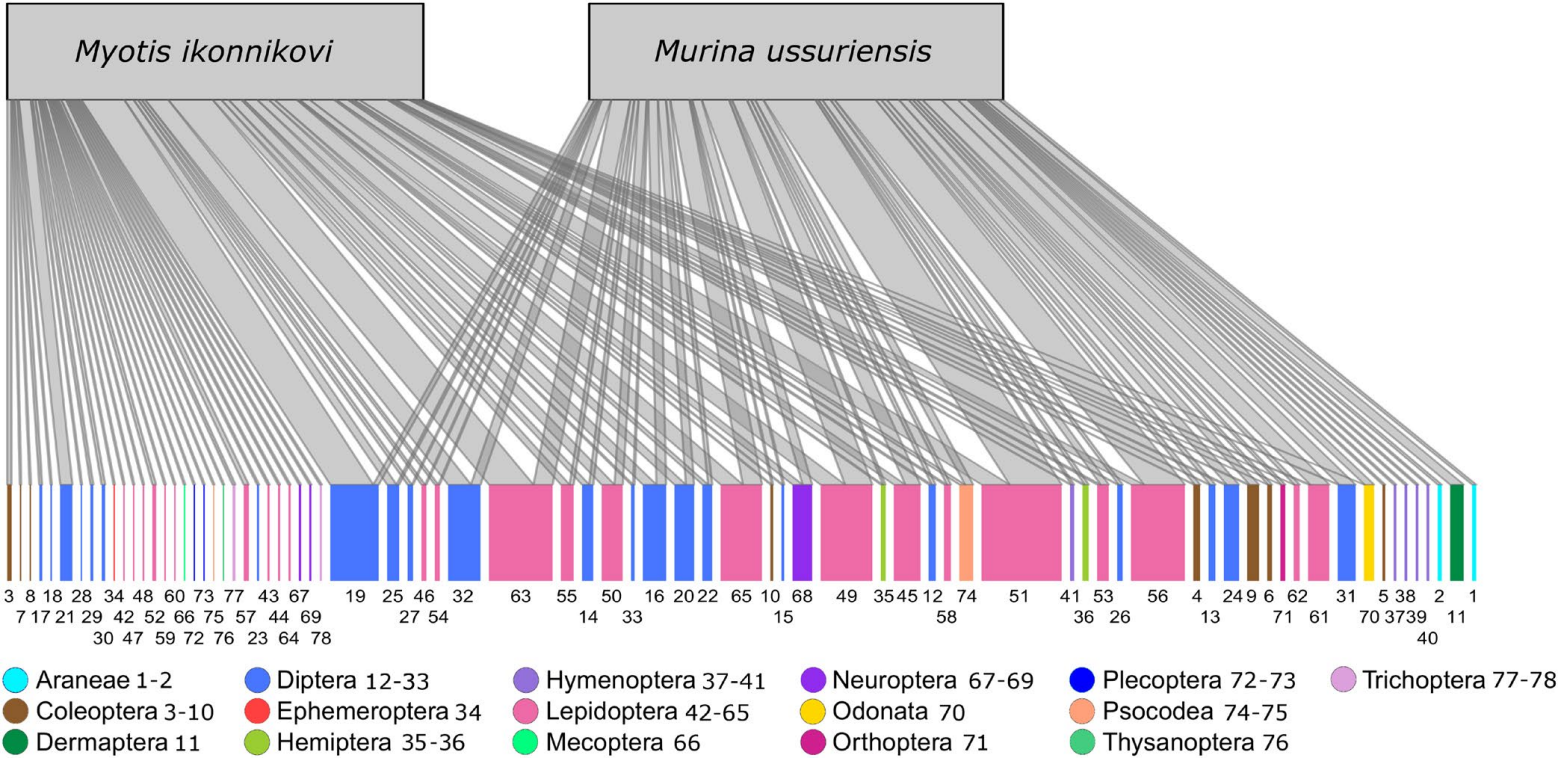
Plotweb depicts how prey items from various families (legend for numbers is given in Appendix S5: A8) and orders were partitioned between the “core” diets of *Myotis ikonnikovi* and *Murina ussuriensis*. Note that the number of shared families and orders in this graph is slightly higher than in the Venn diagram (Figure 5) as prey items that were consumed by bat 1 just once were taken into account in cases where the same prey item was consumed by bat 2 more than once

We found that the core diet *M*. *ikonnikovi* often contained more prey from the families Limoniidae (19), Tipulidae (32), and Tortricidae (63) than that of *M*. *ussuriensis* (Figure 6) across the insect families of Diptera and Lepidoptera from which both bat species shared prey items. In contrast, prey from the families Phoridae (24), Geometridae (51), Noctuidae (56), and Saturniidae (61) occurred more frequently in the core diet of *M*. *ussuriensis* compared to that of *M*. *ikonnikovi*. Finally, both bat species shared approximately equal frequencies of the following prey families (ordered in decreasing occurrence): Erebidae (49), Crambidae (45), Hemerobiidae (68), Pediciidae (22), Anthomyiidae (12), Notodontindae (58), Miridae (35), Tenebrionidae (10), Chloropidae (15, Figure 6). Bats consumed the same prey item or species in only 18 out of 73 cases within these families.

### 3.5 Predominant mode of foraging

**FIGURE 7:**
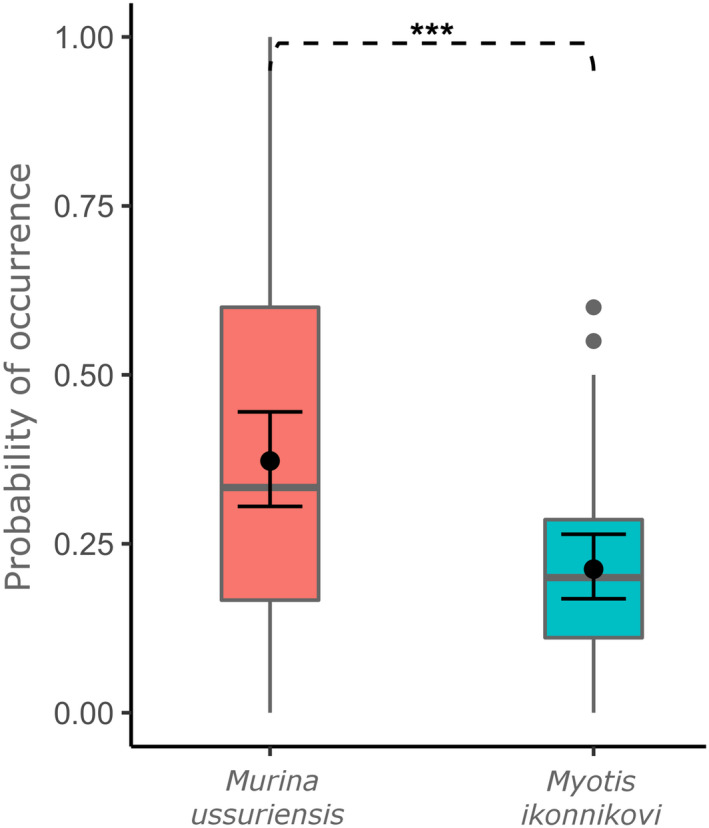
Probability of prey items that were likely caught via gleaning (probability of 1) or aerial hawking (probability of 0) to occur in the diets of *Murina ussuriensis* and *Myotis ikonnikovi*. The boxplots represent the raw data of proportions of prey items that were likely caught via gleaning in relation to aerial hawking, while the filled circles and 95% confidence intervals represent the values obtained from the generalized liner mixed effect model

The residuals of the fitted generalized linear mixed effect model showed no signs of model assumption violations. The model explained significantly more variance than the respective null model (pb‐test statistic = 13.7, df = 1, *p* = .002), and the factor of the bat species was also found to have an effect (*χ* ^2^_Type II Wald_ = 14.4, df = 1, *p* = .0001). In particular, we found that prey items that were most likely caught via gleaning had a higher probability of occurring in the diet of *M*. *ussuriensis* with 37.3 ± 3.6% than in the diet of *M*. *ikonnikovi* with a probability of 21.3 ± 2.4% (odds‐ratio = 2.2 ± 0.46 SE, *z*‐ratio = −3.79, *p* = .0001, Figure 7).

Furthermore, our check on whether prey items from the order of Lepidoptera were consumed as adults or caterpillars (Appendix S5: A5) indicated that most prey items were consumed as adults by both bat species.

### 3.6 Consumed pest insects

A total of 4 and 57 prey items from the orders of Coleoptera and Lepidoptera, respectively, have been identified at the species level. Out of these, we found 21 species that are considered pest insects mainly within the family of Tortricidae (*n* = 9) (please find further details in the Appendix S4).

## 4 DISCUSSION

The present work is the first one, to our knowledge, to study the diet composition of *M*. *ikonnikovi* and *M*. *ussuriensis* via DNA metabarcoding using fecal samples. This study's aim was to elucidate the diet composition of two forest‐dwelling bat species *M*. *ikonnikovi* and *M*. *ussuriensis* and to investigate to what degree these diets overlap.

We found a higher prey diversity in the diet of *M*. *ikonnikovi* compared to the diet of *M*. *ussuriensis* even though the diet of *M*. *ikonnikovi* was represented by a lower sample size. Furthermore, both species most often consumed prey from the orders Lepidoptera and Diptera, and *M*. *ikonnikovi* showed a higher proportion of prey belonging to the order Diptera in its diet than *M*. *ussuriensis*. Those families that belong to the order of Diptera and that were most frequently consumed by *M*. *ikonnikovi* were, interestingly, those that represent insect species with aquatic life stages (e.g., Limoniidae, Tipulidae) (Capinera, 2008). In contrast, we found prey items from various Lepidopteran families in the diet of *M*. *ussuriensis*, while prey from the order of Diptera was less often consumed. The functional analysis suggested that prey that is more likely to be captured in a gleaning mode had a higher probability to occur in the diet of *M*. *ussuriensis* as compared to *M*. *ikonnikovi*. Furthermore, infrequently consumed prey items from orders and families like Araneae, Forficulidae, and Scarabaeidae that represent arthropods with no or low flight capabilities (Capinera, 2008) or prey from families like Libellulidae that represent rather diurnal insects (Heckman, 2006) occurred in the diet of *M*. *ussuriensis*, which might indicate occasional foraging in a gleaning mode—either from the ground or from leaf surfaces. However, the limitations of the functional classification of prey items should be kept in mind when interpreting the results. Another indication of gleaning as the dominant style of foraging is a relatively frequent consumption of caterpillars, as was found in previous studies (Kalka & Kalko, 2006; Wilson & Barclay, 2006). Based on phenological data (Appendix S5: A5), we estimated whether the identified species of Lepidoptera in the diets of both bat species were captured as adults or as caterpillars and found that the majority of those prey items were most probably consumed as adults by both bat species.

Taken together, these findings might indicate that *M*. *ikonnikovi* is a more generalist predator compared to *M*. *ussuriensis*. We further infer that both bat species forage predominantly in aerial‐hawking mode, while *M*. *ussuriensis* might occasionally switch to gleaning mode.

Due to the rarity of dietary studies on the investigated bat species using molecular methods, we attempted to set our results into the context of studies using morphological methods on either the same bat species or ecologically similar bat species. A relatively recent study from Sato and Katsuta (2018) investigated the diet composition of several Japanese bat species by dissecting fecal pellets under a stereomicroscope and identifying single insect fragments up to the species level. In summary, Coleoptera occurred far more frequently in the diet of the bats investigated by Sato and Katsuta (2018) compared to the bats that were investigated in the present study. This difference might be due to a bias of the morphological method that Sato and Katsuta (2018) used, as more sclerotized prey parts especially from Coleoptera, remain intact after digestion to a stronger degree than soft‐bodied prey (Rabinowitz & Tuttle, 1982). Sato and Katsuta (2018), despite the methodological differences, sampled bats from a wider area (prefectures of Tohoku, Kanto, and Chubu) and from a wider time frame (from May to October across 4 to 6 years) compared to the present study that focused on samples from Hokkaido forests.

Compared to the diet composition of European forest‐dwelling and foliage‐gleaning bat species investigated in forested habitats by Andreas et al. (2012), the diet composition of *M*. *ussuriensis* seems rather to resemble the diet composition of *Myotis bechsteinii*, which combines both foraging modes, and not the one of *Myotis myotis*, which predominantly gleans arthropods such as beetles and crickets from the ground (Arlettaz et al., 1997; Pereira et al., 2002). Therefore, our results appear to be in agreement with previously made observations on ecologically similar bat species. Finally, it is important to mention the potential significance of the ecosystem service that both bat species provide: We found about 20 species of Lepidoptera that are considered pests of crops such as tea (e.g., *Ectropis oblique*) and of trees (e.g., species from genera *Lymantria*, *Spodoptera* and *Saturnia*, Appendix S4).

## 5 CONCLUSION

The present study contributed significantly to the current knowledge of the diet composition of *M*. *ussuriensis* and *M*. *ikonnikovi*. We have also uncovered to what degree the diet composition of both bat species overlapped. Furthermore, the bat species with the lower aspect ratio and more rounded wing tips is likely to forage in greater extent by gleaning compared to the other bat species. We encourage further studies on the diet composition of these two bat species that cover a larger range of habitats and seasons to evaluate how flexible these species are regarding their diet composition and how valuable they are with regard to ecosystem services.

## CONFLICT OF INTEREST

All authors approved of the final manuscript version for publication. The authors declare no conflict of interests.

## AUTHOR CONTRIBUTIONS

**Olga Heim:** Conceptualization (supporting); Data curation (equal); Formal analysis (equal); Project administration (supporting); Writing – original draft (lead); Writing – review & editing (equal). **Anna I. E. Puisto:** Formal analysis (supporting); Writing – review & editing (equal). **Ilari E. Sääksjärvi:** Funding acquisition (equal); Resources (equal); Writing – review & editing (equal). **Dai Fukui:** Conceptualization (lead); Data curation (equal); Funding acquisition (equal); Investigation (lead); Methodology (equal); Project administration (lead); Resources (equal); Writing – original draft (supporting); Writing – review & editing (equal). **Eero J. Vesterinen:** Conceptualization (supporting); Data curation (equal); Formal analysis (equal); Funding acquisition (equal); Methodology (equal); Project administration (supporting); Resources (equal); Writing – original draft (supporting); Writing – review & editing (equal).

## Supporting information

Appendix S1

Appendix S2

Appendix S3

Appendix S4

Appendix S5

## Data Availability

Labeled raw reads, OTU tables, and ZOTUs are available in the Dryad Digital Repository: https://doi.org/10.5061/dryad.dz08kprzm.

## References

[ece38472-bib-0001] Alberdi, A. , & Gilbert, M. T. P. (2019). hilldiv: an R package for the integral analysis of diversity based on Hill numbers. bioRxiv. 10.1101/545665

[ece38472-bib-0002] Altschul, S. F. , Gish, W. , Miller, W. , Myers, E. W. , & Lipman, D. J. (1990). Basic local alignment search tool. Journal of Molecular Biology, 215, 403–410. 2231712 10.1016/S0022-2836(05)80360-2

[ece38472-bib-0003] Andreas, M. , Reiter, A. , & Benda, P. (2012). Dietary composition, resource partitioning and trophic niche overlap in three forest foliage‐gleaning bats in Central Europe. Acta Chiropterologica, 14, 335–345.

[ece38472-bib-0004] Arlettaz, R. , Perrin, N. , & Hausser, J. (1997). Trophic resource partitioning and competition between the two sibling bat species *Myotis myotis* and *Myotis blythii* . Journal of Animal Ecology, 66(6), 897–911.

[ece38472-bib-0005] Arrizabalaga‐Escudero, A. , Clare, E. L. , Salsamendi, E. , Alberdi, A. , Garin, I. , Aihartza, J. , & Goiti, U. (2018). Assessing niche partitioning of co‐occurring sibling bat species by DNA metabarcoding. Molecular Ecology, 27, 1273–1283. 29411450 10.1111/mec.14508

[ece38472-bib-0006] Bates, D. , Maechler, M. , Bolker, B. , & Walker, S. (2015). Fitting linear mixed‐effects models using lme4. Journal of Statistical Software, 67, 1–48.

[ece38472-bib-0007] CABI (2021). Crop protection compendium. CAB International. www.cabi.org/cpc

[ece38472-bib-0008] Capinera, J. L. (2008). Encyclopedia of entomology. Springer.

[ece38472-bib-0009] Chao, A. , & Lee, S.‐M. (1992). Estimating the number of classes via sample coverage. Journal of the American Statistical Association, 87, 210–217.

[ece38472-bib-0010] Chen, H. (2018). VennDiagram: Generate high‐resolution venn and euler plots. R package version 1.6.20 ed.

[ece38472-bib-0011] Chesson, P. (2000). Mechanisms of maintenance of species diversity. Annual Review of Ecology and Systematics, 31, 343–366.

[ece38472-bib-0012] Clarke, L. J. , Soubrier, J. , Weyrich, L. S. , & Cooper, A. (2014). Environmental metabarcodes for insects: *in silico* PCR reveals potential for taxonomic bias. Molecular Ecology Resources, 14, 1160–1170. 24751203 10.1111/1755-0998.12265

[ece38472-bib-0013] Colwell, R. K. (2013). EstimateS: Statistical estimation of species richness and shared species from samples, 9. http://purl.oclc.org/estimates

[ece38472-bib-0014] Deagle, B. E. , Thomas, A. C. , McInnes, J. C. , Clarke, L. J. , Vesterinen, E. J. , Clare, E. L. , Kartzinel, T. R. , & Eveson, J. P. (2019). Counting with DNA in metabarcoding studies: How should we convert sequence reads to dietary data? Molecular Ecology, 28, 391–406. 29858539 10.1111/mec.14734 PMC6905394

[ece38472-bib-0015] Dorman, C. F. , Frued, J. , Bluethgen, N. , & Gruber, B. (2009). Indices, graphs and null models: analyzing bipartite ecological networks. The Open Ecology Journal, 2, 7–24.

[ece38472-bib-0016] Dorman, C. F. , Gruber, B. , & Frued, J. (2008). Introducing the bipartite Package: Analysing Ecological Networks. R News, 8, 8–11.

[ece38472-bib-0017] Edgar, R. C. (2010). Search and clustering orders of magnitude faster than BLAST. Bioinformatics, 26, 2460–2461. 20709691 10.1093/bioinformatics/btq461

[ece38472-bib-0018] Edgar, R. C. (2016). UNOISE2: improved error‐correction for Illumina 16S and ITS amplicon sequencing. Biorxiv, 81257v1.

[ece38472-bib-0019] Flanders, J. , Inoue‐Murayama, M. , Rossiter, S. J. , & Hill, D. A. (2016). Female philopatry and limited male‐biased dispersal in the Ussuri tube‐nosed bat, *Murina ussuriensis* . Journal of Mammalogy, 97, 545–553.

[ece38472-bib-0020] Fukui, D. , Agetsuma, N. , & Hill, D. A. (2004). Acoustic identification of eight species of bat (Mammalia: Chiroptera) inhabiting forests of Southern Hokkaido, Japan: Potential for conservation monitoring. Zoological Science, 21, 947–955. 10.2108/zsj.21.947 15459453

[ece38472-bib-0021] Fukui, D. , Hill, D. A. , & Matsumura, S. (2012). Maternity Roosts and Behaviour of the Ussurian Tube‐Nosed Bat *Murina ussuriensis* . Acta Chiropterologica, 14, 93–104.

[ece38472-bib-0022] Fukui, D. , Hill, D. A. , Sun‐Sook, K. , & Han, S.‐H. (2015). Echolocation call structure of fourteen bat species in Korea. Animal Systematics, Evolution and Diversity, 31, 160.

[ece38472-bib-0023] Fukui, D. , Hirao, T. , Murakami, M. , & Hirakawa, H. (2011). Effects of treefall gaps created by windthrow on bat assemblages in a temperate forest. Forest Ecology and Management, 261, 1546–1552.

[ece38472-bib-0024] Fukui, D. , Murakami, M. , Nakano, S. , & Aoi, T. (2006). Effect of emergent aquatic insects on bat foraging in a riparian forest. Journal of Animal Ecology, 75, 1252–1258. 17032357 10.1111/j.1365-2656.2006.01146.x

[ece38472-bib-0025] Fukui, D. , Sano, A. , & Kruskop, S. V. (2019). *Murina ussuriensis*. [Online]. The IUCN Red List of Threatened Species 2019: e.T84562332A22095832. 10.2305/IUCN.UK.2019-3.RLTS.T84562332A22095832.en

[ece38472-bib-0026] Geipel, I. , Lattenkamp, E. Z. , Dixon, M. M. , Wiegrebe, L. , & Page, R. A. (2021). Hearing sensitivity: An underlying mechanism for niche differentiation in gleaning bats. Proceedings of the National Academy of Sciences, 118, e2024943118. 10.1073/pnas.2024943118 PMC8433509 34426521

[ece38472-bib-0027] Gómez‐Llano, M. , Germain, R. M. , Kyogoku, D. , McPeek, M. A. , & Siepielski, A. M. (2021). When Ecology Fails: How Reproductive Interactions Promote Species Coexistence. Trends in Ecology & Evolution, 36, 610–622. 33785182 10.1016/j.tree.2021.03.003

[ece38472-bib-0028] Halekoh, U. , & Højsgaard, S. (2014). A kenward‐roger approximation and parametric bootstrap methods for tests in linear mixed models–the R package pbkrtest. Journal of Statistical Software, 59, 1–30.26917999

[ece38472-bib-0029] Hartig, F. (2020). DHARMa: Residual diagnostics for hierarchical (multi‐level/mixed) regression models. R package version 0.3.3.0. ed.

[ece38472-bib-0030] Heckman, C. W. (2006). Encyclopedia of South American aquatic insects: Odonata‐Anisoptera: illustrated keys to known families, genera, and species in South America. Springer Science & Business Media.

[ece38472-bib-0031] Heim, O. , Puisto, A. I. E. , Fukui, D. , & Vesterinen, E. J. (2019). Molecular Evidence of Bird‐Eating Behavior in Nyctalus Aviator. Acta Ethologica, 22(3), 223–226. 10.1007/s10211-019-00319-5

[ece38472-bib-0032] Hill, D. A. , & Greenaway, F. (2005). Effectiveness of an acoustic lure for surveying bats in British woodlands. Mammal Review, 35, 116–122.

[ece38472-bib-0033] Hill, M. O. (1973). Diversity and evenness: A unifying notation and its consequences. Ecology, 54, 427–432.

[ece38472-bib-0034] Jo, Y. S. (2015). Mammals of Korea: Conservation and management. Texas Tech University.

[ece38472-bib-0035] Kalka, M. , & Kalko, E. K. (2006). Gleaning bats as underestimated predators of herbivorous insects: diet of *Micronycteris microtis* (Phyllostomidae) in Panama. Journal of Tropical Ecology, 22, 1–10.

[ece38472-bib-0036] Kaunisto, K. M. , Roslin, T. , Forbes, M. R. , Morrill, A. , Sääksjärvi, I. E. , Puisto, A. I. E. , Lilley, T. M. , & Vesterinen, E. J. (2020). Threats from the air: Damselfly predation on diverse prey taxa. Journal of Animal Ecology, 89, 1365–1374. 32124439 10.1111/1365-2656.13184

[ece38472-bib-0037] Kaunisto, K. M. , Roslin, T. , Sääksjärvi, I. E. , & Vesterinen, E. J. (2017). Pellets of proof: First glimpse of the dietary composition of adult odonates as revealed by metabarcoding of feces. Ecology and Evolution, 7(20), 8588–8598. 10.1002/ece3.3404 29075474 PMC5648679

[ece38472-bib-0038] Kearse, M. , Moir, R. , Wilson, A. , Stones‐Havas, S. , Cheung, M. , Sturrock, S. , Buxton, S. , Cooper, A. , Markowitz, S. , Duran, C. , Thierer, T. , Ashton, B. , Meintjes, P. , & Drummond, A. (2012). Geneious Basic: an integrated and extendable desktop software platform for the organization and analysis of sequence data. Bioinformatics, 28, 1647–1649. 10.1093/bioinformatics/bts199 22543367 PMC3371832

[ece38472-bib-0039] Kim, S.‐S. , Fukui, D. , Han, S.‐H. , Hur, W.‐H. , & Oh, D.‐S. (2014). Habitat Characteristics of *Myotis ikonnikovi* . Korean Journal of Ecology and Environment, 47(1), 41–52.

[ece38472-bib-0040] Lee, S.‐M. , & Chao, A. (1994). Estimating population size via sample coverage for closed capture‐recapture models. Biometrics, 50, 88–97. 19480084

[ece38472-bib-0041] Lenth, R. (2019). emmeans: Estimated marginal means, aka least‐squares means.

[ece38472-bib-0042] Martin, M. (2011). Cutadapt removes adapter sequences from high‐throughput sequencing reads. Embnet Journal, 17, 10.

[ece38472-bib-0043] Neuweiler, G. (1989). Foraging ecology and audition in echolocating bats. Trends in Ecology & Evolution, 4, 160–166. 21227342 10.1016/0169-5347(89)90120-1

[ece38472-bib-0044] Norberg, U. M. , & Rayner, J. M. V. (1987). Ecological Morphology and Flight in Bats (Mammalia; Chiroptera): Wing Adaptations, Flight Performance, Foraging Strategy and Echolocation. Philosophical Transactions of the Royal Society B, 316(1179), 335–427. 10.1098/rstb.1987.0030

[ece38472-bib-0045] Novella‐Fernandez, R. , Ibañez, C. , Juste, J. , Clare, E. L. , Doncaster, C. P. , & Razgour, O. (2020). Trophic resource partitioning drives fine‐scale coexistence in cryptic bat species. Ecology and Evolution, 10, 14122–14136. 33391705 10.1002/ece3.7004 PMC7771180

[ece38472-bib-0046] Ohdachi, S. D. , Ishibashi, Y. , Iwasa, M. A. , Fukui, D. , & Saitoh, T. (2015). The wild mammals of Japan, 2nd edn. Shoukadoh Book Sellers Kyoto.

[ece38472-bib-0047] Pereira, M. J. R. , Rebelo, H. , Rainho, A. , & Palmeirim, J. M. (2002). Prey selection by *Myotis myotis* (Vespertilionidae) in a Mediterranean region. Acta Chiropterologica, 4, 183–193.

[ece38472-bib-0048] Pompanon, F. , Deagle, B. E. , Symondson, W. O. C. , Brown, D. S. , Jarman, S. N. , & Taberlet, P. (2012). Who is eating what: diet assessment using next generation sequencing. Molecular Ecology, 21, 1931–1950. 22171763 10.1111/j.1365-294X.2011.05403.x

[ece38472-bib-0049] R Core Team (2020). R: A language and environment for statistical computing. , 3.6.1 edn. R Foundation for Statistical Computing.

[ece38472-bib-0050] Rabinowitz, A. R. , & Tuttle, M. D. (1982). A test of the validity of two currently used methods of determining bat prey preferences. Acta Theriologica, 27, 283–293.

[ece38472-bib-0051] Ratnasingham, S. , & Hebert, P. (2007). BOLD: The barcode of life data system (www.barcodinglife.org). Molecular Ecology Notes, 7, 355–364. 18784790 10.1111/j.1471-8286.2007.01678.x PMC1890991

[ece38472-bib-0052] Roeleke, M. , Johannsen, L. , & Voigt, C. C. (2018). How Bats Escape the Competitive Exclusion Principle—Seasonal Shift From Intraspecific to Interspecific Competition Drives Space Use in a Bat Ensemble. Frontiers in Ecology and Evolution, 6, 101. 10.3389/fevo.2018.00101

[ece38472-bib-0053] Rognes, T. , Flouri, T. , Nichols, B. , Quince, C. , & Mahé, F. (2016). VSEARCH: a versatile open source tool for metagenomics. PeerJ, 4, e2584. 10.7717/peerj.2584 27781170 PMC5075697

[ece38472-bib-0054] Sato, A. , & Katsuta, S. (2018). Food habits of 16 species of bats in Japan. Japanese Wildlife Research Society, 43, 55–73.

[ece38472-bib-0055] Sato, A. , Katsuta, S. , & Yamamoto, T. (2011). The distribution and roost use of the Ikonnikov's Myotis, *Myotis ikonnikovi*, in the Minami‐Alps area. Journal of the Japanese Wildlife Research Society, 36, 1–7.

[ece38472-bib-0056] Schnitzler, H. U. , Moss, C. F. , & Denzinger, A. (2003). From spatial orientation to food acquisition in echolocating bats. Trends in Ecology & Evolution, 18, 386–394.

[ece38472-bib-0057] Siemers, B. M. , & Schnitzler, H.‐U. (2004). Echolocation signals reflect niche differentiation in five sympatric congeneric bat species. Nature, 429, 657–661. 15190352 10.1038/nature02547

[ece38472-bib-0058] Sorvari, J. , Härkönen, S. K. , & Vesterinen, E. J. (2012). First record of an indoor pest sawtoothed grain beetle Oryzaephilus surinamensis (Coleoptera: Silvanidae) from wild outdoor wood ant nest. Entomologica Fennica, 23(2), 69–71. https://doi.org/10.33338/ef.6777

[ece38472-bib-0059] Stubbe, M. , Ariunbold, J. , Buuveibaatar, V. , Dorjderem, S. , Monkhzul, T. , Otgonbaatar, M. , & Tsogbadrakh, M. (2008). *Myotis ikonnikovi* . 10.2305/IUCN.UK.2008.RLTS.T14168A4414060.en

[ece38472-bib-0060] Sugai, S. , Kondo, N. , Souma, K. , & Masuko, T. (2011). Bat fauna in three river basins originating in Mt. Motoko, Hokkaido. Journal of Agricultural Science Tokyo University of Agriculture, 56, 155–161.

[ece38472-bib-0061] Vesterinen, E. J. , Ruokolainen, L. , Wahlberg, N. , Peña, C. , Roslin, T. , & Laine, V. N. (2016). What you need is what you eat? Prey selection by the bat *Myotis daubentonii* . Molecular Ecology, 25, 1581–1594. 26841188 10.1111/mec.13564

[ece38472-bib-0062] Walker, F. M. , Williamson, C. H. D. , Sanchez, D. E. , Sobek, C. J. , & Chambers, C. L. (2016). Species From Feces: Order‐Wide Identification of Chiroptera From Guano and Other Non‐Invasive Genetic Samples. PLoS One, 11, e0162342. 10.1371/journal.pone.0162342 27654850 PMC5031397

[ece38472-bib-0063] Wilson, J. M. , & Barclay, R. M. R. (2006). Consumption of caterpillars by bats during an outbreak of Western Spruce Budworm. The American Midland Naturalist, 155(244–249), 6.

[ece38472-bib-0064] Zeale, M. R. K. , Butlin, R. K. , Barker, G. L. A. , Lees, D. C. , & Jones, G. (2011). Taxon‐specific PCR for DNA barcoding arthropod prey in bat faeces. Molecular Ecology Resources, 11, 236–244. 21429129 10.1111/j.1755-0998.2010.02920.x

[ece38472-bib-0065] Zhang, J. (2016). spaa: Species association analysis, 0.2.2 edn.

[ece38472-bib-0066] Zhigalin, A. , Stubbe, M. , Ariunbold, J. , Buuveibaatar, V. , Dorjderem, S. , Monkhzul, T. , Otgonbaatar, M. , & Tsogbadrakh, M. (2020). *Myotis ikonnikovi* . The IUCN Red List of Threatened Species.

