# Supplementary material for: Dietary analysis reveals differences in the prey use of two sympatric bat species
Source: Ecol Evol. 2021 Dec 16;11(24):18651–61. doi: 10.1002/ece3.8472 (PMC8717349; doi:10.1002/ece3.8472)
Supplement: Supplementary file 1 — Appendix S1 [file ECE3-11-18651-s004.pdf]

Supplementary information S1 for *Dietary analysis reveals differences in the prey use of two sympatric bat species*

Olga Heim[1], Anna I.E. Puisto[2], Ilari Sääksjärvi[3], Dai Fukui[4] and Eero J. Vesterinen[5]*

1 Faculty of Life and Medical Sciences, Doshisha University, 610-0321 Kyotanabe, Japan

2 Centre for Population Health Research, University of Turku, 20014 Turku, Finland

3 Biodiversity Unit, University of Turku, 20014 Turku, Finland

4 The University of Tokyo Hokkaido Forest, The University of Tokyo, 079-1563 Furano, Japan

5 Department of Biology, University of Turku, 20014 Turku, Finland

Prey list

in Japan	in Hokkaido							Literature sources regarding occurrence of prey item in Hokkaido and/or Japan			
Primer	Class	Order	Family	Subfamily	Genus	Species	BIN	Family	Subfamily	Genus	Species
COI	Arachnida	Araneae	Lycosidae	NA	Pardosa		BOLD:ABA5238		NA	Nadolny et al. 2016	
COI	Arachnida	Araneae	Salticidae				BOLD:AAV1597	Ono et al. 2009			
COI	Arachnida	Araneae	Salticidae				GBCH10991-13	Yaginuma 1972			
COI	Arachnida	Araneae	Salticidae				BOLD:ACH5240	Ono et al. 2009			
COI	Collembola	Symphypleona	Bourletiellidae	NA	Deuterosminthurus		BOLD:AAB7915		NA	Yosii 1977	
COI	Insecta	Blattodea	Ectobiidae	Ectobiinae			BOLD:ACI8100	小松 et al. 2015; 小松 2019	Becalloni 2014; 1		
COI	Insecta	Coleoptera	Carabidae				BOLD:AAZ4621	Kaizuka & Iwasa 2015			
COI	Insecta	Coleoptera	Coccinellidae				BOLD:ACP4109	Okuda & Hodek 1994			
COI	Insecta	Coleoptera	Cantharidae	Cantharinae	Podabrus		BOLD:ACD6222			Kazantsev 2011	
COI	Insecta	Coleoptera	Cantharidae	Cantharinae	Podabrus		BOLD:ACD6257			Kazantsev 2011	
COI	Insecta	Coleoptera	Carabidae	Harpalinae			BOLD:ABX4019		Kaizuka & Iwasa 2015		
COI	Insecta	Coleoptera	Carabidae	Carabinae	Carabus		BOLD:ABY6003			Löbl & Löbl 2017	
COI	Insecta	Coleoptera	Carabidae	Harpalinae	Pterostichus	oblongopunctatus	BOLD:ABY4764			Kosuda et al. 2016	Roskov et al. 2019; 2
COI	Insecta	Coleoptera	Cerambycidae	Lamiinae	Monochamus		BOLD:AAC3388			Togashi et al. 2010	
COI	Insecta	Coleoptera	Chrysomelidae	Chrysomelinae			BOLD:AAC0432		Cho & Świętojańska 2017		
COI	Insecta	Coleoptera	Cryptophagidae	Atomariinae	Atomaria	turgida	BOLD:AAJ9463	Otero et al. 2017	Otero et al. 2017	Otero et al. 2017	Lyubarsky 2014
COI	Insecta	Coleoptera	Curculionidae	Entiminae	Polydrusus		BOLD:ACO8630			Morimoto et al. 2015	
COI	Insecta	Coleoptera	Hydrophilidae	Hydrophilinae	Enochrus		BOLD:AAF0204			Minoshima 2019; Jia & Wang 2010	
COI	Insecta	Coleoptera	Nitidulidae	Nitidulinae	Cychramus	variegatus	BOLD:ABW4745	Kashizaki & Hisamatsu 2011	Lee et al. 2017	Hayashi 1978	Hayashi 1978
COI	Insecta	Coleoptera	Staphylinidae	Euaesthetinae	Euaesthetus		BOLD:ACG9244	Oh & Cho 2015	Puthz 2010	Puthz 2010	
COI	Insecta	Diptera	Anthomyiidae				BOLD:AAG2490	Suwa 2018			
COI	Insecta	Diptera	Tachinidae	Exoristinae			BOLD:AAI6325		O'Hara et al. 2019		
COI	Insecta	Diptera	Tipulidae	Tipulinae	Tipula		BOLD:AAN5045			Men et al. 2019	
COI	Insecta	Diptera	Tipulidae	Tipulinae	Tipula		BOLD:AAN9029			Men et al. 2019	
COI	Insecta	Diptera	Limoniidae	Chioneinae	Teucholabis		BOLD:AAV2508			Kato 2019	
COI	Insecta	Diptera	Limoniidae	Chioneinae			BOLD:ABA4102		Kato 2019		
COI	Insecta	Diptera	Cecidomyiidae				BOLD:ABV2094	Abe et al. 2011			
COI	Insecta	Diptera	Limoniidae				BOLD:ABX4369	Savchenko et al. 1992			
COI	Insecta	Diptera	Limoniidae	Limoniinae	Libnotes		BOLD:ABY2385			Savchenko et al. 1992	

Primer	Class	Order	Family	Subfamily	Genus	Species	BIN	Family	Subfamily	Genus	Species
COI	Insecta	Diptera	Syrphidae	Eristalinae			BOLD:ABY2389		Prokhorov et al. 2018		
COI	Insecta	Diptera	Tipulidae	Tipulinae	Tipula		BOLD:ABY5691			Men et al. 2019	
COI	Insecta	Diptera	Phoridae				BOLD:ACA3150	Michailovskaya 1998			
COI	Insecta	Diptera	Tachinidae	Tachininae	Nemoraea	takanoi	BOLD:ACA7375				O'Hara et al. 2019
COI	Insecta	Diptera	Chironomidae	Chironominae	Glyptotendipes		BOLD:ACE3296			Yamamoto 1996	
COI	Insecta	Diptera	Tachinidae				BOLD:ACF0578	O'Hara et al. 2019			
COI	Insecta	Diptera	Tipulidae	Tipulinae	Tipula		BOLD:ACF0878			Men et al. 2019	
COI	Insecta	Diptera	Phoridae				BOLD:ACF6080	Michailovskaya 1998			
COI	Insecta	Diptera	Sphaeroceridae	Limosininae	Rachispoda		BOLD:ACK2192			Hayashi 1986	
COI	Insecta	Diptera	Chironomidae				BOLD:ACK8680	Sasa & Suzuki 2000, Yamamoto et al. 2015			
COI	Insecta	Diptera	Sciaridae				BOLD:ACM6077	Menzel & Mohrig 2000			
COI	Insecta	Diptera	Simuliidae				BOLD:ACM8222	Adler & Crosskey 2008			
COI	Insecta	Diptera	Limoniidae				BOLD:ACN0178	Savchenko et al. 1992			
COI	Insecta	Diptera	Limoniidae				BOLD:ACN0238	Savchenko et al. 1992			
COI	Insecta	Diptera	Mycetophilidae				BOLD:ACN0859	Ratnasingham & Hebert 2007			
COI	Insecta	Diptera	Calliphoridae				BOLD:ACN1515	Hori et al. 1990			
COI	Insecta	Diptera	Psychodidae				BOLD:ACN3276	Sanjoba et al. 2011			
COI	Insecta	Diptera	Drosophilidae				BOLD:ACN4805	Toda 1987			
COI	Insecta	Diptera					BOLD:ACN5240				
COI	Insecta	Diptera	Limoniidae				BOLD:ACN5325	Savchenko et al. 1992			
COI	Insecta	Diptera	Phoridae				BOLD:ACO0272	Michailovskaya 1998			
COI	Insecta	Diptera	Chironomidae				BOLD:ACO4488	Sasa & Suzuki 2000, Yamamoto et al. 2015			
COI	Insecta	Diptera					BOLD:ACP6542				
COI	Insecta	Diptera	Chloropidae				BOLD:ACR0489	Nartshuk 2017			
COI	Insecta	Diptera	Muscidae				BOLD:ACT7328	Ratnasingham & Hebert 2007			
COI	Insecta	Diptera	Sciaridae				BOLD:ACU8386	Menzel & Mohrig 2000			
COI	Insecta	Diptera	Muscidae	Phaoniinae	Helina		BOLD:ACV2533			Zhang & Kurahashi 2000	
COI	Insecta	Diptera	Psychodidae				BOLD:ACW2931	Sanjoba et al. 2011			
COI	Insecta	Diptera	Agromyzidae				BOLD:AAV4874	Sasakawa 2015			
COI	Insecta	Diptera	Anthomyiidae	Anthomyiinae	Botanophila		BOLD:AAG2502			Sevcik & Papp 2004	
COI	Insecta	Diptera	Anthomyiidae	Anthomyiinae	Lasiomma		BOLD:ACK1581			Suwa 2018	
COI	Insecta	Diptera	Anthomyiidae	Anthomyiinae	Delia	platura	BOLD:AAG2511				Mizukoshi 2000
COI	Insecta	Diptera	Anthomyiidae	Anthomyiinae	Botanophila	profuga	BOLD:ACP6334				Suwa 1999
COI	Insecta	Diptera	Bolitophilidae	NA	Bolitophila		BOLD:AAM9007		NA	Sevcik & Papp 2004	
COI	Insecta	Diptera	Cecidomyiidae				BOLD:ACP9491	Abe et al. 2011			
COI	Insecta	Diptera	Cecidomyiidae				BOLD:ACU9836	Abe et al. 2011			
COI	Insecta	Diptera	Cecidomyiidae	Cecidomyiinae	Feltiella	acarisuga	BOLD:ACD4136				Abe et al. 2011

Primer	Class	Order	Family	Subfamily	Genus	Species	BIN	Family	Subfamily	Genus	Species
COI	Insecta	Diptera	Chamaemyiidae				BOLD:ACC5885	Nagatomi 1968			
COI	Insecta	Diptera	Chironomidae	Diamesinae	Diamesa		BOLD:AAB5106			Suzuki 1998	
COI	Insecta	Diptera	Chironomidae	Orthocladiinae	Heterotrissocladius	marcidus	BOLD:AAF2163		Sasa & Suzuki 2000, Yamamoto et al. 2015	Yamamoto 2004	Yamamoto 2004
COI	Insecta	Diptera	Chironomidae	Orthocladiinae	Eukiefferiella		BOLD:AAI5126			Sasa & Suzuki 2000	
COI	Insecta	Diptera					BOLD:ACQ9269				
COI	Insecta	Diptera	Chironomidae	Orthocladiinae	Cricotopus	bicinctus	BOLD:AAT9677				Sasa & Suzuki 2001
COI	Insecta	Diptera	Chironomidae				BOLD:ACB8882	Sasa & Suzuki 2000, Yamamoto et al. 2015			
COI	Insecta	Diptera	Chironomidae				BOLD:ACF6427	Sasa & Suzuki 2000, Yamamoto et al. 2015			
COI	Insecta	Diptera	Chironomidae	Diamesinae			BOLD:ACK5496		Ratnasingham & Hebert 2007		
COI	Insecta	Diptera	Chloropidae	Oscinellinae			BOLD:AAN5657		Nartshuk 2017		
COI	Insecta	Diptera	Chloropidae				BOLD:ADC9928	Nartshuk 2017			
COI	Insecta	Diptera	Culicidae	Culicinae			BOLD:AAF2904		Tanaka 1999		
COI	Insecta	Diptera	Culicidae	Culicinae	Culex	orientalis	BOLD:ACB9306				Ratnasingham & Hebert 2007
COI	Insecta	Diptera	Culicidae	Culicinae	Culex		BOLD:AAA4751			Ratnasingham & Hebert 2007; Fonseca et al. 2009	
COI	Insecta	Diptera	Cylindrotomidae	Cylindrotominae	Cylindrotoma		BOLD:AAD0770			Ratnasingham & Hebert 2007	
COI	Insecta	Diptera	Drosophilidae	Drosophilinae	Drosophila		BOLD:AAY0127			Ratnasingham & Hebert 2007	
COI	Insecta	Diptera	Drosophilidae	Drosophilinae	Drosophila		BOLD:ADH1432			Ratnasingham & Hebert 2007	
COI	Insecta	Diptera	Drosophilidae	Steganinae	Leucophenga		BOLD:ACA1411			Chen & Aotsuka 2003	
COI	Insecta	Diptera	Ephydridae				BOLD:ACS0447	Kang & Suh 2017			
COI	Insecta	Diptera	Hybotidae	Tachydromiinae	Chersodromia		BOLD:ACJ9160			Maeda 2011	
COI	Insecta	Diptera	Keroplatidae				BOLD:AAG4943	Polevoi & Barkalov 2017			
COI	Insecta	Diptera	Limoniidae	Limoniinae	Antocha	bifida	BOLD:AAW5955				Savchenko et al. 1992
COI	Insecta	Diptera	Limoniidae	Limoniinae	Dicranomyia	frontalis	BOLD:ABA5203				Savchenko et al. 1992
COI	Insecta	Diptera	Limoniidae	Limoniinae	Dicranomyia		BOLD:ABW0212			Savchenko et al. 1992	
COI	Insecta	Diptera	Limoniidae				BOLD:ABV4791	Savchenko et al. 1992			
COI	Insecta	Diptera	Limoniidae				BOLD:ACS9407	Savchenko et al. 1992			
COI	Insecta	Diptera	Limoniidae	Limoniinae	Discobola		BOLD:ABW4922			Savchenko et al. 1992	
COI	Insecta	Diptera	Limoniidae	Limoniinae	Libnotes		BOLD:ABW9478			Savchenko et al. 1992	
COI	Insecta	Diptera	Limoniidae	Limoniinae	Dicranomyia		BOLD:AAO3939			Savchenko et al. 1992	
COI	Insecta	Diptera	Limoniidae	Limoniinae	Dicranomyia		FINTI487-12			Savchenko et al. 1992	
COI	Insecta	Diptera	Limoniidae	Limoniinae	Metalimnobia		BOLD:ABU9381			Savchenko et al. 1992	
COI	Insecta	Diptera	Limoniidae	Limoniinae	Metalimnobia		BOLD:AAX5093			Savchenko et al. 1992	
COI	Insecta	Diptera	Limoniidae	Limoniinae	Rhipidia	maculata	BOLD:ACB7799				Savchenko et al. 1992
COI	Insecta	Diptera	Lonchopteridae	NA	Lonchoptera		BOLD:ACB6694		NA	Wagner et al. 2008	
COI	Insecta	Diptera	Muscidae				BOLD:ACD2056	Vikhrev 2009; Ratnasingham & Hebert 2007			

Primer	Class	Order	Family	Subfamily	Genus	Species	BIN	Family	Subfamily	Genus	Species
COI	Insecta	Diptera	Muscidae				BOLD:AAG1765	Vikhrev 2009; Ratnasingham & Hebert 2007			
COI	Insecta	Diptera	Muscidae	Phaoniinae	Helina	obscurata	BOLD:AAE6931			Zhang & Kurahashi 2000	Sorokina et al. 2018
COI	Insecta	Diptera	Muscidae	Azeliinae	Muscina	pascuorum	BOLD:AAG1714			Vikhrev 2009	Sawabe et al. 2006
COI	Insecta	Diptera	Muscidae				BOLD:ACR5608	Vikhrev 2009			
COI	Insecta	Diptera	Muscidae				BOLD:AAX1493	Ratnasingham & Hebert 2007; Zhang & Kurahashi 2000; Iwasa 2007			
COI	Insecta	Diptera	Muscidae	Azeliinae	Hydrotaea	armipes	BOLD:AAG6908			Vikhrev 2015	Mihalyi 1976
COI	Insecta	Diptera	Muscidae	Phaoniinae	Phaonia		BOLD:AAG1772			Вихрев & Ерофеева 2018	
COI	Insecta	Diptera	Muscidae	Phaoniinae	Phaonia		BOLD:AAP8142			Вихрев & Ерофеева 2018	
COI	Insecta	Diptera	Muscidae	Azeliinae	Hydrotaea	glabricula	BOLD:ADK1195			Vikhrev 2015	Vikhrev 2015
COI	Insecta	Diptera	Muscidae	Azeliinae	Potamia	littoralis	BOLD:ACQ0523				Iwasa et al. 1995
COI	Insecta	Diptera	Muscidae	Azeliinae	Thricops	diaphanus	BOLD:AAG1710				Beppu 2004
COI	Insecta	Diptera	Mycetophilidae	Mycetophilinae	Platurocypta		BOLD:ACC5656		Ratnasingham & Hebert 2007	杉浦 et al. 2003	
COI	Insecta	Diptera	Pediciidae	Uliinae	Ula		BOLD:AAV1814			Savchenko et al. 1992	
COI	Insecta	Diptera	Pediciidae	Pediciinae	Dicranota		BOLD:ABW5265			Savchenko et al. 1992	
COI	Insecta	Diptera	Pediciidae	Uliinae	Ula	bolitophila	BOLD:ABU5946				Savchenko et al. 1992
COI	Insecta	Diptera	Pediciidae	Uliinae	Ula		BOLD:ABA7462			Savchenko et al. 1992	
COI	Insecta	Diptera	Periscelididae				BOLD:ADE5755	Sueyoshi & Mathis 2004			
COI	Insecta	Diptera	Phoridae				BOLD:AAP6413	Michailovskaya 1998			
COI	Insecta	Diptera	Psychodidae				BOLD:ACO7340	Sanjoba et al. 2011			
COI	Insecta	Diptera	Psychodidae				BOLD:ADE7332	Sanjoba et al. 2011			
COI	Insecta	Diptera	Psychodidae				BOLD:ADE8063	Sanjoba et al. 2011			
COI	Insecta	Diptera	Psychodidae	Psychodinae	Psychoda		BOLD:AAL7819	Sanjoba et al. 2011		Kiyoku 1958; Yones et al. 2013	
COI	Insecta	Diptera	Psychodidae	Psychodinae	Psychoda		BOLD:ACD9559	Sanjoba et al. 2011	Yones et al. 2013	Yones et al. 2013	
COI	Insecta	Diptera	Sarcophagidae				BOLD:ACU3079	Verves & Khrokalo 2006			
COI	Insecta	Diptera	Sciaridae				BOLD:ACC1760	Menzel & Mohrig 2000			
COI	Insecta	Diptera	Sciaridae				BOLD:ACI7364	Menzel & Mohrig 2000			
COI	Insecta	Diptera	Simuliidae	Simuliinae	Simulium		BOLD:ABA1846			Adler & Crosskey 2008	
COI	Insecta	Diptera	Sphaeroceridae	Limosininae	Opalimosina	mirabilis	BOLD:AAN6406				Hayashi 2010
COI	Insecta	Diptera	Syrphidae	Eristalinae	Ferdinandea	cuprea	BOLD:AAJ0402		Prokhorov et al. 2018	Khaghaninia et al., 2014	Khaghaninia et al., 2014
COI	Insecta	Diptera	Tachinidae	Exoristinae	Cyzenis		BOLD:AAP4829			O'Hara et al. 2019	
COI	Insecta	Diptera	Tachinidae	Exoristinae	Drino		BOLD:AAB6198			O'Hara et al. 2019	
COI	Insecta	Diptera	Tachinidae	Exoristinae			BOLD:AAN9266		O'Hara et al. 2019		
COI	Insecta	Diptera	Tachinidae	Exoristinae	Cadurciella	tritaeniata	BOLD:AAN9651				O'Hara et al. 2019
COI	Insecta	Diptera	Tachinidae	Exoristinae	Phebellia	villica	BOLD:ACB0760				O'Hara et al. 2019
COI	Insecta	Diptera	Tachinidae				BOLD:AAZ8670	O'Hara et al. 2019			
COI	Insecta	Diptera	Tachinidae	Exoristinae			BOLD:AAC1692		O'Hara et al. 2019		

Primer	Class	Order	Family	Subfamily	Genus	Species	BIN	Family	Subfamily	Genus	Species
COI	Insecta	Diptera	Tipulidae	Tipulinae	Tipula	kuzuensis	BOLD:AAN9034			Men et al. 2019	Young et al. 2013
COI	Insecta	Diptera	Tipulidae	Tipulinae	Tipula		BOLD:ABV3695			Men et al. 2019	
COI	Insecta	Diptera	Tipulidae	Tipulinae	Tipula		BOLD:ABV4667			Men et al. 2019	
COI	Insecta	Diptera	Tipulidae	Tipulinae	Tipula		TIPTW088-10			Men et al. 2019	
COI	Insecta	Diptera	Tipulidae				TIPTW093-10	Men et al. 2019			
COI	Insecta	Diptera	Trichoceridae	Trichocerinae	Trichocera		BOLD:ACF7745			Krzeminska 2001 (Trichocera sakaguchii is in Japan; has no record in BOLD)	
COI	Insecta	Hemiptera	Pentatomidae				BOLD:AAP3528	Ishikawa & Moriya 2019			
COI	Insecta	Hemiptera	Cicadellidae	Deltocephalinae			BOLD:AAG8956		Luo et al. 2019		
COI	Insecta	Hemiptera	Cicadidae	Cicadinae	Yezoterpnosia	nigricosta	BOLD:ACP9813				中田 et al. 2006
COI	Insecta	Hemiptera	Psyllidae				BOLD:ACM6872	Cho et al. 2019			
COI	Insecta	Hymenoptera	Cimbicidae	Cimbicinae	Trichiosoma		BOLD:ACJ7002			Лелей et al. 2012	
COI	Insecta	Hymenoptera	Cimbicidae	Cimbicinae	Trichiosoma		BOLD:ABZ2163			Лелей et al. 2012	
COI	Insecta	Hymenoptera	Ichneumonidae				BOLD:ADI1279	Watanabe 2015			
COI	Insecta	Hymenoptera	Pamphiliidae	Pamphiliinae	Neurotoma	nemoralis	BOLD:AAV6346				ЛЕЛЕЙ et al. 2016
COI	Insecta	Hymenoptera	Pamphiliidae	Pamphiliinae	Pamphilius		BOLD:AAK4614			Лелей et al. 2012	
COI	Insecta	Hymenoptera					EII067-15				
COI	Insecta	Hymenoptera					EII104-15				
COI	Insecta	Hymenoptera	Tenthredinidae	Nematinae	Pristiphora		BOLD:AAK9450			Лелей et al. 2012	
COI	Insecta	Lepidoptera	Coleophoridae	Coleophorinae	Coleophora		BOLD:AAC8694			Niwaka & Jimbo 2018; 3	
COI	Insecta	Lepidoptera	Crambidae	Scopariinae	Eudonia	persimilis	BOLD:AAF1478				Niwaka & Jimbo 2018; 4
COI	Insecta	Lepidoptera					BOLD:AAF8577				
COI	Insecta	Lepidoptera					BOLD:AAG6034				
COI	Insecta	Lepidoptera	Erebidae	Arctiinae			BOLD:AAH2626		ЛЕЛЕЙ et al. 2016		
COI	Insecta	Lepidoptera	Gelechiidae	Gelechiinae			BOLD:AAH5654		ЛЕЛЕЙ et al. 2016		
COI	Insecta	Lepidoptera	Noctuidae	Noctuinae			BOLD:AAI3519		Sayama et al. 2012; ЛЕЛЕЙ et al. 2016		
COI	Insecta	Lepidoptera	Erebidae				BOLD:AAL4384	Lafontaine & Schmidt 2013			
COI	Insecta	Lepidoptera	Crambidae	Spilomelinae	Pleuroptya/ Patania	expictalis	BOLD:AAL4502				ЛЕЛЕИ et al. 2016; Niwaka & Jimbo 2018; 5
COI	Insecta	Lepidoptera	Erebidae	Boletobiinae	Enispa		BOLD:AAL7095			ЛЕЛЕЙ et al. 2016	
COI	Insecta	Lepidoptera	Noctuidae	Noctuinae			BOLD:AAM0480		Sayama et al. 2012; ЛЕЛЕИ et al. 2016		
COI	Insecta	Lepidoptera	Notodontidae	Notodontinae	Cerura		BOLD:AAM4552			ЛЕЛЕЙ et al. 2016	
COI	Insecta	Lepidoptera	Noctuidae				BOLD:AAM9055	ЛЕЛЕЙ et al. 2016			
COI	Insecta	Lepidoptera	Noctuidae	Noctuinae			BOLD:AAM9761		Sayama et al. 2012; ЛЕЛЕЙ et al. 2016		
COI	Insecta	Lepidoptera	Crambidae	Pyraustinae	Goniorhynchus		BOLD:AAN3555			ЛЕЛЕЙ et al. 2016	
COI	Insecta	Lepidoptera	Hesperiidae				BOLD:AAN7248	ЛЕЛЕЙ et al. 2016			
COI	Insecta	Lepidoptera	Noctuidae				BOLD:AAP1728	ЛЕЛЕЙ et al. 2016			

Primer	Class	Order	Family	Subfamily	Genus	Species	BIN	Family	Subfamily	Genus	Species
COI	Insecta	Lepidoptera	Geometridae	Ennominae			BOLD:AAP2372		Sayama et al. 2012; ЛЕЛЕЙ et al. 2016		
COI	Insecta	Lepidoptera	Gelechiidae				BOLD:AAQ1460	ЛЕЛЕЙ et al. 2016			
COI	Insecta	Lepidoptera	Crambidae	Spilomelinae			BOLD:AAQ2087		ЛЕЛЕЙ et al. 2016		
COI	Insecta	Lepidoptera	Geometridae	Sterrhinae	Scopula		BOLD:AAV8961			Sayama et al. 2012	
COI	Insecta	Lepidoptera	Erebidae	Arctiinae			BOLD:AAY5858		ЛЕЛЕЙ et al. 2016		
COI	Insecta	Lepidoptera	Limacodidae				BOLD:AAZ8422	ЛЕЛЕЙ et al. 2016			
COI	Insecta	Lepidoptera	Tortricidae	Olethreutinae	Rhopobota		BOLD:ABA8634			Sayama et al. 2012	
COI	Insecta	Lepidoptera	Geometridae	Larentiinae	Leptostegna		BOLD:ABU5788			Sayama et al. 2012	
COI	Insecta	Lepidoptera	Sphingidae	Smerinthinae	Marumba	gaschkewitschii	BOLD:ABU7231				Sayama et al. 2012
COI	Insecta	Lepidoptera					BOLD:ABU9226				
COI	Insecta	Lepidoptera	Tortricidae				BOLD:ABW0658	Sayama et al. 2012, ЛЕЛЕЙ et al. 2016			
COI	Insecta	Lepidoptera	Erebidae	Arctiinae	Cyana		BOLD:ABX3752			Sayama et al. 2012	
COI	Insecta	Lepidoptera	Erebidae	Erebinae	Catocala		BOLD:ABZ3190			ЛЕЛЕЙ et al. 2016	
COI	Insecta	Lepidoptera	Erebidae	Arctiinae			BOLD:ACB1726		ЛЕЛЕЙ et al. 2016		
COI	Insecta	Lepidoptera	Geometridae	Geometrinae			BOLD:ACE9755		ЛЕЛЕЙ et al. 2016		
COI	Insecta	Lepidoptera	Autostichidae				BOLD:ACF7812	Park & Kim 2017			
COI	Insecta	Lepidoptera	Cosmopterigidae				BOLD:ACJ1842	ЛЕЛЕЙ et al. 2016			
COI	Insecta	Lepidoptera	Gelechiidae	Gelechiinae	Sophronia		BOLD:ACL1953			ЛЕЛЕЙ et al. 2016	
COI	Insecta	Lepidoptera	Geometridae	Ennominae			BOLD:ACL6993		Sayama et al. 2012; ЛЕЛЕЙ et al. 2016		
COI	Insecta	Lepidoptera					BOLD:ACM3459				
COI	Insecta	Lepidoptera	Elachistidae				BOLD:ACM9615	ЛЕЛЕЙ et al. 2016; Niwaka & Jimbo 2018; 6			
COI	Insecta	Lepidoptera	Tortricidae				BOLD:ACN0142	Sayama et al. 2012, ЛЕЛЕЙ et al. 2016			
COI	Insecta	Lepidoptera	Gelechiidae				BOLD:ACN0185	ЛЕЛЕЙ et al. 2016			
COI	Insecta	Lepidoptera	Tortricidae	Tortricinae	Choristoneura		BOLD:ACN0625			ЛЕЛЕЙ et al. 2016	
COI	Insecta	Lepidoptera	Erebidae	Herminiinae			BOLD:ACO1434		ЛЕЛЕЙ et al. 2016		
COI	Insecta	Lepidoptera	Lasiocampidae	Poecilocampinae			BOLD:ACP2255		ЛЕЛЕЙ et al. 2016; Niwaka & Jimbo 2018; 7		
COI	Insecta	Lepidoptera	Gelechiidae				BOLD:ACR6168	ЛЕЛЕЙ et al. 2016			
COI	Insecta	Lepidoptera	Stathmopodidae	NA	Stathmopoda		BOLD:ACV9615		NA	ЛЕЛЕЙ et al. 2016; Niwaka & Jimbo 2018; 8	
COI	Insecta	Lepidoptera					BOLD:ACW1375				
COI	Insecta	Lepidoptera					BOLD:ACX5590				
COI	Insecta	Lepidoptera	Crambidae				BOLD:ACX6648	ЛЕЛЕЙ et al. 2016			
COI	Insecta	Lepidoptera					BOLD:ADF8756				
COI	Insecta	Lepidoptera					BOLD:ADG3051				
COI	Insecta	Lepidoptera					BOLD:ADI1550				

Primer	Class	Order	Family	Subfamily	Genus	Species	BIN	Family	Subfamily	Genus	Species
COI	Insecta	Lepidoptera	Tortricidae	Tortricinae	Acleris	paradiseana	BOLD:ADI2826				ЛЕЛЕЙ et al. 2016
COI	Insecta	Lepidoptera					BOLD:ADI2961				
COI	Insecta	Lepidoptera					BOLD:ADI4331				
COI	Insecta	Lepidoptera	Geometridae	Sterrhinae	Scopula		BOLD:ADI7455			Sayama et al. 2012	
COI	Insecta	Lepidoptera	Geometridae	Sterrhinae	Scopula		BOLD:ADJ2221			Sayama et al. 2012	
COI	Insecta	Lepidoptera					GWOSS664-11				
COI	Insecta	Lepidoptera	Geometridae	Geometrinae			NAGEO164-09		Sayama et al. 2012		
COI	Insecta	Lepidoptera	Autostichidae	Autostichinae			BOLD:AAC7842	Park & Kim 2017	Park & Kim 2017		
COI	Insecta	Lepidoptera	Blastobasidae	Blastobasinae	Hypatopa		BOLD:AAB9632			ЛЕЛЕЙ et al. 2016; Niwaka & Jimbo 2018; 9	
COI	Insecta	Lepidoptera	Blastobasidae	Blastobasinae			BOLD:AAX6193		ЛЕЛЕЙ et al. 2016		
COI	Insecta	Lepidoptera	Coleophoridae	Coleophorinae	Coleophora		BOLD:AAB8487			Niwaka & Jimbo 2018; 10	
COI	Insecta	Lepidoptera	Crambidae	Pyraustinae	Pyrausta/ Goniorhynchus		BOLD:AAD0032			ЛЕЛЕЙ et al. 2016; Niwaka & Jimbo 2018; 11	
COI	Insecta	Lepidoptera					BOLD:ACM1938				
COI	Insecta	Lepidoptera	Crambidae	Acentropinae	Nymphula/Elophila		BOLD:AAF2874			ЛЕЛЕЙ et al. 2016; Chen et al. 2010	
COI	Insecta	Lepidoptera	Crambidae	Spilomelinae	Palpita		BOLD:AAD6468			Sayama et al. 2012	
COI	Insecta	Lepidoptera	Crambidae	Spilomelinae			BOLD:AAB6255		ЛЕЛЕЙ et al. 2016; Niwaka & Jimbo 2018; 12		
COI	Insecta	Lepidoptera	Crambidae	Spilomelinae	Pleuroptya/Patania		BOLD:AAB6257			ЛЕЛЕЙ et al. 2016	
COI	Insecta	Lepidoptera	Depressariidae				BOLD:AAY5033	ЛЕЛЕЙ et al. 2016			
COI	Insecta	Lepidoptera	Depressariidae	Depressariinae	Agonopterix		BOLD:AAF7176			ЛЕЛЕЙ et al. 2016	
COI	Insecta	Lepidoptera	Depressariidae	Depressariinae	Depressaria		BOLD:ACN1500			ЛЕЛЕЙ et al. 2016	
COI	Insecta	Lepidoptera	Depressariidae				BOLD:ACE7937	Buchner & Stănescu 2019; ЛЕЛЕЙ et al. 2016			
COI	Insecta	Lepidoptera	Drepanidae	Thyatirinae	Tetheella	fluctuosa	BOLD:AAD3647				ЛЕЛЕЙ et al. 2016
COI	Insecta	Lepidoptera	Erebidae				BOLD:AAB7538	ЛЕЛЕЙ et al. 2016			
COI	Insecta	Lepidoptera	Erebidae	Erebinae	Catocala		BOLD:AAE9541			Sayama et al. 2012	
COI	Insecta	Lepidoptera	Erebidae	Arctiinae			BOLD:AAC3572		ЛЕЛЕЙ et al. 2016		
COI	Insecta	Lepidoptera	Erebidae	Hypeninae			BOLD:AAC7130		ЛЕЛЕЙ et al. 2016		
COI	Insecta	Lepidoptera	Erebidae	Erebinae	Ercheia		BOLD:AAD8285			Niwaka & Jimbo 2018; 13	
COI	Insecta	Lepidoptera	Erebidae	Erebinae	Thyas	juno	BOLD:AAF1405				ЛЕЛЕЙ et al. 2016
COI	Insecta	Lepidoptera	Erebidae	Erebinae			BOLD:ABX5513		ЛЕЛЕЙ et al. 2016		
COI	Insecta	Lepidoptera	Erebidae	Arctiinae			BOLD:ACE8889		ЛЕЛЕЙ et al. 2016		
COI	Insecta	Lepidoptera	Erebidae				BOLD:ACT9815	ЛЕЛЕЙ et al. 2016			
COI	Insecta	Lepidoptera	Erebidae				BOLD:ADF0678	ЛЕЛЕЙ et al. 2016			
COI	Insecta	Lepidoptera	Erebidae	Herminiinae	Hydrillodes		BOLD:ACU0270			ЛЕЛЕЙ et al. 2016	
COI	Insecta	Lepidoptera					BOLD:AAV8834				
COI	Insecta	Lepidoptera	Erebidae	Lymantriinae	Lymantria	dispar	BOLD:AAA2052				ЛЕЛЕЙ et al. 2016

Primer	Class	Order	Family	Subfamily	Genus	Species	BIN	Family	Subfamily	Genus	Species
COI	Insecta	Lepidoptera	Erebidae	Lymantriinae	Lymantria	monacha	BOLD:AAA5537				ЛЕЛЕЙ et al. 2016
COI	Insecta	Lepidoptera	Erebidae	Pangraptinae	Pangrapta		BOLD:ADF1765			ЛЕЛЕЙ et al. 2016	
COI	Insecta	Lepidoptera	Gelechiidae	Gelechiinae	Gelechia	cuneatella	BOLD:AAF5086				ЛЕЛЕЙ et al. 2016
COI	Insecta	Lepidoptera	Gelechiidae	Gelechiinae	Psoricoptera	gibbosella	BOLD:AAD0608				ЛЕЛЕЙ et al. 2016
COI	Insecta	Lepidoptera	Gelechiidae				BOLD:ABA1140	ЛЕЛЕЙ et al. 2016			
COI	Insecta	Lepidoptera	Geometridae	Ennominae			BOLD:ACG8719		Sayama et al. 2012; ЛЕЛЕЙ et al. 2016		
COI	Insecta	Lepidoptera	Geometridae	Ennominae	Cleora	insolita	BOLD:ACJ3813				ЛЕЛЕЙ et al. 2016
COI	Insecta	Lepidoptera	Geometridae	Ennominae			BOLD:AAC6655		Sayama et al. 2012		
COI	Insecta	Lepidoptera	Geometridae	Ennominae	Deileptenia	ribeata	BOLD:AAC3800				Sayama et al. 2012
COI	Insecta	Lepidoptera	Geometridae	Ennominae	Ectropis		BOLD:AAB5227			Sayama et al. 2012	
COI	Insecta	Lepidoptera	Geometridae	Ennominae	Ectropis	crepuscularia	BOLD:AAA2076				Sayama et al. 2012
COI	Insecta	Lepidoptera	Geometridae	Ennominae	Endropiodes		BOLD:AAF7479			ЛЕЛЕЙ et al. 2016	
COI	Insecta	Lepidoptera	Geometridae	Larentiinae	Gandaritis	fixseni	BOLD:AAY1335				Sayama et al. 2012
COI	Insecta	Lepidoptera	Geometridae	Larentiinae	Gandaritis		BOLD:AAB5983			Sayama et al. 2012	
COI	Insecta	Lepidoptera	Geometridae	Geometrinae	Geometra		BOLD:AAB2011			Sayama et al. 2012	
COI	Insecta	Lepidoptera	Geometridae	Ennominae	Protoboarmia		BOLD:AAA2077			Sayama et al. 2012; ЛЕЛЕЙ et al. 2016	
COI	Insecta	Lepidoptera	Geometridae	Larentiinae	Eupithecia		BOLD:AAA2083			Sayama et al. 2012	
COI	Insecta	Lepidoptera	Geometridae	Sterrhinae	Cyclophora		BOLD:AAD6021			ЛЕЛЕЙ et al. 2016	
COI	Insecta	Lepidoptera	Geometridae	Geometrinae			BOLD:AAE5087		ЛЕЛЕЙ et al. 2016		
COI	Insecta	Lepidoptera	Geometridae	Ennominae			BOLD:AAE6522		Sayama et al. 2012; ЛЕЛЕЙ et al. 2016		
COI	Insecta	Lepidoptera	Geometridae	Ennominae	Menophra	senilis	BOLD:AAF3823				Sayama et al. 2012
COI	Insecta	Lepidoptera	Geometridae	Ennominae			BOLD:AAL8521		Sayama et al. 2012; ЛЕЛЕЙ et al. 2016		
COI	Insecta	Lepidoptera	Geometridae	Ennominae			BOLD:AAP9222		Sayama et al. 2012; ЛЕЛЕЙ et al. 2016		
COI	Insecta	Lepidoptera	Geometridae				BOLD:AAZ1901	Yoshida 1991			
COI	Insecta	Lepidoptera	Geometridae	Ennominae			BOLD:ABU6285		Sayama et al. 2012; ЛЕЛЕЙ et al. 2016		
COI	Insecta	Lepidoptera	Geometridae	Geometrinae	Jodis	putata	BOLD:ABZ4040				ЛЕЛЕЙ et al. 2016
COI	Insecta	Lepidoptera	Geometridae	Larentiinae			BOLD:ACB9627		ЛЕЛЕЙ et al. 2016		
COI	Insecta	Lepidoptera	Geometridae				BOLD:ADF1852	Sayama et al. 2012, ЛЕЛЕЙ et al. 2016			
COI	Insecta	Lepidoptera	Geometridae	Sterrhinae	Scopula		LNAUS1341-13			Sayama et al. 2012	
COI	Insecta	Lepidoptera	Geometridae	Ennominae	Hypomecis	punctinalis	BOLD:ACA2461				Sayama et al. 2012
COI	Insecta	Lepidoptera	Geometridae	Ennominae			BOLD:ACS9064		Sayama et al. 2012; ЛЕЛЕЙ et al. 2016		
COI	Insecta	Lepidoptera	Geometridae	Ennominae			BOLD:AAB6560		ЛЕЛЕЙ et al. 2016		
COI	Insecta	Lepidoptera	Geometridae	Ennominae	Ourapteryx	maculicaudaria	BOLD:AAW9443				ЛЕЛЕЙ et al. 2016

Primer	Class	Order	Family	Subfamily	Genus	Species	BIN	Family	Subfamily	Genus	Species
COI	Insecta	Lepidoptera	Geometridae	Ennominae			BOLD:AAA2521		Sayama et al. 2012; ЛЕЛЕИ et al. 2016		
COI	Insecta	Lepidoptera	Geometridae	Ennominae	Phthonosema	tendinosaria	BOLD:AAF6489				ЛЕЛЕЙ et al. 2016
COI	Insecta	Lepidoptera	Gracillariidae	Gracillariinae	Caloptilia	cf. heringi	BOLD:ADK1669				ЛЕЛЕЙ et al. 2016
COI	Insecta	Lepidoptera	Gracillariidae	Gracillariinae	Caloptilia	hidakensis	BOLD:AAK1674				ЛЕЛЕЙ et al. 2016
COI	Insecta	Lepidoptera	Gracillariidae	Gracillariinae	Caloptilia		BOLD:ACL6329			ЛЕЛЕЙ et al. 2016	
COI	Insecta	Lepidoptera	Gracillariidae	Gracillariinae			BOLD:ADK2510		ЛЕЛЕЙ et al. 2016		
COI	Insecta	Lepidoptera	Hepialidae	Hepialinae			BOLD:AAO2948		Niwaka & Jimbo 2018; 14		
COI	Insecta	Lepidoptera	Hepialidae	Hepialinae			BOLD:ACN8703		Niwaka & Jimbo 2018; 15		
COI	Insecta	Lepidoptera	Hesperiidae	Coeliadinae	Burara/Bibasis	aquilina	BOLD:ACD6545				Tsukiji 2020; 16
COI	Insecta	Lepidoptera	Hesperiidae	Hesperiinae			LIMBC545-11	ЛЕЛЕЙ et al. 2016	ЛЕЛЕЙ et al. 2016		
COI	Insecta	Lepidoptera	Hesperiidae	Hesperiinae	Thoressa	varia	BOLD:ADK0840				Tsukiji 2020; 17
COI	Insecta	Lepidoptera	Lasiocampidae	Lasiocampinae	Euthrix	potatoria	BOLD:AAC1584				Sayama et al. 2012; Niwaka & Jimbo 2018; 18
COI	Insecta	Lepidoptera	Lecithoceridae				BOLD:AAD6680	Park et al. 2020; ЛЕЛЕЙ et al. 2016; Niwaka & Jimbo 2018; 19			
COI	Insecta	Lepidoptera	Limacodidae	Limacodinae			BOLD:ACE6221		Sayama et al. 2012		
COI	Insecta	Lepidoptera	Limacodidae	Limacodinae			BOLD:AAA9203		Sayama et al. 2012		
COI	Insecta	Lepidoptera	Limacodidae				BOLD:ACC2517	ЛЕЛЕЙ et al. 2016			
COI	Insecta	Lepidoptera	Limacodidae	Limacodinae	Parasa		BOLD:AAM0823			Sayama et al. 2012	
COI	Insecta	Lepidoptera	Limacodidae	Limacodinae	Phrixolepia		BOLD:AAV5028			ЛЕЛЕЙ et al. 2016; Niwaka & Jimbo 2018; 20	
COI	Insecta	Lepidoptera	Lycaenidae	Theclinae	Rapala		BOLD:AAL1375		ЛЕЛЕЙ et al. 2016	Korshunov & Gorbunov, 1995	
COI	Insecta	Lepidoptera	Noctuidae	Acronictinae	Acronicta		BOLD:ADK0044			Sayama et al. 2012; ЛЕЛЕЙ et al. 2016	
COI	Insecta	Lepidoptera	Noctuidae	Pantheinae	Anacronicta		BOLD:AAK1458			ЛЕЛЕЙ et al. 2016; Sayama et al. 2012	
COI	Insecta	Lepidoptera	Noctuidae	Noctuinae	Apamea	helva	BOLD:AAC5412				ЛЕЛЕЙ et al. 2016
COI	Insecta	Lepidoptera	Noctuidae	Noctuinae	Athetis		BOLD:AAV0425			ЛЕЛЕЙ et al. 2016	
COI	Insecta	Lepidoptera	Noctuidae	Acronictinae	Belciades	niveola	BOLD:ACU0724				Sayama et al. 2012; ЛЕЛЕЙ et al. 2016
COI	Insecta	Lepidoptera	Noctuidae	Amphipyrinae	Brachionycha	nubeculosa	BOLD:AAE0860				ЛЕЛЕЙ et al. 2016
COI	Insecta	Lepidoptera					BOLD:ADF8757				
COI	Insecta	Lepidoptera	Noctuidae				BOLD:ACT9151	Sayama et al. 2012; Niwaka & Jimbo 2018; 21			
COI	Insecta	Lepidoptera	Noctuidae	Noctuinae			BOLD:ACJ1078		Sayama et al. 2012; ЛЕЛЕИ et al. 2016		
COI	Insecta	Lepidoptera	Noctuidae	Oncocnemidinae			BOLD:AAE4319		ЛЕЛЕЙ et al. 2016		
COI	Insecta	Lepidoptera	Noctuidae	Plusiinae			BOLD:AAE7504		ЛЕЛЕЙ et al. 2016		
COI	Insecta	Lepidoptera	Noctuidae	Amphipyrinae			BOLD:AAW4866		ЛЕЛЕЙ et al. 2016		
COI	Insecta	Lepidoptera	Noctuidae	Noctuinae	Leucania		BOLD:AAJ2503			ЛЕЛЕЙ et al. 2016	
COI	Insecta	Lepidoptera	Noctuidae	Noctuinae	Lithophane	socia	BOLD:AAE6607				ЛЕЛЕЙ et al. 2016

Primer	Class	Order	Family	Subfamily	Genus	Species	BIN	Family	Subfamily	Genus	Species
COI	Insecta	Lepidoptera	Noctuidae	Noctuinae	Spodoptera	exigua	BOLD:AAA6644				ЛЕЛЕЙ et al. 2016
COI	Insecta	Lepidoptera	Noctuidae	Noctuinae	Spodoptera	cilium	BOLD:AAC8279			ЛЕЛЕЙ et al. 2016	Ratnasingham & Hebert 2007
COI	Insecta	Lepidoptera	Noctuidae	Amphipyrinae			BOLD:AAH5319		ЛЕЛЕЙ et al. 2016		
COI	Insecta	Lepidoptera					BOLD:ADG3545				
COI	Insecta	Lepidoptera					BOLD:AAB6211				
COI	Insecta	Lepidoptera	Noctuidae	Noctuinae			BOLD:AAB6980		Sayama et al. 2012; ЛЕЛЕЙ et al. 2016		
COI	Insecta	Lepidoptera	Noctuidae	Noctuinae	Xylena		BOLD:AAE4735			ЛЕЛЕЙ et al. 2016	
COI	Insecta	Lepidoptera	Nolidae	Chloephorinae	Nycteola		BOLD:AAE2641			ЛЕЛЕЙ et al. 2016	
COI	Insecta	Lepidoptera	Nolidae	Nolinae	Nola	confusalis	BOLD:AAB5563				Sayama et al. 2012
COI	Insecta	Lepidoptera	Nolidae	Nolinae			BOLD:AAL7275		Sayama et al. 2012		
COI	Insecta	Lepidoptera	Nolidae	Chloephorinae	Pseudoips	prasinana	BOLD:AAB8807				ЛЕЛЕЙ et al. 2016
COI	Insecta	Lepidoptera	Notodontidae				BOLD:AAM3801	ЛЕЛЕЙ et al. 2016			
COI	Insecta	Lepidoptera	Notodontidae	Notodontinae			BOLD:AAL6465		Sayama et al. 2012, ЛЕЛЕЙ et al. 2016		
COI	Insecta	Lepidoptera	Notodontidae	Heterocampinae			BOLD:AAM4825	ЛЕЛЕЙ et al. 2016	Ratnasingham & Hebert 2007		
COI	Insecta	Lepidoptera	Notodontidae	Notodontinae	Notodonta		BOLD:AAC1146			ЛЕЛЕЙ et al. 2016	
COI	Insecta	Lepidoptera	Notodontidae	Notodontinae	Shaka		BOLD:ACJ1076			Tsukiji 2020; 22	
COI	Insecta	Lepidoptera	Notodontidae	Heterocampinae	Stauropus	fagi	BOLD:AAD0646				Sayama et al. 2012
COI	Insecta	Lepidoptera	Nymphalidae	Heliconiinae	Boloria		BOLD:AAB9155			ЛЕЛЕЙ et al. 2016, Simonsen 2005	
COI	Insecta	Lepidoptera	Oecophoridae	Oecophorinae			BOLD:AAF9734		ЛЕЛЕЙ et al. 2016		
COI	Insecta	Lepidoptera	Papilionidae	Papilioninae	Papilio		BOLD:AAI5285			ЛЕЛЕЙ et al. 2016	
COI	Insecta	Lepidoptera	Pyralidae	Pyralinae	Endotricha	olivacealis	BOLD:ACI9403				ЛЕЛЕИ et al. 2016; Niwaka & Jimbo 2018; 23
COI	Insecta	Lepidoptera	Saturniidae				BOLD:AAA5428	Sayama et al. 2012; ЛЕЛЕЙ et al. 2016			
COI	Insecta	Lepidoptera	Saturniidae	Saturniinae	Saturnia		BOLD:AAB9581		Sayama et al. 2012	Sayama et al. 2012	
COI	Insecta	Lepidoptera	Saturniidae	Saturniinae	Saturnia	jonasii	BOLD:AAD1452				Sayama et al. 2012
COI	Insecta	Lepidoptera	Sphingidae	Smerinthinae	Callambulyx		BOLD:ABY4871			Sayama et al. 2012	
COI	Insecta	Lepidoptera	Sphingidae	Macroglossinae			BOLD:AAB0931		ЛЕЛЕИ et al. 2016; Niwaka & Jimbo 2018; 24		
COI	Insecta	Lepidoptera	Sphingidae	Sphinginae	Sphinx	ligustri	BOLD:AAB6107				ЛЕЛЕЙ et al. 2016
COI	Insecta	Lepidoptera	Sphingidae	Sphinginae	Sphinx		BOLD:ACF5246			ЛЕЛЕИ et al. 2016; Niwaka & Jimbo 2018; 25	
COI	Insecta	Lepidoptera	Stathmopodidae	NA	Stathmopoda	pedella	BOLD:AAD4282				ЛЕЛЕИ et al. 2016; Niwaka & Jimbo 2018; 26
COI	Insecta	Lepidoptera	Tineidae	Scardiinae	Morophaga	bucephala	BOLD:AAG8510				Sayama et al. 2012
COI	Insecta	Lepidoptera	Tortricidae	Tortricinae			BOLD:AAC3136		Sayama et al. 2012, ЛЕЛЕИ et al. 2016		
COI	Insecta	Lepidoptera	Tortricidae	Tortricinae	Archips	betulana	BOLD:ACM3437				ЛЕЛЕЙ et al. 2016
COI	Insecta	Lepidoptera	Tortricidae	Tortricinae	Archips		BOLD:AAB5839			Sayama et al. 2012, ЛЕЛЕИ et al. 2016	

Primer	Class	Order	Family	Subfamily	Genus	Species	BIN	Family	Subfamily	Genus	Species
COI	Insecta	Lepidoptera	Tortricidae	Tortricinae	Choristoneura	diversana	BOLD:AAD8048			ЛЕЛЕЙ et al. 2016	ЛЕЛЕИ et al. 2016; 北海道森林害虫図鑑 (affrc.go.jp)
COI	Insecta	Lepidoptera	Tortricidae	Tortricinae			BOLD:ACT2120		Sayama et al. 2012, ЛЕЛЕИ et al. 2016		
COI	Insecta	Lepidoptera	Tortricidae	Tortricinae	Dichelia		ANICU1645-11			ЛЕЛЕЙ et al. 2016	
COI	Insecta	Lepidoptera	Tortricidae	Tortricinae	Eana	incanana	BOLD:AAD7476				Niwaka & Jimbo 2018; 27
COI	Insecta	Lepidoptera	Tortricidae	Olethreutinae	Epinotia		BOLD:AAE1784			Sayama et al. 2012	
COI	Insecta	Lepidoptera	Tortricidae	Olethreutinae	Epinotia	nisella	BOLD:AAA7530			Sayama et al. 2012	ЛЕЛЕЙ et al. 2016
COI	Insecta	Lepidoptera	Tortricidae	Olethreutinae	Eudemis	porphyrana	BOLD:AAC6854				ЛЕЛЕЙ et al. 2016
COI	Insecta	Lepidoptera	Tortricidae	Olethreutinae	Gypsonoma	dealbana	BOLD:AAB0380				Sayama et al. 2012
COI	Insecta	Lepidoptera	Tortricidae	Olethreutinae	Lobesia		BOLD:ABV8007			ЛЕЛЕЙ et al. 2016	
COI	Insecta	Lepidoptera	Tortricidae	Olethreutinae			BOLD:ABZ7645		Sayama et al. 2012		
COI	Insecta	Lepidoptera	Tortricidae	Olethreutinae	Olethreutes		BOLD:ACS0054			Niwaka & Jimbo 2018; 28	
COI	Insecta	Lepidoptera	Tortricidae	Tortricinae	Pandemis	cinnamomeana	BOLD:AAD0575				ЛЕЛЕЙ et al. 2016
COI	Insecta	Lepidoptera	Tortricidae	Tortricinae	Pandemis	corylana	BOLD:AAC5400				ЛЕЛЕЙ et al. 2016
COI	Insecta	Lepidoptera	Tortricidae	Olethreutinae	Phiaris		BOLD:AAJ2026			ЛЕЛЕЙ et al. 2016	
COI	Insecta	Lepidoptera	Tortricidae	Tortricinae	Ptycholoma	lecheanum	BOLD:AAD3264				Tsukiji 2020; 29
COI	Insecta	Lepidoptera	Tortricidae	Olethreutinae	Rhopobota	naevana	BOLD:AAA9812				Sayama et al. 2012
COI	Insecta	Lepidoptera	Tortricidae	Olethreutinae			BOLD:AAA0213		Niwaka & Jimbo 2018; 30		
COI	Insecta	Lepidoptera	Tortricidae	Olethreutinae	Spilonota	laricana	BOLD:AAA7739				ЛЕЛЕЙ et al. 2016
COI	Insecta	Lepidoptera	Tortricidae	Tortricinae	Archips	crataeganus	BOLD:AAD6620			Sayama et al. 2012, ЛЕЛЕЙ et al. 2016	Bong-kyu et al. 2003
COI	Insecta	Lepidoptera					BOLD:AAH4639				
COI	Insecta	Lepidoptera	Tortricidae	Olethreutinae	Epinotia		BOLD:AAN7425			Sayama et al. 2012	
COI	Insecta	Lepidoptera	Tortricidae	Olethreutinae			BOLD:AAV9504		Sayama et al. 2012		
COI	Insecta	Lepidoptera	Tortricidae				BOLD:ADE9961	Sayama et al. 2012, ЛЕЛЕЙ et al. 2016			
COI	Insecta	Lepidoptera	Tortricidae	Tortricinae			BOLD:ACP8754		Sayama et al. 2012, ЛЕЛЕЙ et al. 2016		
COI	Insecta	Lepidoptera	Tortricidae	Olethreutinae	Zeiraphera	rufimitrana	BOLD:AAM3356				ЛЕЛЕЙ et al. 2016
COI	Insecta	Lepidoptera	Uraniidae	Uraniinae			BOLD:ACF4699		ЛЕЛЕЙ et al. 2016		
COI	Insecta	Lepidoptera	Yponomeutidae	Yponomeutinae			BOLD:AAE4109		ЛЕЛЕЙ et al. 2016; Na et al. 2018		
COI	Insecta	Lepidoptera	Ypsolophidae	Ypsolophinae	Ypsolopha	vittella	BOLD:AAD9548				ЛЕЛЕЙ et al. 2016
COI	Insecta	Lepidoptera					BOLD:AAD3218				
COI	Insecta	Mecoptera	Panorpidae	Panorpinae	Panorpa	pryeri	BOLD:ACA3726				中田 et al. 2006
COI	Insecta	Neuroptera	Hemerobiidae	Drepanepteryginae	Drepanepteryx		BOLD:ACT2374			Dobosz et al. 2019	
COI	Insecta	Neuroptera	Hemerobiidae	Hemerobiinae	Hemerobius		BOLD:AAG0897			Tsukiji 2020; 31	
COI	Insecta	Neuroptera	Hemerobiidae	Hemerobiinae	Hemerobius		BOLD:ABZ0149			Tsukiji 2020; 32	
COI	Insecta	Neuroptera	Hemerobiidae	Hemerobiinae	Hemerobius	fenestratus	BOLD:AAU3559				Dobosz et al. 2019

Primer	Class	Order	Family	Subfamily	Genus	Species	BIN	Family	Subfamily	Genus	Species
COI	Insecta	Neuroptera	Hemerobiidae	Hemerobiinae	Hemerobius	simulans	BOLD:ACM1832				Dobosz et al. 2019
COI	Insecta	Neuroptera	Hemerobiidae	Hemerobiinae			BOLD:ABU9030		Dobosz et al. 2019		
COI	Insecta	Neuroptera	Osmylidae				INRMA502-12	Dobosz et al. 2019			
COI	Insecta	Plecoptera	Nemouridae	Amphinemurinae			BOLD:AAL6222		DeWalt et al. 2020; 33		
COI	Insecta	Psocodea	Psocidae	Psocinae	Psococerastis		BOLD:ACC5474			Tsukiji 2020; 34	
COI	Insecta	Trichoptera	Lepidostomatidae	Lepidostomatinae	Lepidostoma	elongatum	RUSST089-12			Yang & Weaver 2002	Yang & Weaver 2002
COI	Insecta	Trichoptera	Psychomyiidae	Psychomyiinae	Tinodes	higashiyamanus	BOLD:ACD6706				Ito 2000, 2017
COI	Insecta	Trichoptera	Thremmatidae	Thremmatinae	Neophylax	ussuriensis	BOLD:AAG9568				Nozaki 2020
16S	Arachnida	Opiliones	Sclerosomatidae	Leiobuninae	Leiobunum	tohokuense	NA				Burns et al. 2018
16S	Insecta	Coleoptera	Elateridae	Elaterinae	Agriotes		NA		Oba et al. 2015	Oba et al. 2015	
16S	Insecta	Coleoptera	Scarabaeidae	Rutelinae	Anomala		NA			Kobayashi & Matsumoto 2011	
16S	Insecta	Coleoptera	Curculionidae	Curculioninae	Curculio		NA			Notsu 1994	
16S	Insecta	Coleoptera	Cerambycidae	Lamiinae	Eutetrapha		NA			Lin et al. 2017	
16S	Insecta	Coleoptera	Tenebrionidae	Alleculinae	Hymenalia		NA		Akita & Matsumoto 2012	Isono et al. 1986	
16S	Insecta	Coleoptera	Scarabaeidae				NA	Kobayashi & Matsumoto 2011			
16S	Insecta	Coleoptera	Scarabaeidae				NA	Kobayashi & Matsumoto 2011			
16S	Insecta	Coleoptera	Melandryidae	Malandryinae	Phloiotrya	planiuscula	NA			Ishihama & Hara 2006	Löbl & Smetana 2008
16S	Insecta	Coleoptera	Scarabaeidae				NA	Kobayashi & Matsumoto 2011			
16S	Insecta	Coleoptera	Cantharidae	Cantharinae	Podabrus		NA			Kazantsev 2001	
16S	Insecta	Coleoptera	Chrysomelidae				NA	Cho & Świętojańska 2017			
16S	Insecta	Dermaptera	Forficulidae	Anechurinae	Anechura	harmandi	NA				Nishikawa 2009
16S	Insecta	Dermaptera	Forficulidae	Forficulinae	Forficula	mikado	NA				Nishikawa 2009
16S	Insecta	Diptera	Mycetophilidae	Gnoristinae	Boletina		NA			Ratnasingham & Hebert 2007	
16S	Insecta	Diptera	Tachinidae	Exoristinae	Botria	japonica	NA				O'Hara et al. 2019
16S	Insecta	Diptera	Sciaridae	NA	Chaetosciara		NA	Menzel & Mohrig 2000	NA	Menzel 1999	
16S	Insecta	Diptera	Calliphoridae	Chrysomyinae	Chrysomya		NA		Iwasa & Hori 1990	Erzinclioglu 1990	
16S	Insecta	Diptera	Empidoidea				NA	Maeda 2011			
16S	Insecta	Diptera	Culicidae	Culicinae	Culex		NA			Fonseca et al. 2009	
16S	Insecta	Diptera	Culicidae	Culicinae	Culex	tritaeniorhynchus	NA				Takashima et al. 1989
16S	Insecta	Diptera	Limoniidae	Limoniinae	Dicranomyia		NA			Savchenko et al. 1992	
16S	Insecta	Diptera	Limoniidae	Limoniinae	Dicranomyia		NA			Savchenko et al. 1992	
16S	Insecta	Diptera	Limoniidae	Limoniinae	Dicranomyia		NA			Savchenko et al. 1992	
16S	Insecta	Diptera	Limoniidae	Limoniinae	Dicranomyia		NA			Savchenko et al. 1992	
16S	Insecta	Diptera	Tachinidae	Exoristinae	Eumea	linearicornis	NA				O'Hara et al. 2019
16S	Insecta	Diptera	Mycetophilidae	Mycetophilinae	Exechia		NA			Ratnasingham & Hebert 2007	
16S	Insecta	Diptera	Mycetophilidae	Mycetophilinae	Exechia		NA			Ratnasingham & Hebert 2007	

Primer	Class	Order	Family	Subfamily	Genus	Species	BIN	Family	Subfamily	Genus	Species
16S	Insecta	Diptera	Tachinidae	Exoristinae	Gonia	chinensis	NA				O'Hara et al. 2019
16S	Insecta	Diptera	Limoniidae				NA	Savchenko et al. 1992			
16S	Insecta	Diptera	Chironomidae	Chironominae	Micropsectra		NA			Ratnasingham & Hebert 2007	
16S	Insecta	Diptera	Chironomidae	Chironominae	Micropsectra		NA			Ratnasingham & Hebert 2007	
16S	Insecta	Diptera	Mycetophilidae	Mycetophilinae	Mycetophila		NA		Ratnasingham & Hebert 2007	Matsumoto 2006	
16S	Insecta	Diptera	Mycetophilidae	Mycetophilinae	Mycetophila	fungorum	NA				Okada 1939
16S	Insecta	Diptera	Mycetophilidae	Mycomyinae	Mycomya		NA	Ratnasingham & Hebert 2007	笹川 2003	笹川 2003	
16S	Insecta	Diptera					NA				
16S	Insecta	Diptera	Empidoidea				NA	Maeda 2011			
16S	Insecta	Diptera	Limoniidae				NA	Savchenko et al. 1992			
16S	Insecta	Diptera	Psychodidae				NA	Sanjoba et al. 2011			
16S	Insecta	Diptera	Rhagionidae				NA	Imada & Kato 2016			
16S	Insecta	Diptera	Tipulidae				NA	Men et al. 2019			
16S	Insecta	Diptera	Culicidae	Culicinae	Ochlerotatus		NA			Ratnasingham & Hebert 2007	
16S	Insecta	Diptera	Pediciidae	Pediciinae	Pedicia		NA			Savchenko et al. 1992	
16S	Insecta	Diptera	Rhinophoridae				NA	Kato & Tachi 2016			
16S	Insecta	Diptera	Mycetophilidae	Mycetophilinae	Pseudobrachypeza		NA			Ratnasingham & Hebert 2007	
16S	Insecta	Diptera	Phoridae	Phorinae	Stichillus		NA			Nakayama & Shima 2004	
16S	Insecta	Diptera	Tachinidae	Exoristinae	Suensonomyia	nudinerva	NA				O'Hara et al. 2019
16S	Insecta	Diptera	Mycetophilidae				NA	Ratnasingham & Hebert 2007			
16S	Insecta	Diptera	Muscidae	Azeliinae	Thricops		NA			Beppu 2004	
16S	Insecta	Diptera	Tipulidae	Tipulinae	Tipula		NA			Men et al. 2019	
16S	Insecta	Diptera	Tipulidae	Tipulinae	Tipula		NA			Men et al. 2019	
16S	Insecta	Diptera	Mycetophilidae	Mycetophilinae			NA		Ratnasingham & Hebert 2007		
16S	Insecta	Ephemeroptera	Heptageniidae	Rhithrogeniinae	Rhithrogena		NA			Ishiwata 2001	
16S	Insecta	Ephemeroptera	Heptageniidae	Rhithrogeniinae	Rhithrogena		NA			Ishiwata 2001	
16S	Insecta	Hemiptera	Miridae	Mirinae	Adelphocoris		NA			Zhang et al. 2015	
16S	Insecta	Hemiptera	Miridae	Deraeocorinae	Alloeotomus	simplus	NA	Zhang et al. 2015	Poorani et al. 2019	Seong & Lee 2007	Seong & Lee 2007
16S	Insecta	Hemiptera	Miridae	Mirinae	Arbolygus	rubripes	NA				Tsukiji 2020; 35
16S	Insecta	Hemiptera	Acanthosomatidae	Acanthosomatinae	Elasmucha	signoreti	NA				Tsukiji 2020; 36
16S	Insecta	Hemiptera	Miridae	Mirinae	Phytocoris		NA			Yasunaga & Schwartz 2015	
16S	Insecta	Hemiptera	Psyllidae	Psyllinae	Psylla		NA			Cho et al. 2019	
16S	Insecta	Hemiptera	Psyllidae	Psyllinae	Psylla		NA			Cho et al. 2019	
16S	Insecta	Hemiptera	Aphididae	Calaphidinae	Symydobius	kabae	NA		Simbaqueba et al. 2016	Tsukiji 2020; 37	Qiao & Zhang 2002
16S	Insecta	Hymenoptera	Braconidae	Rogadinae	Aleiodes		NA	Ratnasingham & Hebert 2007	Butcher et al. 2016	Samin et al. 2016	
16S	Insecta	Hymenoptera	Ichneumonidae	Campopleginae	Hyposoter		NA		Watanabe 2019	Watanabe 2015	
16S	Insecta	Hymenoptera	Braconidae	Microgastrinae	Microplitis		NA			Kaneko 1993	
16S	Insecta	Hymenoptera					NA				

Primer	Class	Order	Family	Subfamily	Genus	Species	BIN	Family	Subfamily	Genus	Species
16S	Insecta	Hymenoptera	Formicidae				NA	寺山 et al. 2020			
16S	Insecta	Hymenoptera	Tenthredinidae	Nematinae	Nematinus	luteus	NA				Togashi 2007
16S	Insecta	Lepidoptera	Geometridae	Ennominae	Arichanna	melanaria	NA				ЛЕЛЕЙ et al. 2016
16S	Insecta	Lepidoptera	Noctuidae				NA	ЛЕЛЕИ et al. 2016, Sayama et al. 2012			
16S	Insecta	Lepidoptera	Tortricidae	Tortricinae			NA		Sayama et al. 2012, ЛЕЛЕЙ et al. 2016		
16S	Insecta	Trichoptera	Limnephilidae	Dicosmoecinae	Dicosmoecus		NA			Ratnasingham & Hebert 2007	
16S	Insecta	Lepidoptera	Geometridae	Ennominae	Ectropis	obliqua	NA				ЛЕЛЕЙ et al. 2016
16S	Insecta	Lepidoptera	Tortricidae	Tortricinae			NA		Sayama et al. 2012, ЛЕЛЕЙ et al. 2016		
16S	Insecta	Lepidoptera	Geometridae	Ennominae	Jankowskia		NA			ЛЕЛЕЙ et al. 2016	
16S	Insecta	Lepidoptera	Geometridae	Larentiinae	Lobogonodes	erectaria	NA				Sayama et al. 2012
16S	Insecta	Lepidoptera	Erebidae	Lymantriinae	Lymantria		NA			ЛЕЛЕЙ et al. 2016	
16S	Insecta	Lepidoptera	Geometridae	Ennominae	Myrioblephara	cilicornaria	NA				Niwaka & Jimbo 2018; 38
16S	Insecta	Lepidoptera					NA				
16S	Insecta	Lepidoptera	Noctuidae	Noctuinae	Panolis		NA			ЛЕЛЕЙ et al. 2016	
16S	Insecta	Lepidoptera	Noctuidae	Hadeninae	Panolis		NA			ЛЕЛЕЙ et al. 2016	
16S	Insecta	Lepidoptera	Noctuidae	Noctuinae	Panolis		NA			ЛЕЛЕЙ et al. 2016	
16S	Insecta	Lepidoptera	Noctuidae				NA	ЛЕЛЕЙ et al. 2016, Sayama et al. 2012			
16S	Insecta	Lepidoptera	Noctuidae				NA	ЛЕЛЕЙ et al. 2016, Sayama et al. 2012			
16S	Insecta	Lepidoptera	Thyrididae	Siculodinae	Pyrinioides	aurea	NA				Niwaka & Jimbo 2018; 39
16S	Insecta	Lepidoptera	Saturniidae	Saturniinae	Saturnia		NA			Sayama et al. 2012	
16S	Insecta	Mecoptera	Panorpidae	Panorpinae	Panorpa	takenouchii	NA			中田 et al. 2006	Penny & Byers 1979
16S	Insecta	Neuroptera	Chrysopidae	Chrysopinae	Apertochrysa		NA			Tsukaguchi & Tago 2018	
16S	Insecta	Neuroptera	Chrysopidae	Chrysopinae	Chrysoperla		NA			Tsukiji 2020; 40	
16S	Insecta	Neuroptera	Chrysopidae	Chrysopinae	Chrysoperla		NA			Tsukiji 2020; 41	
16S	Insecta	Neuroptera	Osmylidae	Osmylinae	Osmylus		NA			Tsukiji 2020; 42	
16S	Insecta	Neuroptera	Hemerobiidae	Hemerobiinae			NA		Dobosz et al. 2019		
16S	Insecta	Odonata	Libellulidae	Sympetrinae	Sympetrum	frequens	NA				Tsukiji 2020; 43
16S	Insecta	Odonata	Libellulidae	Sympetrinae	Sympetrum	infuscatum	NA				Tsukiji 2020; 44
16S	Insecta	Orthoptera	Rhaphidophoridae	Aemodogryllinae			NA		Tsukiji 2020; 45		
16S	Insecta	Orthoptera	Trigonidiidae	Nemobiinae	Dianemobius	nigrofasciatus	NA				Tsukiji 2020; 46
16S	Insecta	Orthoptera	Rhaphidophoridae	Aemodogryllinae	Diestrammena		NA			Tsukiji 2020; 47	
16S	Insecta	Orthoptera	Rhaphidophoridae				NA	Tsukiji 2020; 48			
16S	Insecta	Orthoptera	Rhaphidophoridae				NA	Tsukiji 2020; 49			
16S	Insecta	Plecoptera	Perlidae	Acroneuriinae	Calineuria		NA			Uchida 1983; DeWalt et al. 2020; 50	
16S	Insecta	Plecoptera	Nemouridae				NA	DeWalt et al. 2020; 51			

Primer	Class	Order	Family	Subfamily	Genus	Species	BIN	Family	Subfamily	Genus	Species
16S	Insecta	Psocodea	Amphipsocidae	Amphipsocinae	Amphipsocus	japonicus	NA				Yoshizawa 2001
16S	Insecta	Psocodea					NA				
16S	Insecta	Psocodea	Mesopsocidae	NA	Mesopsocus	unipunctatus	NA				Yoshizawa 1998
16S	Insecta	Psocodea	Psocidae	Psocinae	Psococerastis		NA			Tsukiji 2020; 52	
16S	Insecta	Psocodea	Psocidae	Psocinae	Psococerastis	nubila	NA				Yoshizawa 2002
16S	Insecta	Psocodea	Stenopsocidae	NA	Stenopsocus	nigricellus	NA			Tsukiji 2020; 53	Yoshizawa 2002
16S	Insecta	Psocodea	Psocidae	Psocinae	Trichadenotecnum		NA			Yoshizawa 2004	
16S	Insecta	Psocodea	Psocidae	Psocinae	Trichadenotecnum	incognitum	NA			Yoshizawa 2004	Yoshizawa 2004
16S	Insecta	Psocodea	Psocidae	Psocinae	Trichadenotecnum		NA			Yoshizawa 2004	
16S	Insecta	Thysanoptera	Thripidae	Thripinae	Thrips		NA			Iwasaki et al. 2005	

Hompage links		Accessed
1	http://cockroach.speciesfile.org/Common/basic/Taxa.aspx?TaxonNameID=1175933	23-06-20
2	http://www.catalogueoflife.org/col/details/species/id/4fa0e6dd77a4885e51e61d52aeecedac	
3	http://www.jpmoth.org/Coleophoridae/Coleophora_albicans.html	27-04-20
4	http://www.jpmoth.org/Crambidae/Scopariinae/Eudonia_persimilis.html	27-04-20
5	http://www.jpmoth.org/Crambidae/Pyraustinae/Pleuroptya_expictalis.html	27-04-20
6	http://www.jpmoth.org/Elachistidae/Perittia_andoi.html	27-04-20
7	http://www.jpmoth.org/Lasiocampidae/Poecilocampinae/Poecilocampa_tamanukii.html	27-04-20
8	http://www.jpmoth.org/Stathmopodidae/Stathmopoda_pedella.html	27-04-20
9	http://www.jpmoth.org/Blastobasidae/Hypatopa_montivaga.html	27-04-20
10	http://www.jpmoth.org/Coleophoridae/Coleophora_albicans.html	27-04-20
11	http://www.jpmoth.org/Crambidae/Pyraustinae/Pyrausta_aurata.html	27-04-20
12	http://www.jpmoth.org/Crambidae/Pyraustinae/Pleuroptya_ruralis.html	27-04-20
13	http://www.jpmoth.org/Noctuidae/Catocalinae/Ercheia_umbrosa.html	27-04-20
14 & 15	http://www.jpmoth.org/Hepialidae/index.html	27-04-20
16 & 17	https://mushinavi.com/	12-05-20
18	Euthrix potatoria bergmanihttp://www.jpmoth.org/Lasiocampidae/Lasiocampinae/Euthrix.html	27-04-20
19	http://www.jpmoth.org/Lecithoceridae/Oditinae/Scythropiodes_issikii.html	27-04-20
20	http://www.jpmoth.org/Limacodidae/Limacodinae/Phrixolepia_sericea.html	27-04-20
21	http://www.jpmoth.org/Noctuidae/Noctuinae/Diarsia_canescens.html	27-04-20
22	https://mushinavi.com/	15-05-20
23	http://www.jpmoth.org/Pyralidae/Pyralinae/Endotricha_olivacealis.html	27-04-20
24	http://www.jpmoth.org/Sphingidae/Macroglossinae/Hemaris_affinis.html	27-04-20
25	Sphinx morio inoueihttp://www.jpmoth.org/Sphingidae/Sphinginae/Sphinx_morio_inouei.html	27-04-20
26	http://www.jpmoth.org/Stathmopodidae/Stathmopoda_pedella.html	27-04-20
27	http://www.jpmoth.org/Tortricidae/Tortricinae/Eana_incanana.html	27-04-20
28	http://www.jpmoth.org/Tortricidae/Olethreutinae/Olethreutes_bipunctanus_yama.html	15-05-20

	Hompage links	Accessed
29	https://mushinavi.com/	12-05-20
30	http://www.jpmoth.org/Tortricidae/Olethreutinae/Bactra_furfurana.html	27-04-20
31 & 32	https://mushinavi.com/	08-05-20
33	http://plecoptera.speciesfile.org	08-05-20
34	https://mushinavi.com/	08-05-20
35-37	https://mushinavi.com/	07-05-20
38	http://www.jpmoth.org/Geometridae/Ennominae/Myrioblephara_cilicornaria.html	27-04-20
39	http://www.jpmoth.org/Thyrididae/Siculodinae/Pyrinioides_aurea.html	27-04-20
40-49	https://mushinavi.com/	08-05-20
50	http://plecoptera.speciesfile.org/Common/editTaxon/Distribution/ShowDistribution.aspx?TaxonNameID=1157210	08-05-20
51	http://plecoptera.speciesfile.org	08-05-20
52 & 53	https://mushinavi.com/	08-05-20
	https://www.boldsystems.org/ (cited above as Ratnasingham & Hebert 2007)	27-04-20 - 07-05-2020
