# Supplementary material for: Dietary analysis reveals differences in the prey use of two sympatric bat species
Source: Ecol Evol. 2021 Dec 16;11(24):18651–61. doi: 10.1002/ece3.8472 (PMC8717349; doi:10.1002/ece3.8472)
Supplement: Supplementary file 2 — Appendix S2 [file ECE3-11-18651-s005.pdf]

Supplementary information S2 for *Dietary analysis reveals differences in the prey use of two sympatric bat species*

Olga Heim[1], Anna I.E. Puisto[2], Ilari Sääksjärvi[3], Dai Fukui[4] and Eero J. Vesterinen[5]*

1 Faculty of Life and Medical Sciences, Doshisha University, 610-0321 Kyotanabe, Japan

2 Centre for Population Health Research, University of Turku, 20014 Turku, Finland

3 Biodiversity Unit, University of Turku, 20014 Turku, Finland

4 The University of Tokyo Hokkaido Forest, The University of Tokyo, 079-1563 Furano, Japan

5 Department of Biology, University of Turku, 20014 Turku, Finland

Percentage of occurrence data

16S

Murina ussuriensis	Myotis ikonnikovi	
54	76	Count of occurrences
88	140	Sum of occurrences
43	39	Number of samples

COI

Murina ussuriensis	Myotis ikonnikovi	
180	264	Count of occurrences
234	474	Sum of occurrences
49	45	Number of samples

Fusion

Murina ussuriensis	Myotis ikonnikovi	
234	340	Count of occurrences
322	614	Sum of occurrences
53	45	Number of samples

								Percentage of occurrence		Number of samples where prey item occurred	
**Primer**	**Class**	**Order**	**Family**	**Subfamily**	**Genus**	**Species**	**BIN**	**M. ussuriensis**	**M. ikonnikovi**	**M. ussuriensis**	**M. ikonnikovi**
COI	Arachnida	Araneae	Lycosidae	NA	Pardosa		BOLD:ABA5238	0.009	0	3	0
COI	Arachnida	Araneae	Salticidae				BOLD:AAV1597	0.003	0	1	0
COI	Arachnida	Araneae	Salticidae				GBCH10991-13	0.003	0	1	0
COI	Arachnida	Araneae	Salticidae				BOLD:ACH5240	0.003	0	1	0
COI	Collembola	Symphypleona	Bourletiellidae	NA	Deuterosminthurus		BOLD:AAB7915	0.003	0	1	0
COI	Insecta	Blattodea	Ectobiidae	Ectobiinae			BOLD:ACI8100	0.003	0	1	0
COI	Insecta	Coleoptera	Carabidae				BOLD:AAZ4621	0.006	0	2	0
COI	Insecta	Coleoptera	Coccinellidae				BOLD:ACP4109	0	0.0016	0	1
COI	Insecta	Coleoptera	Cantharidae	Cantharinae	Podabrus		BOLD:ACD6222	0	0.0049	0	3
COI	Insecta	Coleoptera	Cantharidae	Cantharinae	Podabrus		BOLD:ACD6257	0	0.0016	0	1
COI	Insecta	Coleoptera	Carabidae	Harpalinae			BOLD:ABX4019	0.003	0.0016	1	1
COI	Insecta	Coleoptera	Carabidae	Carabinae	Carabus		BOLD:ABY6003	0	0.0016	0	1
COI	Insecta	Coleoptera	Carabidae	Harpalinae	Pterostichus	oblongopunctatus	BOLD:ABY4764	0.003	0	1	0
COI	Insecta	Coleoptera	Cerambycidae	Lamiinae	Monochamus		BOLD:AAC3388	0.003	0	1	0
COI	Insecta	Coleoptera	Chrysomelidae	Chrysomelinae			BOLD:AAC0432	0.006	0.0016	2	1
COI	Insecta	Coleoptera	Cryptophagidae	Atomariinae	Atomaria	turgida	BOLD:AAJ9463	0	0.0016	0	1
COI	Insecta	Coleoptera	Curculionidae	Entiminae	Polydrusus		BOLD:ACO8630	0	0.0016	0	1
COI	Insecta	Coleoptera	Hydrophilidae	Hydrophilinae	Enochrus		BOLD:AAF0204	0.003	0	1	0

Primer	Class	Order	Family	Subfamily	Genus	Species	BIN	M. ussuriensis	M. ikonnikovi	M. ussuriensis	M. ikonnikovi
COI	Insecta	Coleoptera	Nitidulidae	Nitidulinae	Cychramus	variegatus	BOLD:ABW4745	0	0.0033	0	2
COI	Insecta	Coleoptera	Staphylinidae	Euaesthetinae	Euaesthetus		BOLD:ACG9244	0	0.0016	0	1
COI	Insecta	Diptera	Anthomyiidae				BOLD:AAG2490	0	0.0016	0	1
COI	Insecta	Diptera	Tachinidae	Exoristinae			BOLD:AAI6325	0.003	0	1	0
COI	Insecta	Diptera	Tipulidae	Tipulinae	Tipula		BOLD:AAN5045	0	0.0016	0	1
COI	Insecta	Diptera	Tipulidae	Tipulinae	Tipula		BOLD:AAN9029	0.003	0.0033	1	2
COI	Insecta	Diptera	Limoniidae	Chioneinae	Teucholabis		BOLD:AAV2508	0	0.0016	0	1
COI	Insecta	Diptera	Limoniidae	Chioneinae			BOLD:ABA4102	0	0.0016	0	1
COI	Insecta	Diptera	Cecidomyiidae				BOLD:ABV2094	0.003	0	1	0
COI	Insecta	Diptera	Limoniidae				BOLD:ABX4369	0	0.0016	0	1
COI	Insecta	Diptera	Limoniidae	Limoniinae	Libnotes		BOLD:ABY2385	0	0.0033	0	2
COI	Insecta	Diptera	Syrphidae	Eristalinae			BOLD:ABY2389	0	0.0016	0	1
COI	Insecta	Diptera	Tipulidae	Tipulinae	Tipula		BOLD:ABY5691	0.003	0	1	0
COI	Insecta	Diptera	Phoridae				BOLD:ACA3150	0.003	0	1	0
COI	Insecta	Diptera	Tachinidae	Tachininae	Nemoraea	takanoi	BOLD:ACA7375	0.003	0	1	0
COI	Insecta	Diptera	Chironomidae	Chironominae	Glyptotendipes		BOLD:ACE3296	0	0.0016	0	1
COI	Insecta	Diptera	Tachinidae				BOLD:ACF0578	0.003	0	1	0
COI	Insecta	Diptera	Tipulidae	Tipulinae	Tipula		BOLD:ACF0878	0	0.0016	0	1
COI	Insecta	Diptera	Phoridae				BOLD:ACF6080	0.012	0.0049	4	3
COI	Insecta	Diptera	Sphaeroceridae	Limosininae	Rachispoda		BOLD:ACK2192	0	0.0016	0	1
COI	Insecta	Diptera	Chironomidae				BOLD:ACK8680	0	0.0016	0	1
COI	Insecta	Diptera	Sciaridae				BOLD:ACM6077	0	0.0016	0	1
COI	Insecta	Diptera	Simuliidae				BOLD:ACM8222	0	0.0016	0	1
COI	Insecta	Diptera	Limoniidae				BOLD:ACN0178	0	0.0016	0	1
COI	Insecta	Diptera	Limoniidae				BOLD:ACN0238	0	0.0016	0	1
COI	Insecta	Diptera	Mycetophilidae				BOLD:ACN0859	0	0.0049	0	3
COI	Insecta	Diptera	Calliphoridae				BOLD:ACN1515	0.003	0	1	0
COI	Insecta	Diptera	Psychodidae				BOLD:ACN3276	0	0.0016	0	1
COI	Insecta	Diptera	Drosophilidae				BOLD:ACN4805	0	0.0016	0	1
COI	Insecta	Diptera					BOLD:ACN5240	0	0.0016	0	1
COI	Insecta	Diptera	Limoniidae				BOLD:ACN5325	0	0.0033	0	2
COI	Insecta	Diptera	Phoridae				BOLD:ACO0272	0.006	0	2	0
COI	Insecta	Diptera	Chironomidae				BOLD:ACO4488	0	0.0016	0	1
COI	Insecta	Diptera					BOLD:ACP6542	0	0.0033	0	2
COI	Insecta	Diptera	Chloropidae				BOLD:ACR0489	0	0.0016	0	1
COI	Insecta	Diptera	Muscidae				BOLD:ACT7328	0	0.0016	0	1
COI	Insecta	Diptera	Sciaridae				BOLD:ACU8386	0	0.0016	0	1
COI	Insecta	Diptera	Muscidae	Phaoniinae	Helina		BOLD:ACV2533	0	0.0016	0	1

Primer	Class	Order	Family	Subfamily	Genus	Species	BIN	M. ussuriensis	M. ikonnikovi	M. ussuriensis	M. ikonnikovi
COI	Insecta	Diptera	Psychodidae				BOLD:ACW2931	0	0.0016	0	1
COI	Insecta	Diptera	Agromyzidae				BOLD:AAV4874	0	0.0016	0	1
COI	Insecta	Diptera	Anthomyiidae	Anthomyiinae	Botanophila		BOLD:AAG2502	0	0.0016	0	1
COI	Insecta	Diptera	Anthomyiidae	Anthomyiinae	Lasiomma		BOLD:ACK1581	0.003	0	1	0
COI	Insecta	Diptera	Anthomyiidae	Anthomyiinae	Delia	platura	BOLD:AAG2511	0.003	0	1	0
COI	Insecta	Diptera	Anthomyiidae	Anthomyiinae	Botanophila	profuga	BOLD:ACP6334	0.003	0.0033	1	2
COI	Insecta	Diptera	Bolitophilidae	NA	Bolitophila		BOLD:AAM9007	0	0.0016	0	1
COI	Insecta	Diptera	Cecidomyiidae				BOLD:ACP9491	0.006	0	2	0
COI	Insecta	Diptera	Cecidomyiidae				BOLD:ACU9836	0	0.0033	0	2
COI	Insecta	Diptera	Cecidomyiidae	Cecidomyiinae	Feltiella	acarisuga	BOLD:ACD4136	0.003	0	1	0
COI	Insecta	Diptera	Chamaemyiidae				BOLD:ACC5885	0	0.0016	0	1
COI	Insecta	Diptera	Chironomidae	Diamesinae	Diamesa		BOLD:AAB5106	0	0.0016	0	1
COI	Insecta	Diptera	Chironomidae	Orthocladiinae	Heterotrissocladius	marcidus	BOLD:AAF2163	0	0.0016	0	1
COI	Insecta	Diptera	Chironomidae	Orthocladiinae	Eukiefferiella		BOLD:AAI5126	0.003	0	1	0
COI	Insecta	Diptera					BOLD:ACQ9269	0	0.0016	0	1
COI	Insecta	Diptera	Chironomidae	Orthocladiinae	Cricotopus	bicinctus	BOLD:AAT9677	0.003	0	1	0
COI	Insecta	Diptera	Chironomidae				BOLD:ACB8882	0	0.0016	0	1
COI	Insecta	Diptera	Chironomidae				BOLD:ACF6427	0.003	0	1	0
COI	Insecta	Diptera	Chironomidae	Diamesinae			BOLD:ACK5496	0	0.0033	0	2
COI	Insecta	Diptera	Chloropidae	Oscinellinae			BOLD:AAN5657	0	0.0016	0	1
COI	Insecta	Diptera	Chloropidae				BOLD:ADC9928	0.003	0	1	0
COI	Insecta	Diptera	Culicidae	Culicinae			BOLD:AAF2904	0.003	0.0016	1	1
COI	Insecta	Diptera	Culicidae	Culicinae	Culex	orientalis	BOLD:ACB9306	0.009	0.0212	3	13
COI	Insecta	Diptera	Culicidae	Culicinae	Culex		BOLD:AAA4751	0.003	0	1	0
COI	Insecta	Diptera	Cylindrotomidae	Cylindrotominae	Cylindrotoma		BOLD:AAD0770	0	0.0016	0	1
COI	Insecta	Diptera	Drosophilidae	Drosophilinae	Drosophila		BOLD:AAY0127	0	0.0016	0	1
COI	Insecta	Diptera	Drosophilidae	Drosophilinae	Drosophila		BOLD:ADH1432	0	0.0016	0	1
COI	Insecta	Diptera	Drosophilidae	Steganinae	Leucophenga		BOLD:ACA1411	0	0.0016	0	1
COI	Insecta	Diptera	Ephydridae				BOLD:ACS0447	0	0.0016	0	1
COI	Insecta	Diptera	Hybotidae	Tachydromiinae	Chersodromia		BOLD:ACJ9160	0.003	0	1	0
COI	Insecta	Diptera	Keroplatidae				BOLD:AAG4943	0	0.0016	0	1
COI	Insecta	Diptera	Limoniidae	Limoniinae	Antocha	bifida	BOLD:AAW5955	0.003	0.0114	1	7
COI	Insecta	Diptera	Limoniidae	Limoniinae	Dicranomyia	frontalis	BOLD:ABA5203	0	0.0033	0	2
COI	Insecta	Diptera	Limoniidae	Limoniinae	Dicranomyia		BOLD:ABW0212	0	0.0016	0	1
COI	Insecta	Diptera	Limoniidae				BOLD:ABV4791	0	0.0016	0	1
COI	Insecta	Diptera	Limoniidae				BOLD:ACS9407	0.006	0.0049	2	3
COI	Insecta	Diptera	Limoniidae	Limoniinae	Discobola		BOLD:ABW4922	0	0.0016	0	1
COI	Insecta	Diptera	Limoniidae	Limoniinae	Libnotes		BOLD:ABW9478	0	0.0016	0	1

Primer	Class	Order	Family	Subfamily	Genus	Species	BIN	M. ussuriensis	M. ikonnikovi	M. ussuriensis	M. ikonnikovi
COI	Insecta	Diptera	Limoniidae	Limoniinae	Dicranomyia		BOLD:AAO3939	0	0.0033	0	2
COI	Insecta	Diptera	Limoniidae	Limoniinae	Dicranomyia		FINTI487-12	0	0.0049	0	3
COI	Insecta	Diptera	Limoniidae	Limoniinae	Metalimnobia		BOLD:ABU9381	0	0.0016	0	1
COI	Insecta	Diptera	Limoniidae	Limoniinae	Metalimnobia		BOLD:AAX5093	0	0.0016	0	1
COI	Insecta	Diptera	Limoniidae	Limoniinae	Rhipidia	maculata	BOLD:ACB7799	0	0.0049	0	3
COI	Insecta	Diptera	Lonchopteridae	NA	Lonchoptera		BOLD:ACB6694	0.003	0.0016	1	1
COI	Insecta	Diptera	Muscidae				BOLD:AAG1765	0	0.0033	0	2
COI	Insecta	Diptera	Muscidae				BOLD:ACD2056	0	0.0033	0	2
COI	Insecta	Diptera	Muscidae	Phaoniinae	Helina	obscurata	BOLD:AAE6931	0.003	0.0016	1	1
COI	Insecta	Diptera	Muscidae	Azeliinae	Muscina	pascuorum	BOLD:AAG1714	0	0.0016	0	1
COI	Insecta	Diptera	Muscidae				BOLD:ACR5608	0.003	0	1	0
COI	Insecta	Diptera	Muscidae				BOLD:AAX1493	0.003	0.0016	1	1
COI	Insecta	Diptera	Muscidae	Azeliinae	Hydrotaea	armipes	BOLD:AAG6908	0	0.0016	0	1
COI	Insecta	Diptera	Muscidae	Phaoniinae	Phaonia		BOLD:AAG1772	0.003	0.0033	1	2
COI	Insecta	Diptera	Muscidae	Phaoniinae	Phaonia		BOLD:AAP8142	0.003	0	1	0
COI	Insecta	Diptera	Muscidae	Azeliinae	Hydrotaea	glabricula	BOLD:ADK1195	0	0.0033	0	2
COI	Insecta	Diptera	Muscidae	Azeliinae	Potamia	littoralis	BOLD:ACQ0523	0.003	0	1	0
COI	Insecta	Diptera	Muscidae	Azeliinae	Thricops	diaphanus	BOLD:AAG1710	0	0.0016	0	1
COI	Insecta	Diptera	Mycetophilidae	Mycetophilinae	Platurocypta		BOLD:ACC5656	0	0.0016	0	1
COI	Insecta	Diptera	Pediciidae	Uliinae	Ula		BOLD:AAV1814	0.003	0.0033	1	2
COI	Insecta	Diptera	Pediciidae	Pediciinae	Dicranota		BOLD:ABW5265	0	0.0016	0	1
COI	Insecta	Diptera	Pediciidae	Uliinae	Ula	bolitophila	BOLD:ABU5946	0.003	0	1	0
COI	Insecta	Diptera	Pediciidae	Uliinae	Ula		BOLD:ABA7462	0.003	0	1	0
COI	Insecta	Diptera	Periscelididae				BOLD:ADE5755	0	0.0049	0	3
COI	Insecta	Diptera	Phoridae				BOLD:AAP6413	0.003	0	1	0
COI	Insecta	Diptera	Psychodidae				BOLD:ACO7340	0	0.0016	0	1
COI	Insecta	Diptera	Psychodidae				BOLD:ADE7332	0	0.0016	0	1
COI	Insecta	Diptera	Psychodidae				BOLD:ADE8063	0	0.0016	0	1
COI	Insecta	Diptera	Psychodidae	Psychodinae	Psychoda		BOLD:AAL7819	0.003	0	1	0
COI	Insecta	Diptera	Psychodidae	Psychodinae	Psychoda		BOLD:ACD9559	0	0.0016	0	1
COI	Insecta	Diptera	Sarcophagidae				BOLD:ACU3079	0.009	0.0033	3	2
COI	Insecta	Diptera	Sciaridae				BOLD:ACC1760	0.003	0	1	0
COI	Insecta	Diptera	Sciaridae				BOLD:ACI7364	0	0.0049	0	3
COI	Insecta	Diptera	Simuliidae	Simuliinae	Simulium		BOLD:ABA1846	0	0.0016	0	1
COI	Insecta	Diptera	Sphaeroceridae	Limosininae	Opalimosina	mirabilis	BOLD:AAN6406	0	0.0049	0	3
COI	Insecta	Diptera	Syrphidae	Eristalinae	Ferdinandea	cuprea	BOLD:AAJ0402	0	0.0065	0	4
COI	Insecta	Diptera	Tachinidae	Exoristinae	Cyzenis		BOLD:AAP4829	0	0.0016	0	1
COI	Insecta	Diptera	Tachinidae	Exoristinae	Drino		BOLD:AAB6198	0.003	0	1	0

Primer	Class	Order	Family	Subfamily	Genus	Species	BIN	M. ussuriensis	M. ikonnikovi	M. ussuriensis	M. ikonnikovi
COI	Insecta	Diptera	Tachinidae	Exoristinae			BOLD:AAN9266	0.003	0	1	0
COI	Insecta	Diptera	Tachinidae	Exoristinae	Cadurciella	tritaeniata	BOLD:AAN9651	0.003	0	1	0
COI	Insecta	Diptera	Tachinidae	Exoristinae	Phebellia	villica	BOLD:ACB0760	0.003	0	1	0
COI	Insecta	Diptera	Tachinidae				BOLD:AAZ8670	0.003	0	1	0
COI	Insecta	Diptera	Tachinidae	Exoristinae			BOLD:AAC1692	0.003	0	1	0
COI	Insecta	Diptera	Tipulidae	Tipulinae	Tipula	kuzuensis	BOLD:AAN9034	0	0.0065	0	4
COI	Insecta	Diptera	Tipulidae	Tipulinae	Tipula		BOLD:ABV3695	0	0.0016	0	1
COI	Insecta	Diptera	Tipulidae	Tipulinae	Tipula		BOLD:ABV4667	0	0.0016	0	1
COI	Insecta	Diptera	Tipulidae	Tipulinae	Tipula		TIPTW088-10	0.003	0.0049	1	3
COI	Insecta	Diptera	Tipulidae				TIPTW093-10	0.003	0.0098	1	6
COI	Insecta	Diptera	Trichoceridae	Trichocerinae	Trichocera		BOLD:ACF7745	0	0.0016	0	1
COI	Insecta	Hemiptera	Pentatomidae				BOLD:AAP3528	0.003	0	1	0
COI	Insecta	Hemiptera	Cicadellidae	Deltocephalinae			BOLD:AAG8956	0	0.0016	0	1
COI	Insecta	Hemiptera	Cicadidae	Cicadinae	Yezoterpnosia	nigricosta	BOLD:ACP9813	0.003	0	1	0
COI	Insecta	Hemiptera	Psyllidae				BOLD:ACM6872	0	0.0016	0	1
COI	Insecta	Hymenoptera	Cimbicidae	Cimbicinae	Trichiosoma		BOLD:ACJ7002	0.003	0	1	0
COI	Insecta	Hymenoptera	Cimbicidae	Cimbicinae	Trichiosoma		BOLD:ABZ2163	0.003	0	1	0
COI	Insecta	Hymenoptera	Ichneumonidae				BOLD:ADI1279	0	0.0016	0	1
COI	Insecta	Hymenoptera	Pamphiliidae	Pamphiliinae	Neurotoma	nemoralis	BOLD:AAV6346	0.003	0	1	0
COI	Insecta	Hymenoptera	Pamphiliidae	Pamphiliinae	Pamphilius		BOLD:AAK4614	0.003	0	1	0
COI	Insecta	Hymenoptera					EII067-15	0.003	0	1	0
COI	Insecta	Hymenoptera					EII104-15	0	0.0033	0	2
COI	Insecta	Hymenoptera	Tenthredinidae	Nematinae	Pristiphora		BOLD:AAK9450	0.003	0	1	0
COI	Insecta	Lepidoptera	Coleophoridae	Coleophorinae	Coleophora		BOLD:AAC8694	0	0.0016	0	1
COI	Insecta	Lepidoptera	Crambidae	Scopariinae	Eudonia	persimilis	BOLD:AAF1478	0	0.0016	0	1
COI	Insecta	Lepidoptera					BOLD:AAF8577	0	0.0033	0	2
COI	Insecta	Lepidoptera					BOLD:AAG6034	0	0.0016	0	1
COI	Insecta	Lepidoptera	Erebidae	Arctiinae			BOLD:AAH2626	0	0.0081	0	5
COI	Insecta	Lepidoptera	Gelechiidae	Gelechiinae			BOLD:AAH5654	0.003	0	1	0
COI	Insecta	Lepidoptera	Noctuidae	Noctuinae			BOLD:AAI3519	0.009	0	3	0
COI	Insecta	Lepidoptera	Erebidae				BOLD:AAL4384	0	0.0016	0	1
COI	Insecta	Lepidoptera	Crambidae	Spilomelinae	Pleuroptya/Patania	expictalis	BOLD:AAL4502	0.003	0	1	0
COI	Insecta	Lepidoptera	Erebidae	Boletobiinae	Enispa		BOLD:AAL7095	0.003	0.0033	1	2
COI	Insecta	Lepidoptera	Noctuidae	Noctuinae			BOLD:AAM0480	0	0.0016	0	1
COI	Insecta	Lepidoptera	Notodontidae	Notodontinae	Cerura		BOLD:AAM4552	0	0.0016	0	1
COI	Insecta	Lepidoptera	Noctuidae				BOLD:AAM9055	0.003	0	1	0
COI	Insecta	Lepidoptera	Noctuidae	Noctuinae			BOLD:AAM9761	0.003	0.0033	1	2
COI	Insecta	Lepidoptera	Crambidae	Pyraustinae	Goniorhynchus		BOLD:AAN3555	0	0.0033	0	2

Primer	Class	Order	Family	Subfamily	Genus	Species	BIN	M. ussuriensis	M. ikonnikovi	M. ussuriensis	M. ikonnikovi
COI	Insecta	Lepidoptera	Hesperiidae				BOLD:AAN7248	0	0.0016	0	1
COI	Insecta	Lepidoptera	Noctuidae				BOLD:AAP1728	0	0.0016	0	1
COI	Insecta	Lepidoptera	Geometridae	Ennominae			BOLD:AAP2372	0.003	0	1	0
COI	Insecta	Lepidoptera	Gelechiidae				BOLD:AAQ1460	0	0.0016	0	1
COI	Insecta	Lepidoptera	Crambidae	Spilomelinae			BOLD:AAQ2087	0	0.0049	0	3
COI	Insecta	Lepidoptera	Geometridae	Sterrhinae	Scopula		BOLD:AAV8961	0	0.0016	0	1
COI	Insecta	Lepidoptera	Erebidae	Arctiinae			BOLD:AAY5858	0	0.0016	0	1
COI	Insecta	Lepidoptera	Limacodidae				BOLD:AAZ8422	0	0.0016	0	1
COI	Insecta	Lepidoptera	Tortricidae	Olethreutinae	Rhopobota		BOLD:ABA8634	0.003	0.0016	1	1
COI	Insecta	Lepidoptera	Geometridae	Larentiinae	Leptostegna		BOLD:ABU5788	0.003	0	1	0
COI	Insecta	Lepidoptera	Sphingidae	Smerinthinae	Marumba	gaschkewitschii	BOLD:ABU7231	0.003	0	1	0
COI	Insecta	Lepidoptera					BOLD:ABU9226	0.006	0	2	0
COI	Insecta	Lepidoptera	Tortricidae				BOLD:ABW0658	0	0.0016	0	1
COI	Insecta	Lepidoptera	Erebidae	Arctiinae	Cyana		BOLD:ABX3752	0.003	0.0114	1	7
COI	Insecta	Lepidoptera	Erebidae	Erebinae	Catocala		BOLD:ABZ3190	0.003	0	1	0
COI	Insecta	Lepidoptera	Erebidae	Arctiinae			BOLD:ACB1726	0	0.0016	0	1
COI	Insecta	Lepidoptera	Geometridae	Geometrinae			BOLD:ACE9755	0	0.0016	0	1
COI	Insecta	Lepidoptera	Autostichidae				BOLD:ACF7812	0	0.0016	0	1
COI	Insecta	Lepidoptera	Cosmopterigidae				BOLD:ACJ1842	0.003	0.0016	1	1
COI	Insecta	Lepidoptera	Gelechiidae	Gelechiinae	Sophronia		BOLD:ACL1953	0	0.0016	0	1
COI	Insecta	Lepidoptera	Geometridae	Ennominae			BOLD:ACL6993	0	0.0033	0	2
COI	Insecta	Lepidoptera					BOLD:ACM3459	0	0.0049	0	3
COI	Insecta	Lepidoptera	Elachistidae				BOLD:ACM9615	0	0.0033	0	2
COI	Insecta	Lepidoptera	Tortricidae				BOLD:ACN0142	0.003	0.0033	1	2
COI	Insecta	Lepidoptera	Gelechiidae				BOLD:ACN0185	0	0.0098	0	6
COI	Insecta	Lepidoptera	Tortricidae	Tortricinae	Choristoneura		BOLD:ACN0625	0.003	0.0065	1	4
COI	Insecta	Lepidoptera	Erebidae	Herminiinae			BOLD:ACO1434	0.003	0	1	0
COI	Insecta	Lepidoptera	Lasiocampidae	Poecilocampinae			BOLD:ACP2255	0	0.0033	0	2
COI	Insecta	Lepidoptera	Gelechiidae				BOLD:ACR6168	0.003	0.0016	1	1
COI	Insecta	Lepidoptera	Stathmopodidae	NA	Stathmopoda		BOLD:ACV9615	0.003	0	1	0
COI	Insecta	Lepidoptera					BOLD:ACW1375	0.000	0.0081	0	5
COI	Insecta	Lepidoptera					BOLD:ACX5590	0.003	0	1	0
COI	Insecta	Lepidoptera	Crambidae				BOLD:ACX6648	0.003	0.0016	1	1
COI	Insecta	Lepidoptera					BOLD:ADF8756	0	0.0049	0	3
COI	Insecta	Lepidoptera					BOLD:ADG3051	0	0.0016	0	1
COI	Insecta	Lepidoptera					BOLD:ADI1550	0	0.0016	0	1
COI	Insecta	Lepidoptera	Tortricidae	Tortricinae	Acleris	paradiseana	BOLD:ADI2826	0	0.0016	0	1
COI	Insecta	Lepidoptera					BOLD:ADI2961	0	0.0098	0	6

Primer	Class	Order	Family	Subfamily	Genus	Species	BIN	M. ussuriensis	M. ikonnikovi	M. ussuriensis	M. ikonnikovi
COI	Insecta	Lepidoptera					BOLD:ADI4331	0	0.0016	0	1
COI	Insecta	Lepidoptera	Geometridae	Sterrhinae	Scopula		BOLD:ADI7455	0	0.0033	0	2
COI	Insecta	Lepidoptera	Geometridae	Sterrhinae	Scopula		BOLD:ADJ2221	0.003	0.0098	1	6
COI	Insecta	Lepidoptera					GWOSS664-11	0.006	0	2	0
COI	Insecta	Lepidoptera	Geometridae	Geometrinae			NAGEO164-09	0	0.0016	0	1
COI	Insecta	Lepidoptera	Autostichidae	Autostichinae			BOLD:AAC7842	0	0.0016	0	1
COI	Insecta	Lepidoptera	Blastobasidae	Blastobasinae	Hypatopa		BOLD:AAB9632	0	0.0033	0	2
COI	Insecta	Lepidoptera	Blastobasidae	Blastobasinae			BOLD:AAX6193	0	0.0016	0	1
COI	Insecta	Lepidoptera	Coleophoridae	Coleophorinae	Coleophora		BOLD:AAB8487	0	0.0033	0	2
COI	Insecta	Lepidoptera	Crambidae	Pyraustinae	Pyrausta/ Goniorhynchus		BOLD:AAD0032	0	0.0016	0	1
COI	Insecta	Lepidoptera					BOLD:ACM1938	0.003	0.0033	1	2
COI	Insecta	Lepidoptera	Crambidae	Acentropinae	Nymphula/Elophila		BOLD:AAF2874	0.003	0	1	0
COI	Insecta	Lepidoptera	Crambidae	Spilomelinae	Palpita		BOLD:AAD6468	0.025	0.0098	8	6
COI	Insecta	Lepidoptera	Crambidae	Spilomelinae			BOLD:AAB6255	0	0.0016	0	1
COI	Insecta	Lepidoptera	Crambidae	Spilomelinae	Pleuroptya/ Patania		BOLD:AAB6257	0	0.0016	0	1
COI	Insecta	Lepidoptera	Depressariidae				BOLD:AAY5033	0	0.0016	0	1
COI	Insecta	Lepidoptera	Depressariidae	Depressariinae	Agonopterix		BOLD:AAF7176	0	0.0033	0	2
COI	Insecta	Lepidoptera	Depressariidae	Depressariinae	Depressaria		BOLD:ACN1500	0	0.0033	0	2
COI	Insecta	Lepidoptera	Depressariidae				BOLD:ACE7937	0.003	0	1	0
COI	Insecta	Lepidoptera	Drepanidae	Thyatirinae	Tetheella	fluctuosa	BOLD:AAD3647	0	0.0033	0	2
COI	Insecta	Lepidoptera	Erebidae				BOLD:AAB7538	0.009	0	3	0
COI	Insecta	Lepidoptera	Erebidae	Erebinae	Catocala		BOLD:AAE9541	0.003	0.0016	1	1
COI	Insecta	Lepidoptera	Erebidae	Arctiinae			BOLD:AAC3572	0	0.0049	0	3
COI	Insecta	Lepidoptera	Erebidae	Hypeninae			BOLD:AAC7130	0	0.0016	0	1
COI	Insecta	Lepidoptera	Erebidae	Erebinae	Ercheia		BOLD:AAD8285	0.003	0	1	0
COI	Insecta	Lepidoptera	Erebidae	Erebinae	Thyas	juno	BOLD:AAF1405	0.003	0	1	0
COI	Insecta	Lepidoptera	Erebidae	Erebinae			BOLD:ABX5513	0.003	0	1	0
COI	Insecta	Lepidoptera	Erebidae	Arctiinae			BOLD:ACE8889	0	0.0081	0	5
COI	Insecta	Lepidoptera	Erebidae				BOLD:ACT9815	0.003	0	1	0
COI	Insecta	Lepidoptera	Erebidae				BOLD:ADF0678	0.003	0.0016	1	1
COI	Insecta	Lepidoptera	Erebidae	Herminiinae	Hydrillodes		BOLD:ACU0270	0.003	0.0049	1	3
COI	Insecta	Lepidoptera					BOLD:AAV8834	0.003	0.0033	1	2
COI	Insecta	Lepidoptera	Erebidae	Lymantriinae	Lymantria	dispar	BOLD:AAA2052	0.003	0	1	0
COI	Insecta	Lepidoptera	Erebidae	Lymantriinae	Lymantria	monacha	BOLD:AAA5537	0.006	0.0033	2	2
COI	Insecta	Lepidoptera	Erebidae	Pangraptinae	Pangrapta		BOLD:ADF1765	0.003	0	1	0
COI	Insecta	Lepidoptera	Gelechiidae	Gelechiinae	Gelechia	cuneatella	BOLD:AAF5086	0	0.0033	0	2
COI	Insecta	Lepidoptera	Gelechiidae	Gelechiinae	Psoricoptera	gibbosella	BOLD:AAD0608	0.009	0.0081	3	5
COI	Insecta	Lepidoptera	Gelechiidae				BOLD:ABA1140	0.003	0.0033	1	2

Primer	Class	Order	Family	Subfamily	Genus	Species	BIN	M. ussuriensis	M. ikonnikovi	M. ussuriensis	M. ikonnikovi
COI	Insecta	Lepidoptera	Geometridae	Ennominae			BOLD:ACG8719	0.003	0	1	0
COI	Insecta	Lepidoptera	Geometridae	Ennominae	Cleora	insolita	BOLD:ACJ3813	0.006	0	2	0
COI	Insecta	Lepidoptera	Geometridae	Ennominae			BOLD:AAC6655	0.003	0	1	0
COI	Insecta	Lepidoptera	Geometridae	Ennominae	Deileptenia	ribeata	BOLD:AAC3800	0	0.0016	0	1
COI	Insecta	Lepidoptera	Geometridae	Ennominae	Ectropis		BOLD:AAB5227	0.003	0.0016	1	1
COI	Insecta	Lepidoptera	Geometridae	Ennominae	Ectropis	crepuscularia	BOLD:AAA2076	0.003	0	1	0
COI	Insecta	Lepidoptera	Geometridae	Ennominae	Endropiodes		BOLD:AAF7479	0.003	0	1	0
COI	Insecta	Lepidoptera	Geometridae	Larentiinae	Gandaritis	fixseni	BOLD:AAY1335	0.003	0	1	0
COI	Insecta	Lepidoptera	Geometridae	Larentiinae	Gandaritis		BOLD:AAB5983	0	0.0016	0	1
COI	Insecta	Lepidoptera	Geometridae	Geometrinae	Geometra		BOLD:AAB2011	0	0.0016	0	1
COI	Insecta	Lepidoptera	Geometridae	Ennominae	Protoboarmia		BOLD:AAA2077	0	0.0016	0	1
COI	Insecta	Lepidoptera	Geometridae	Larentiinae	Eupithecia		BOLD:AAA2083	0.003	0	1	0
COI	Insecta	Lepidoptera	Geometridae	Sterrhinae	Cyclophora		BOLD:AAD6021	0.003	0.0033	1	2
COI	Insecta	Lepidoptera	Geometridae	Geometrinae			BOLD:AAE5087	0.003	0.0081	1	5
COI	Insecta	Lepidoptera	Geometridae	Ennominae			BOLD:AAE6522	0	0.0033	0	2
COI	Insecta	Lepidoptera	Geometridae	Ennominae	Menophra	senilis	BOLD:AAF3823	0.003	0	1	0
COI	Insecta	Lepidoptera	Geometridae	Ennominae			BOLD:AAL8521	0.003	0	1	0
COI	Insecta	Lepidoptera	Geometridae	Ennominae			BOLD:AAP9222	0	0.0016	0	1
COI	Insecta	Lepidoptera	Geometridae				BOLD:AAZ1901	0	0.0033	0	2
COI	Insecta	Lepidoptera	Geometridae	Ennominae			BOLD:ABU6285	0	0.0016	0	1
COI	Insecta	Lepidoptera	Geometridae	Geometrinae	Jodis	putata	BOLD:ABZ4040	0.003	0.0016	1	1
COI	Insecta	Lepidoptera	Geometridae	Larentiinae			BOLD:ACB9627	0.003	0.0016	1	1
COI	Insecta	Lepidoptera	Geometridae				BOLD:ADF1852	0.009	0	3	0
COI	Insecta	Lepidoptera	Geometridae	Sterrhinae	Scopula		LNAUS1341-13	0	0.0065	0	4
COI	Insecta	Lepidoptera	Geometridae	Ennominae	Hypomecis	punctinalis	BOLD:ACA2461	0.012	0	4	0
COI	Insecta	Lepidoptera	Geometridae	Ennominae			BOLD:ACS9064	0.003	0	1	0
COI	Insecta	Lepidoptera	Geometridae	Ennominae			BOLD:AAB6560	0.003	0	1	0
COI	Insecta	Lepidoptera	Geometridae	Ennominae	Ourapteryx	maculicaudaria	BOLD:AAW9443	0.003	0	1	0
COI	Insecta	Lepidoptera	Geometridae	Ennominae			BOLD:AAA2521	0	0.0016	0	1
COI	Insecta	Lepidoptera	Geometridae	Ennominae	Phthonosema	tendinosaria	BOLD:AAF6489	0.006	0	2	0
COI	Insecta	Lepidoptera	Gracillariidae	Gracillariinae	Caloptilia	cf. heringi	BOLD:ADK1669	0	0.0033	0	2
COI	Insecta	Lepidoptera	Gracillariidae	Gracillariinae	Caloptilia	hidakensis	BOLD:AAK1674	0	0.0016	0	1
COI	Insecta	Lepidoptera	Gracillariidae	Gracillariinae	Caloptilia		BOLD:ACL6329	0	0.0016	0	1
COI	Insecta	Lepidoptera	Gracillariidae	Gracillariinae			BOLD:ADK2510	0	0.0016	0	1
COI	Insecta	Lepidoptera	Hepialidae	Hepialinae			BOLD:AAO2948	0.003	0	1	0
COI	Insecta	Lepidoptera	Hepialidae	Hepialinae			BOLD:ACN8703	0	0.0016	0	1
COI	Insecta	Lepidoptera	Hesperiidae	Coeliadinae	Burara/Bibasis	aquilina	BOLD:ACD6545	0.009	0	3	0
COI	Insecta	Lepidoptera	Hesperiidae	Hesperiinae			LIMBC545-11	0.006	0.0065	2	4

Primer	Class	Order	Family	Subfamily	Genus	Species	BIN	M. ussuriensis	M. ikonnikovi	M. ussuriensis	M. ikonnikovi
COI	Insecta	Lepidoptera	Hesperiidae	Hesperiinae	Thoressa	varia	BOLD:ADK0840	0.003	0	1	0
COI	Insecta	Lepidoptera	Lasiocampidae	Lasiocampinae	Euthrix	potatoria	BOLD:AAC1584	0.003	0.0049	1	3
COI	Insecta	Lepidoptera	Lecithoceridae				BOLD:AAD6680	0	0.0016	0	1
COI	Insecta	Lepidoptera	Limacodidae	Limacodinae			BOLD:ACE6221	0	0.0049	0	3
COI	Insecta	Lepidoptera	Limacodidae	Limacodinae			BOLD:AAA9203	0.003	0.0033	1	2
COI	Insecta	Lepidoptera	Limacodidae				BOLD:ACC2517	0.003	0.0016	1	1
COI	Insecta	Lepidoptera	Limacodidae	Limacodinae	Parasa		BOLD:AAM0823	0.003	0.0065	1	4
COI	Insecta	Lepidoptera	Limacodidae	Limacodinae	Phrixolepia		BOLD:AAV5028	0	0.0016	0	1
COI	Insecta	Lepidoptera	Lycaenidae	Theclinae	Rapala		BOLD:AAL1375	0.003	0.0016	1	1
COI	Insecta	Lepidoptera	Noctuidae	Acronictinae	Acronicta		BOLD:ADK0044	0.003	0	1	0
COI	Insecta	Lepidoptera	Noctuidae	Pantheinae	Anacronicta		BOLD:AAK1458	0.003	0	1	0
COI	Insecta	Lepidoptera	Noctuidae	Noctuinae	Apamea	helva	BOLD:AAC5412	0.003	0	1	0
COI	Insecta	Lepidoptera	Noctuidae	Noctuinae	Athetis		BOLD:AAV0425	0.003	0	1	0
COI	Insecta	Lepidoptera	Noctuidae	Acronictinae	Belciades	niveola	BOLD:ACU0724	0.003	0	1	0
COI	Insecta	Lepidoptera	Noctuidae	Amphipyrinae	Brachionycha	nubeculosa	BOLD:AAE0860	0.003	0	1	0
COI	Insecta	Lepidoptera					BOLD:ADF8757	0.009	0	3	0
COI	Insecta	Lepidoptera	Noctuidae				BOLD:ACT9151	0.003	0	1	0
COI	Insecta	Lepidoptera	Noctuidae	Noctuinae			BOLD:ACJ1078	0.006	0	2	0
COI	Insecta	Lepidoptera	Noctuidae	Oncocnemidinae			BOLD:AAE4319	0.003	0	1	0
COI	Insecta	Lepidoptera	Noctuidae	Plusiinae			BOLD:AAE7504	0.003	0	1	0
COI	Insecta	Lepidoptera	Noctuidae	Amphipyrinae			BOLD:AAW4866	0.003	0.0016	1	1
COI	Insecta	Lepidoptera	Noctuidae	Noctuinae	Leucania		BOLD:AAJ2503	0.003	0	1	0
COI	Insecta	Lepidoptera	Noctuidae	Noctuinae	Lithophane	socia	BOLD:AAE6607	0.003	0	1	0
COI	Insecta	Lepidoptera	Noctuidae	Noctuinae	Spodoptera	exigua	BOLD:AAA6644	0	0.0049	0	3
COI	Insecta	Lepidoptera	Noctuidae	Noctuinae	Spodoptera	cilium	BOLD:AAC8279	0	0.0049	0	3
COI	Insecta	Lepidoptera	Noctuidae	Amphipyrinae			BOLD:AAH5319	0	0.0016	0	1
COI	Insecta	Lepidoptera					BOLD:ADG3545	0.003	0	1	0
COI	Insecta	Lepidoptera					BOLD:AAB6211	0.003	0	1	0
COI	Insecta	Lepidoptera	Noctuidae	Noctuinae			BOLD:AAB6980	0.006	0	2	0
COI	Insecta	Lepidoptera	Noctuidae	Noctuinae	Xylena		BOLD:AAE4735	0.003	0.0065	1	4
COI	Insecta	Lepidoptera	Nolidae	Chloephorinae	Nycteola		BOLD:AAE2641	0	0.0016	0	1
COI	Insecta	Lepidoptera	Nolidae	Nolinae	Nola	confusalis	BOLD:AAB5563	0	0.0016	0	1
COI	Insecta	Lepidoptera	Nolidae	Nolinae			BOLD:AAL7275	0	0.0049	0	3
COI	Insecta	Lepidoptera	Nolidae	Chloephorinae	Pseudoips	prasinana	BOLD:AAB8807	0	0.0033	0	2
COI	Insecta	Lepidoptera	Notodontidae				BOLD:AAM3801	0	0.0016	0	1
COI	Insecta	Lepidoptera	Notodontidae	Notodontinae			BOLD:AAL6465	0.003	0	1	0
COI	Insecta	Lepidoptera	Notodontidae	Heterocampinae			BOLD:AAM4825	0	0.0016	0	1
COI	Insecta	Lepidoptera	Notodontidae	Notodontinae	Notodonta		BOLD:AAC1146	0.003	0	1	0

Primer	Class	Order	Family	Subfamily	Genus	Species	BIN	M. ussuriensis	M. ikonnikovi	M. ussuriensis	M. ikonnikovi
COI	Insecta	Lepidoptera	Notodontidae	Notodontinae	Shaka		BOLD:ACJ1076	0	0.0016	0	1
COI	Insecta	Lepidoptera	Notodontidae	Heterocampinae	Stauropus	fagi	BOLD:AAD0646	0.003	0	1	0
COI	Insecta	Lepidoptera	Nymphalidae	Heliconiinae	Boloria		BOLD:AAB9155	0	0.0033	0	2
COI	Insecta	Lepidoptera	Oecophoridae	Oecophorinae			BOLD:AAF9734	0	0.0033	0	2
COI	Insecta	Lepidoptera	Papilionidae	Papilioninae	Papilio		BOLD:AAI5285	0.003	0	1	0
COI	Insecta	Lepidoptera	Pyralidae	Pyralinae	Endotricha	olivacealis	BOLD:ACI9403	0	0.0016	0	1
COI	Insecta	Lepidoptera	Saturniidae				BOLD:AAA5428	0	0.0016	0	1
COI	Insecta	Lepidoptera	Saturniidae	Saturniinae	Saturnia		BOLD:AAB9581	0.025	0.0033	8	2
COI	Insecta	Lepidoptera	Saturniidae	Saturniinae	Saturnia	jonasii	BOLD:AAD1452	0.003	0	1	0
COI	Insecta	Lepidoptera	Sphingidae	Smerinthinae	Callambulyx		BOLD:ABY4871	0.003	0	1	0
COI	Insecta	Lepidoptera	Sphingidae	Macroglossinae			BOLD:AAB0931	0	0.0016	0	1
COI	Insecta	Lepidoptera	Sphingidae	Sphinginae	Sphinx	ligustri	BOLD:AAB6107	0.003	0	1	0
COI	Insecta	Lepidoptera	Sphingidae	Sphinginae	Sphinx		BOLD:ACF5246	0.003	0	1	0
COI	Insecta	Lepidoptera	Stathmopodidae	NA	Stathmopoda	pedella	BOLD:AAD4282	0	0.0016	0	1
COI	Insecta	Lepidoptera	Tineidae	Scardiinae	Morophaga	bucephala	BOLD:AAG8510	0	0.0016	0	1
COI	Insecta	Lepidoptera	Tortricidae	Tortricinae			BOLD:AAC3136	0	0.0016	0	1
COI	Insecta	Lepidoptera	Tortricidae	Tortricinae	Archips	betulana	BOLD:ACM3437	0.003	0.0081	1	5
COI	Insecta	Lepidoptera	Tortricidae	Tortricinae	Archips		BOLD:AAB5839	0	0.0033	0	2
COI	Insecta	Lepidoptera	Tortricidae	Tortricinae	Choristoneura	diversana	BOLD:AAD8048	0.006	0.0130	2	8
COI	Insecta	Lepidoptera	Tortricidae	Tortricinae			BOLD:ACT2120	0.003	0.0016	1	1
COI	Insecta	Lepidoptera	Tortricidae	Tortricinae	Dichelia		ANICU1645-11	0.003	0	1	0
COI	Insecta	Lepidoptera	Tortricidae	Tortricinae	Eana	incanana	BOLD:AAD7476	0	0.0016	0	1
COI	Insecta	Lepidoptera	Tortricidae	Olethreutinae	Epinotia		BOLD:AAE1784	0	0.0033	0	2
COI	Insecta	Lepidoptera	Tortricidae	Olethreutinae	Epinotia	nisella	BOLD:AAA7530	0	0.0016	0	1
COI	Insecta	Lepidoptera	Tortricidae	Olethreutinae	Eudemis	porphyrana	BOLD:AAC6854	0	0.0033	0	2
COI	Insecta	Lepidoptera	Tortricidae	Olethreutinae	Gypsonoma	dealbana	BOLD:AAB0380	0	0.0016	0	1
COI	Insecta	Lepidoptera	Tortricidae	Olethreutinae	Lobesia		BOLD:ABV8007	0.003	0.0016	1	1
COI	Insecta	Lepidoptera	Tortricidae	Olethreutinae			BOLD:ABZ7645	0	0.0016	0	1
COI	Insecta	Lepidoptera	Tortricidae	Olethreutinae	Olethreutes		BOLD:ACS0054	0	0.0065	0	4
COI	Insecta	Lepidoptera	Tortricidae	Tortricinae	Pandemis	cinnamomeana	BOLD:AAD0575	0.003	0.0016	1	1
COI	Insecta	Lepidoptera	Tortricidae	Tortricinae	Pandemis	corylana	BOLD:AAC5400	0.003	0	1	0
COI	Insecta	Lepidoptera	Tortricidae	Olethreutinae	Phiaris		BOLD:AAJ2026	0	0.0016	0	1
COI	Insecta	Lepidoptera	Tortricidae	Tortricinae	Ptycholoma	lecheanum	BOLD:AAD3264	0	0.0033	0	2
COI	Insecta	Lepidoptera	Tortricidae	Olethreutinae	Rhopobota	naevana	BOLD:AAA9812	0	0.0065	0	4
COI	Insecta	Lepidoptera	Tortricidae	Olethreutinae			BOLD:AAA0213	0.003	0	1	0
COI	Insecta	Lepidoptera	Tortricidae	Olethreutinae	Spilonota	laricana	BOLD:AAA7739	0	0.0016	0	1
COI	Insecta	Lepidoptera	Tortricidae	Tortricinae	Archips	crataeganus	BOLD:AAD6620	0	0.0049	0	3
COI	Insecta	Lepidoptera					BOLD:AAH4639	0	0.0016	0	1

Primer	Class	Order	Family	Subfamily	Genus	Species	BIN	M. ussuriensis	M. ikonnikovi	M. ussuriensis	M. ikonnikovi
COI	Insecta	Lepidoptera	Tortricidae	Olethreutinae	Epinotia		BOLD:AAN7425	0	0.0016	0	1
COI	Insecta	Lepidoptera	Tortricidae	Olethreutinae			BOLD:AAV9504	0	0.0016	0	1
COI	Insecta	Lepidoptera	Tortricidae				BOLD:ADE9961	0	0.0016	0	1
COI	Insecta	Lepidoptera	Tortricidae	Tortricinae			BOLD:ACP8754	0.003	0.0033	1	2
COI	Insecta	Lepidoptera	Tortricidae	Olethreutinae	Zeiraphera	rufimitrana	BOLD:AAM3356	0	0.0033	0	2
COI	Insecta	Lepidoptera	Uraniidae	Uraniinae			BOLD:ACF4699	0	0.0049	0	3
COI	Insecta	Lepidoptera	Yponomeutidae	Yponomeutinae			BOLD:AAE4109	0.003	0.0016	1	1
COI	Insecta	Lepidoptera	Ypsolophidae	Ypsolophinae	Ypsolopha	vittella	BOLD:AAD9548	0	0.0016	0	1
COI	Insecta	Lepidoptera					BOLD:AAD3218	0.003	0.0016	1	1
COI	Insecta	Mecoptera	Panorpidae	Panorpinae	Panorpa	pryeri	BOLD:ACA3726	0	0.0016	0	1
COI	Insecta	Neuroptera	Hemerobiidae	Drepanepteryginae	Drepanepteryx		BOLD:ACT2374	0.003	0	1	0
COI	Insecta	Neuroptera	Hemerobiidae	Hemerobiinae	Hemerobius		BOLD:AAG0897	0.006	0.0016	2	1
COI	Insecta	Neuroptera	Hemerobiidae	Hemerobiinae	Hemerobius		BOLD:ABZ0149	0	0.0016	0	1
COI	Insecta	Neuroptera	Hemerobiidae	Hemerobiinae	Hemerobius	fenestratus	BOLD:AAU3559	0.006	0.0081	2	5
COI	Insecta	Neuroptera	Hemerobiidae	Hemerobiinae	Hemerobius	simulans	BOLD:ACM1832	0.003	0.0049	1	3
COI	Insecta	Neuroptera	Hemerobiidae	Hemerobiinae			BOLD:ABU9030	0.003	0.0016	1	1
COI	Insecta	Neuroptera	Osmylidae				INRMA502-12	0	0.0016	0	1
COI	Insecta	Plecoptera	Nemouridae	Amphinemurinae			BOLD:AAL6222	0	0.0016	0	1
COI	Insecta	Psocodea	Psocidae	Psocinae	Psococerastis		BOLD:ACC5474	0	0.0016	0	1
COI	Insecta	Trichoptera	Lepidostomatidae	Lepidostomatinae	Lepidostoma	elongatum	RUSST089-12	0	0.0016	0	1
COI	Insecta	Trichoptera	Psychomyiidae	Psychomyiinae	Tinodes	higashiyamanus	BOLD:ACD6706	0	0.0016	0	1
COI	Insecta	Trichoptera	Thremmatidae	Thremmatinae	Neophylax	ussuriensis	BOLD:AAG9568	0	0.0049	0	3
16S	Arachnida	Opiliones	Sclerosomatidae	Leiobuninae	Leiobunum	tohokuense	NA	0	0.0016	0	1
16S	Insecta	Coleoptera	Elateridae	Elaterinae	Agriotes		NA	0.003	0	1	0
16S	Insecta	Coleoptera	Scarabaeidae	Rutelinae	Anomala		NA	0.003	0	1	0
16S	Insecta	Coleoptera	Curculionidae	Curculioninae	Curculio		NA	0.003	0	1	0
16S	Insecta	Coleoptera	Cerambycidae	Lamiinae	Eutetrapha		NA	0.003	0	1	0
16S	Insecta	Coleoptera	Tenebrionidae	Alleculinae	Hymenalia		NA	0.003	0.0033	1	2
16S	Insecta	Coleoptera	Scarabaeidae				NA	0.006	0.0016	2	1
16S	Insecta	Coleoptera	Scarabaeidae				NA	0	0.0016	0	1
16S	Insecta	Coleoptera	Melandryidae	Malandryinae	Phloiotrya	planiuscula	NA	0	0.0033	0	2
16S	Insecta	Coleoptera	Scarabaeidae				NA	0.012	0.0016	4	1
16S	Insecta	Coleoptera	Cantharidae	Cantharinae	Podabrus		NA	0	0.0033	0	2
16S	Insecta	Coleoptera	Chrysomelidae				NA	0.003	0	1	0
16S	Insecta	Dermaptera	Forficulidae	Anechurinae	Anechura	harmandi	NA	0.022	0	7	0
16S	Insecta	Dermaptera	Forficulidae	Forficulinae	Forficula	mikado	NA	0.009	0	3	0
16S	Insecta	Diptera	Mycetophilidae	Gnoristinae	Boletina		NA	0	0.0016	0	1
16S	Insecta	Diptera	Tachinidae	Exoristinae	Botria	japonica	NA	0	0.0016	0	1

Primer	Class	Order	Family	Subfamily	Genus	Species	BIN	M. ussuriensis	M. ikonnikovi	M. ussuriensis	M. ikonnikovi
16S	Insecta	Diptera	Sciaridae	NA	Chaetosciara		NA	0	0.0016	0	1
16S	Insecta	Diptera	Calliphoridae	Chrysomyinae	Chrysomya		NA	0	0.0016	0	1
16S	Insecta	Diptera	Empidoidea (Superfamily)				NA	0	0.0016	0	1
16S	Insecta	Diptera	Culicidae	Culicinae	Culex		NA	0.003	0	1	0
16S	Insecta	Diptera	Culicidae	Culicinae	Culex	tritaeniorhynchus	NA	0	0.0065	0	4
16S	Insecta	Diptera	Limoniidae	Limoniinae	Dicranomyia		NA	0	0.0016	0	1
16S	Insecta	Diptera	Limoniidae	Limoniinae	Dicranomyia		NA	0	0.0081	0	5
16S	Insecta	Diptera	Limoniidae	Limoniinae	Dicranomyia		NA	0	0.0016	0	1
16S	Insecta	Diptera	Limoniidae	Limoniinae	Dicranomyia		NA	0	0.0065	0	4
16S	Insecta	Diptera	Tachinidae	Exoristinae	Eumea	linearicornis	NA	0.003	0	1	0
16S	Insecta	Diptera	Mycetophilidae	Mycetophilinae	Exechia		NA	0	0.0033	0	2
16S	Insecta	Diptera	Mycetophilidae	Mycetophilinae	Exechia		NA	0	0.0033	0	2
16S	Insecta	Diptera	Tachinidae	Exoristinae	Gonia	chinensis	NA	0.003	0	1	0
16S	Insecta	Diptera	Limoniidae				NA	0	0.0016	0	1
16S	Insecta	Diptera	Chironomidae	Chironominae	Micropsectra		NA	0	0.0016	0	1
16S	Insecta	Diptera	Chironomidae	Chironominae	Micropsectra		NA	0	0.0016	0	1
16S	Insecta	Diptera	Mycetophilidae	Mycetophilinae	Mycetophila		NA	0	0.0016	0	1
16S	Insecta	Diptera	Mycetophilidae	Mycetophilinae	Mycetophila	fungorum	NA	0	0.0049	0	3
16S	Insecta	Diptera	Mycetophilidae	Mycomyinae	Mycomya		NA	0	0.0016	0	1
16S	Insecta	Diptera					NA	0.003	0	1	0
16S	Insecta	Diptera	Empidoidea (Superfamily)				NA	0	0.0016	0	1
16S	Insecta	Diptera	Limoniidae				NA	0.003	0.0212	1	13
16S	Insecta	Diptera	Psychodidae				NA	0.003	0.0114	1	7
16S	Insecta	Diptera	Rhagionidae				NA	0	0.0016	0	1
16S	Insecta	Diptera	Tipulidae				NA	0	0.0049	0	3
16S	Insecta	Diptera	Culicidae	Culicinae	Ochlerotatus		NA	0.003	0.0016	1	1
16S	Insecta	Diptera	Pediciidae	Pediciinae	Pedicia		NA	0	0.0081	0	5
16S	Insecta	Diptera	Rhinophoridae				NA	0.003	0	1	0
16S	Insecta	Diptera	Mycetophilidae	Mycetophilinae	Pseudobrachypeza		NA	0	0.0016	0	1
16S	Insecta	Diptera	Phoridae	Phorinae	Stichillus		NA	0.003	0.0016	1	1
16S	Insecta	Diptera	Tachinidae	Exoristinae	Suensonomyia	nudinerva	NA	0.003	0	1	0
16S	Insecta	Diptera	Mycetophilidae				NA	0	0.0016	0	1
16S	Insecta	Diptera	Muscidae	Azeliinae	Thricops		NA	0	0.0016	0	1
16S	Insecta	Diptera	Tipulidae	Tipulinae	Tipula		NA	0.006	0.0098	2	6
16S	Insecta	Diptera	Tipulidae	Tipulinae	Tipula		NA	0.003	0.0065	1	4
16S	Insecta	Diptera	Mycetophilidae	Mycetophilinae			NA	0	0.0016	0	1
16S	Insecta	Ephemeroptera	Heptageniidae	Rhithrogeniinae	Rhithrogena		NA	0	0.0016	0	1
16S	Insecta	Ephemeroptera	Heptageniidae	Rhithrogeniinae	Rhithrogena		NA	0	0.0016	0	1

Primer	Class	Order	Family	Subfamily	Genus	Species	BIN	M. ussuriensis	M. ikonnikovi	M. ussuriensis	M. ikonnikovi
16S	Insecta	Hemiptera	Miridae	Mirinae	Adelphocoris		NA	0	0.0016	0	1
16S	Insecta	Hemiptera	Miridae	Deraeocorinae	Alloeotomus	simplus	NA	0	0.0016	0	1
16S	Insecta	Hemiptera	Miridae	Mirinae	Arbolygus	rubripes	NA	0.006	0.0000	2	0
16S	Insecta	Hemiptera	Acanthosomatidae	Acanthosomatinae	Elasmucha	signoreti	NA	0.003	0	1	0
16S	Insecta	Hemiptera	Miridae	Mirinae	Phytocoris		NA	0	0.0016	0	1
16S	Insecta	Hemiptera	Psyllidae	Psyllinae	Psylla		NA	0.003	0.0016	1	1
16S	Insecta	Hemiptera	Psyllidae	Psyllinae	Psylla		NA	0.006	0.0016	2	1
16S	Insecta	Hemiptera	Aphididae	Calaphidinae	Symydobius	kabae	NA	0	0.0016	0	1
16S	Insecta	Hymenoptera	Braconidae	Rogadinae	Aleiodes		NA	0.003	0	1	0
16S	Insecta	Hymenoptera	Ichneumonidae	Campopleginae	Hyposoter		NA	0.003	0	1	0
16S	Insecta	Hymenoptera	Braconidae	Microgastrinae	Microplitis		NA	0.003	0	1	0
16S	Insecta	Hymenoptera					NA	0.003	0	1	0
16S	Insecta	Hymenoptera	Formicidae				NA	0	0.0016	0	1
16S	Insecta	Hymenoptera	Tenthredinidae	Nematinae	Nematinus	luteus	NA	0.003	0	1	0
16S	Insecta	Lepidoptera	Geometridae	Ennominae	Arichanna	melanaria	NA	0.003	0	1	0
16S	Insecta	Lepidoptera	Noctuidae				NA	0.006	0	2	0
16S	Insecta	Lepidoptera	Tortricidae	Tortricinae			NA	0	0.0033	0	2
16S	Insecta	Trichoptera	Limnephilidae	Dicosmoecinae	Dicosmoecus		NA	0	0.0065	0	4
16S	Insecta	Lepidoptera	Geometridae	Ennominae	Ectropis	obliqua	NA	0.009	0	3	0
16S	Insecta	Lepidoptera	Tortricidae	Tortricinae			NA	0.003	0.0033	1	2
16S	Insecta	Lepidoptera	Geometridae	Ennominae	Jankowskia		NA	0.006	0	2	0
16S	Insecta	Lepidoptera	Geometridae	Larentiinae	Lobogonodes	erectaria	NA	0.003	0.0016	1	1
16S	Insecta	Lepidoptera	Erebidae	Lymantriinae	Lymantria		NA	0.006	0.0016	2	1
16S	Insecta	Lepidoptera	Geometridae	Ennominae	Myrioblephara	cilicornaria	NA	0.003	0	1	0
16S	Insecta	Lepidoptera					NA	0.003	0.0033	1	2
16S	Insecta	Lepidoptera	Noctuidae	Noctuinae	Panolis		NA	0.003	0	1	0
16S	Insecta	Lepidoptera	Noctuidae	Hadeninae	Panolis		NA	0.006	0	2	0
16S	Insecta	Lepidoptera	Noctuidae	Noctuinae	Panolis		NA	0.003	0.0016	1	1
16S	Insecta	Lepidoptera	Noctuidae				NA	0.003	0	1	0
16S	Insecta	Lepidoptera	Noctuidae				NA	0.003	0.0016	1	1
16S	Insecta	Lepidoptera	Thyrididae	Siculodinae	Pyrinioides	aurea	NA	0	0.0016	0	1
16S	Insecta	Lepidoptera	Saturniidae	Saturniinae	Saturnia		NA	0.016	0	5	0
16S	Insecta	Mecoptera	Panorpidae	Panorpinae	Panorpa	takenouchii	NA	0	0.0016	0	1
16S	Insecta	Neuroptera	Chrysopidae	Chrysopinae	Apertochrysa		NA	0	0.0016	0	1
16S	Insecta	Neuroptera	Chrysopidae	Chrysopinae	Chrysoperla		NA	0	0.0016	0	1
16S	Insecta	Neuroptera	Chrysopidae	Chrysopinae	Chrysoperla		NA	0	0.0016	0	1
16S	Insecta	Neuroptera	Osmylidae	Osmylinae	Osmylus		NA	0	0.0033	0	2
16S	Insecta	Neuroptera	Hemerobiidae	Hemerobiinae			NA	0	0.0049	0	3

Primer	Class	Order	Family	Subfamily	Genus	Species	BIN	M. ussuriensis	M. ikonnikovi	M. ussuriensis	M. ikonnikovi
16S	Insecta	Odonata	Libellulidae	Sympetrinae	Sympetrum	frequens	NA	0.016	0.0016	5	1
16S	Insecta	Odonata	Libellulidae	Sympetrinae	Sympetrum	infuscatum	NA	0.006	0	2	0
16S	Insecta	Orthoptera	Rhaphidophoridae	Aemodogryllinae			NA	0.003	0	1	0
16S	Insecta	Orthoptera	Trigonidiidae	Nemobiinae	Dianemobius	nigrofasciatus	NA	0.003	0	1	0
16S	Insecta	Orthoptera	Rhaphidophoridae	Aemodogryllinae	Diestrammena		NA	0.003	0	1	0
16S	Insecta	Orthoptera	Rhaphidophoridae				NA	0	0.0000	1	0
16S	Insecta	Orthoptera	Rhaphidophoridae				NA	0	0.0016	0	1
16S	Insecta	Plecoptera	Perlidae	Acroneuriinae	Calineuria		NA	0	0.0033	0	2
16S	Insecta	Plecoptera	Nemouridae				NA	0	0.0016	0	1
16S	Insecta	Psocodea	Amphipsocidae	Amphipsocinae	Amphipsocus	japonicus	NA	0	0.0016	0	1
16S	Insecta	Psocodea					NA	0	0.0016	0	1
16S	Insecta	Psocodea	Mesopsocidae	NA	Mesopsocus	unipunctatus	NA	0	0.0016	0	1
16S	Insecta	Psocodea	Psocidae	Psocinae	Psococerastis		NA	0.003	0.0049	1	3
16S	Insecta	Psocodea	Psocidae	Psocinae	Psococerastis	nubila	NA	0.016	0	5	0
16S	Insecta	Psocodea	Stenopsocidae	NA	Stenopsocus	nigricellus	NA	0	0.0033	0	2
16S	Insecta	Psocodea	Psocidae	Psocinae	Trichadenotecnum		NA	0	0.0016	0	1
16S	Insecta	Psocodea	Psocidae	Psocinae	Trichadenotecnum	incognitum	NA	0	0.0016	0	1
16S	Insecta	Psocodea	Psocidae	Psocinae	Trichadenotecnum		NA	0	0.0033	0	2
16S	Insecta	Thysanoptera	Thripidae	Thripinae	Thrips		NA	0	0.0033	0	2
