# Supplementary material for: Dietary analysis reveals differences in the prey use of two sympatric bat species
Source: Ecol Evol. 2021 Dec 16;11(24):18651–61. doi: 10.1002/ece3.8472 (PMC8717349; doi:10.1002/ece3.8472)
Supplement: Supplementary file 3 — Appendix S3 [file ECE3-11-18651-s003.pdf]

Supplementary information S3 for *Dietary analysis reveals differences in the prey use of two sympatric bat species*

Olga Heim[1], Anna I.E. Puisto[2], Ilari Sääksjärvi[3], Dai Fukui[4] and Eero J. Vesterinen[5]*

1 Faculty of Life and Medical Sciences, Doshisha University, 610-0321 Kyotanabe, Japan

2 Centre for Population Health Research, University of Turku, 20014 Turku, Finland

3 Biodiversity Unit, University of Turku, 20014 Turku, Finland

4 The University of Tokyo Hokkaido Forest, The University of Tokyo, 079-1563 Furano, Japan

5 Department of Biology, University of Turku, 20014 Turku, Finland

Prey item classification

Order	Family	Subfamily	Genus	Species	BIN	Flight capability	Reference	Diel activity	Reference	Mode of capture
Araneae	Lycosidae	NA	Pardosa		BOLD:ABA5238	non-volant	Morphology			gleaning
Araneae	Salticidae				BOLD:AAV1597	non-volant	Morphology			gleaning
Araneae	Salticidae				GBCH10991-13	non-volant	Morphology			gleaning
Araneae	Salticidae				BOLD:ACH5240	non-volant	Morphology			gleaning
Collembola	Bourletiellidae	NA	Deuterosminthurus		BOLD:AAB7915	non-volant	Morphology			gleaning
Blattodea	Ectobiidae	Ectobiinae			BOLD:ACI8100	non-volant	Morphology			gleaning
Coleoptera	Carabidae				BOLD:AAZ4621	non-volant	Roff 1990			gleaning
Coleoptera	Coccinellidae				BOLD:ACP4109	volant	Roff 1994	diurnal	Honek&Hodek 1996	gleaning
Coleoptera	Cantharidae	Cantharinae	Podabrus		BOLD:ACD6222	volant	Roff 1994	diurnal	Tsukiji 2020 (2021/05/18)	gleaning
Coleoptera	Cantharidae	Cantharinae	Podabrus		BOLD:ACD6257	volant	Roff 1994	diurnal	Tsukiji 2020 (2021/05/18)	gleaning
Coleoptera	Carabidae	Harpalinae			BOLD:ABX4019	non-volant	Roff 1990			gleaning
Coleoptera	Carabidae	Carabinae	Carabus		BOLD:ABY6003	non-volant	Roff 1990			gleaning
Coleoptera	Carabidae	Harpalinae	Pterostichus	oblongopunctatus	BOLD:ABY4764	non-volant	Roff 1990			gleaning
Coleoptera	Cerambycidae	Lamiinae	Monochamus		BOLD:AAC3388	volant	Roff 1994	undecided	Tsukiji 2020 (2021/05/18)	undecided
Coleoptera	Chrysomelidae	Chrysomelinae			BOLD:AAC0432	volant	Roff 1994	diurnal	Jolivet&Pierre 1981	gleaning
Coleoptera	Cryptophagidae	Atomariinae	Atomaria	turgida	BOLD:AAJ9463	volant	Roff 1994	nocturnal	Tharandt 2010	aerial-hawking
Coleoptera	Curculionidae	Entiminae	Polydrusus		BOLD:ACO8630	volant	Roff 1994	diurnal	Novella-Fernandez et al. 2020	gleaning
Coleoptera	Hydrophilidae	Hydrophilinae	Enochrus		BOLD:AAF0204	volant	Roff 1994	diurnal	Tsukiji 2020 (2021/05/18)	gleaning
Coleoptera	Nitidulidae	Nitidulinae	Cychramus	variegatus	BOLD:ABW4745	volant	Roff 1994	nocturnal	Yee & Kehl 2015; Mikkola 1972	aerial-hawking
Coleoptera	Staphylinidae	Euaesthetinae	Euaesthetus		BOLD:ACG9244	volant	Roff 1994	nocturnal		aerial-hawking
Diptera	Anthomyiidae				BOLD:AAG2490	volant	Roff 1990, 1994	diurnal	Novella-Fernandez et al. 2020	gleaning
Diptera	Tachinidae	Exoristinae			BOLD:AAI6325	volant	Roff 1990, 1994	diurnal	Novella-Fernandez et al. 2020	gleaning
Diptera	Tipulidae	Tipulinae	Tipula		BOLD:AAN5045	volant	Roff 1990, 1994	nocturnal	Mikkola 1972	aerial-hawking
Diptera	Tipulidae	Tipulinae	Tipula		BOLD:AAN9029	volant	Roff 1990, 1994	nocturnal	Mikkola 1972	aerial-hawking
Diptera	Limoniidae	Chioneinae	Teucholabis		BOLD:AAV2508	volant	Roff 1990, 1994	nocturnal	Novella-Fernandez et al. 2020	aerial-hawking
Diptera	Limoniidae	Chioneinae			BOLD:ABA4102	volant	Roff 1990, 1994	nocturnal	Novella-Fernandez et al. 2020	aerial-hawking
Diptera	Cecidomyiidae				BOLD:ABV2094	volant	Roff 1990, 1994	nocturnal	Scudder & Cannings 2006 (2021/05/19)	aerial-hawking
Diptera	Limoniidae				BOLD:ABX4369	volant	Roff 1990, 1994	nocturnal	Novella-Fernandez et al. 2020	aerial-hawking
Diptera	Limoniidae	Limoniinae	Libnotes		BOLD:ABY2385	volant	Roff 1990, 1994	nocturnal	Novella-Fernandez et al. 2020	aerial-hawking

Order	Family	Subfamily	Genus	Species	BIN	Flight capability	Reference	Diel activity	Reference	Mode of capture
Diptera	Syrphidae	Eristalinae			BOLD:ABY2389	volant	Roff 1990, 1994	diurnal	Novella-Fernandez et al. 2020	gleaning
Diptera	Tipulidae	Tipulinae	Tipula		BOLD:ABY5691	volant	Roff 1990, 1994	nocturnal	Mikkola 1972	aerial-hawking
Diptera	Phoridae				BOLD:ACA3150	volant	Roff 1990, 1994	diurnal	Novella-Fernandez et al. 2020	gleaning
Diptera	Tachinidae	Tachininae	Nemoraea	takanoi	BOLD:ACA7375	volant	Roff 1990, 1994	diurnal	Novella-Fernandez et al. 2020	gleaning
Diptera	Chironomidae	Chironominae	Glyptotendipes		BOLD:ACE3296	volant	Roff 1990, 1994	nocturnal	Scudder & Cannings 2006 (2021/05/19)	aerial-hawking
Diptera	Tachinidae				BOLD:ACF0578	volant	Roff 1990, 1994	diurnal	Novella-Fernandez et al. 2020	gleaning
Diptera	Tipulidae	Tipulinae	Tipula		BOLD:ACF0878	volant	Roff 1990, 1994	nocturnal	Mikkola 1972	aerial-hawking
Diptera	Phoridae				BOLD:ACF6080	volant	Roff 1990, 1994	diurnal	Novella-Fernandez et al. 2020	gleaning
Diptera	Sphaeroceridae	Limosininae	Rachispoda		BOLD:ACK2192	volant	Roff 1990, 1994	diurnal	Novella-Fernandez et al. 2020	gleaning
Diptera	Chironomidae				BOLD:ACK8680	volant	Roff 1990, 1994	nocturnal	Scudder & Cannings 2006 (2021/05/19)	aerial-hawking
Diptera	Sciaridae				BOLD:ACM6077	volant	Roff 1990, 1994	nocturnal	Novella-Fernandez et al. 2020	aerial-hawking
Diptera	Simuliidae				BOLD:ACM8222	volant	Roff 1990, 1994	nocturnal	Novella-Fernandez et al. 2020	aerial-hawking
Diptera	Limoniidae				BOLD:ACN0178	volant	Roff 1990, 1994	nocturnal	Novella-Fernandez et al. 2020	aerial-hawking
Diptera	Limoniidae				BOLD:ACN0238	volant	Roff 1990, 1994	nocturnal	Novella-Fernandez et al. 2020	aerial-hawking
Diptera	Mycetophilidae				BOLD:ACN0859	volant	Roff 1990, 1994	nocturnal	Mikkola 1972	aerial-hawking
Diptera	Calliphoridae				BOLD:ACN1515	volant	Roff 1990, 1994		Novella-Fernandez et al. 2020	gleaning
Diptera	Psychodidae				BOLD:ACN3276	volant	Roff 1990, 1994	nocturnal	Scudder & Cannings 2006 (2021/05/19)	aerial-hawking
Diptera	Drosophilidae				BOLD:ACN4805	volant	Roff 1990, 1994	diurnal	Novella-Fernandez et al. 2020	gleaning
Diptera					BOLD:ACN5240	volant	Roff 1990, 1994	undecided		undecided
Diptera	Limoniidae				BOLD:ACN5325	volant	Roff 1990, 1994	nocturnal	Novella-Fernandez et al. 2020	aerial-hawking
Diptera	Phoridae				BOLD:ACO0272	volant	Roff 1990, 1994	diurnal	Novella-Fernandez et al. 2020	gleaning
Diptera	Chironomidae				BOLD:ACO4488	volant	Roff 1990, 1994	nocturnal	Scudder & Cannings 2006 (2021/05/19)	aerial-hawking
Diptera					BOLD:ACP6542	volant	Roff 1990, 1994	undecided		undecided
Diptera	Chloropidae				BOLD:ACR0489	volant	Roff 1990, 1994	diurnal	Novella-Fernandez et al. 2020	gleaning
Diptera	Muscidae				BOLD:ACT7328	volant	Roff 1990, 1994	diurnal	Novella-Fernandez et al. 2020	gleaning
Diptera	Sciaridae				BOLD:ACU8386	volant	Roff 1990, 1994	nocturnal	Novella-Fernandez et al. 2020	aerial-hawking
Diptera	Muscidae	Phaoniinae	Helina		BOLD:ACV2533	volant	Roff 1990, 1994	diurnal	Novella-Fernandez et al. 2020	gleaning
Diptera	Psychodidae				BOLD:ACW2931	volant	Roff 1990, 1994	nocturnal	Scudder & Cannings 2006 (2021/05/19)	aerial-hawking
Diptera	Agromyzidae				BOLD:AAV4874	volant	Roff 1990, 1994	diurnal	Weintraub & Horowitz 1996	gleaning
Diptera	Anthomyiidae	Anthomyiinae	Botanophila		BOLD:AAG2502	volant	Roff 1990, 1994	diurnal	Novella-Fernandez et al. 2020	gleaning
Diptera	Anthomyiidae	Anthomyiinae	Lasiomma		BOLD:ACK1581	volant	Roff 1990, 1994	diurnal	Novella-Fernandez et al. 2020	gleaning
Diptera	Anthomyiidae	Anthomyiinae	Delia	platura	BOLD:AAG2511	volant	Roff 1990, 1994	diurnal	Novella-Fernandez et al. 2020	gleaning
Diptera	Anthomyiidae	Anthomyiinae	Botanophila	profuga	BOLD:ACP6334	volant	Roff 1990, 1994	diurnal	Novella-Fernandez et al. 2020	gleaning
Diptera	Bolitophilidae	NA	Bolitophila		BOLD:AAM9007	volant	Roff 1990, 1994	undecided		undecided
Diptera	Cecidomyiidae				BOLD:ACP9491	volant	Roff 1990, 1994	nocturnal	Scudder & Cannings 2006 (2021/05/19)	aerial-hawking
Diptera	Cecidomyiidae				BOLD:ACU9836	volant	Roff 1990, 1994	nocturnal	Scudder & Cannings 2006 (2021/05/19)	aerial-hawking
Diptera	Cecidomyiidae	Cecidomyiinae	Feltiella	acarisuga	BOLD:ACD4136	volant	Roff 1990, 1994	nocturnal	Scudder & Cannings 2006 (2021/05/19)	aerial-hawking
Diptera	Chamaemyiidae				BOLD:ACC5885	volant	Roff 1990, 1994		Novella-Fernandez et al. 2020	gleaning
Diptera	Chironomidae	Diamesinae	Diamesa		BOLD:AAB5106	volant	Roff 1990, 1994	nocturnal	Scudder & Cannings 2006 (2021/05/19)	aerial-hawking
Diptera	Chironomidae	Orthocladiinae	Heterotrissocladius	marcidus	BOLD:AAF2163	volant	Roff 1990, 1994	nocturnal	Scudder & Cannings 2006 (2021/05/19)	aerial-hawking
Diptera	Chironomidae	Orthocladiinae	Eukiefferiella		BOLD:AAI5126	volant	Roff 1990, 1994	nocturnal	Scudder & Cannings 2006 (2021/05/19)	aerial-hawking
Diptera					BOLD:ACQ9269	volant	Roff 1990, 1994	undecided		undecided
Diptera	Chironomidae	Orthocladiinae	Cricotopus	bicinctus	BOLD:AAT9677	volant	Roff 1990, 1994	nocturnal	Scudder & Cannings 2006 (2021/05/19)	aerial-hawking

Order	Family	Subfamily	Genus	Species	BIN	Flight capability	Reference	Diel activity	Reference	Mode of capture
Diptera	Chironomidae				BOLD:ACB8882	volant	Roff 1990, 1994	nocturnal	Scudder & Cannings 2006 (2021/05/19)	aerial-hawking
Diptera	Chironomidae				BOLD:ACF6427	volant	Roff 1990, 1994	nocturnal	Scudder & Cannings 2006 (2021/05/19)	aerial-hawking
Diptera	Chironomidae	Diamesinae			BOLD:ACK5496	volant	Roff 1990, 1994	nocturnal	Scudder & Cannings 2006 (2021/05/19)	aerial-hawking
Diptera	Chloropidae	Oscinellinae			BOLD:AAN5657	volant	Roff 1990, 1994	diurnal	Novella-Fernandez et al. 2020	gleaning
Diptera	Chloropidae				BOLD:ADC9928	volant	Roff 1990, 1994	diurnal	Novella-Fernandez et al. 2020	gleaning
Diptera	Culicidae	Culicinae			BOLD:AAF2904	volant	Roff 1990, 1994	nocturnal	Mikkola 1972	aerial-hawking
Diptera	Culicidae	Culicinae	Culex	orientalis	BOLD:ACB9306	volant	Roff 1990, 1994	nocturnal	Mikkola 1972	aerial-hawking
Diptera	Culicidae	Culicinae	Culex		BOLD:AAA4751	volant	Roff 1990, 1994	nocturnal	Mikkola 1972	aerial-hawking
Diptera	Cylindrotomidae	Cylindrotominae	Cylindrotoma		BOLD:AAD0770	volant	Roff 1990, 1994	undecided		undecided
Diptera	Drosophilidae	Drosophilinae	Drosophila		BOLD:AAY0127	volant	Roff 1990, 1994	diurnal	Novella-Fernandez et al. 2020	gleaning
Diptera	Drosophilidae	Drosophilinae	Drosophila		BOLD:ADH1432	volant	Roff 1990, 1994	diurnal	Novella-Fernandez et al. 2020	gleaning
Diptera	Drosophilidae	Steganinae	Leucophenga		BOLD:ACA1411	volant	Roff 1990, 1994	diurnal	Novella-Fernandez et al. 2020	gleaning
Diptera	Ephydridae				BOLD:ACS0447	volant	Roff 1990, 1994		Novella-Fernandez et al. 2020	gleaning
Diptera	Hybotidae	Tachydromiinae	Chersodromia		BOLD:ACJ9160	volant	Roff 1990, 1994		Novella-Fernandez et al. 2020	aerial-hawking
Diptera	Keroplatidae				BOLD:AAG4943	volant	Roff 1990, 1994		Novella-Fernandez et al. 2020	aerial-hawking
Diptera	Limoniidae	Limoniinae	Antocha	bifida	BOLD:AAW5955	volant	Roff 1990, 1994	nocturnal	Novella-Fernandez et al. 2020	aerial-hawking
Diptera	Limoniidae	Limoniinae	Dicranomyia	frontalis	BOLD:ABA5203	volant	Roff 1990, 1994	nocturnal	Novella-Fernandez et al. 2020	aerial-hawking
Diptera	Limoniidae	Limoniinae	Dicranomyia		BOLD:ABW0212	volant	Roff 1990, 1994	nocturnal	Novella-Fernandez et al. 2020	aerial-hawking
Diptera	Limoniidae				BOLD:ABV4791	volant	Roff 1990, 1994	nocturnal	Novella-Fernandez et al. 2020	aerial-hawking
Diptera	Limoniidae				BOLD:ACS9407	volant	Roff 1990, 1994	nocturnal	Novella-Fernandez et al. 2020	aerial-hawking
Diptera	Limoniidae	Limoniinae	Discobola		BOLD:ABW4922	volant	Roff 1990, 1994	nocturnal	Novella-Fernandez et al. 2020	aerial-hawking
Diptera	Limoniidae	Limoniinae	Libnotes		BOLD:ABW9478	volant	Roff 1990, 1994	nocturnal	Novella-Fernandez et al. 2020	aerial-hawking
Diptera	Limoniidae	Limoniinae	Dicranomyia		BOLD:AAO3939	volant	Roff 1990, 1994	nocturnal	Novella-Fernandez et al. 2020	aerial-hawking
Diptera	Limoniidae	Limoniinae	Dicranomyia		FINTI487-12	volant	Roff 1990, 1994	nocturnal	Novella-Fernandez et al. 2020	aerial-hawking
Diptera	Limoniidae	Limoniinae	Metalimnobia		BOLD:ABU9381	volant	Roff 1990, 1994	nocturnal	Novella-Fernandez et al. 2020	aerial-hawking
Diptera	Limoniidae	Limoniinae	Metalimnobia		BOLD:AAX5093	volant	Roff 1990, 1994	nocturnal	Novella-Fernandez et al. 2020	aerial-hawking
Diptera	Limoniidae	Limoniinae	Rhipidia	maculata	BOLD:ACB7799	volant	Roff 1990, 1994	nocturnal	Novella-Fernandez et al. 2020	aerial-hawking
Diptera	Lonchopteridae	NA	Lonchoptera		BOLD:ACB6694	volant	Roff 1990, 1994	nocturnal	Novella-Fernandez et al. 2020	aerial-hawking
Diptera	Muscidae				BOLD:AAG1765	volant	Roff 1990, 1994	diurnal	Novella-Fernandez et al. 2020	gleaning
Diptera	Muscidae				BOLD:ACD2056	volant	Roff 1990, 1994	diurnal	Novella-Fernandez et al. 2020	gleaning
Diptera	Muscidae	Phaoniinae	Helina	obscurata	BOLD:AAE6931	volant	Roff 1990, 1994	diurnal	Novella-Fernandez et al. 2020	gleaning
Diptera	Muscidae	Azeliinae	Muscina	pascuorum	BOLD:AAG1714	volant	Roff 1990, 1994	diurnal	Novella-Fernandez et al. 2020	gleaning
Diptera	Muscidae				BOLD:ACR5608	volant	Roff 1990, 1994	diurnal	Novella-Fernandez et al. 2020	gleaning
Diptera	Muscidae				BOLD:AAX1493	volant	Roff 1990, 1994	diurnal	Novella-Fernandez et al. 2020	gleaning
Diptera	Muscidae	Azeliinae	Hydrotaea	armipes	BOLD:AAG6908	volant	Roff 1990, 1994	diurnal	Novella-Fernandez et al. 2020	gleaning
Diptera	Muscidae	Phaoniinae	Phaonia		BOLD:AAG1772	volant	Roff 1990, 1994	diurnal	Novella-Fernandez et al. 2020	gleaning
Diptera	Muscidae	Phaoniinae	Phaonia		BOLD:AAP8142	volant	Roff 1990, 1994	diurnal	Novella-Fernandez et al. 2020	gleaning
Diptera	Muscidae	Azeliinae	Hydrotaea	glabricula	BOLD:ADK1195	volant	Roff 1990, 1994	diurnal	Novella-Fernandez et al. 2020	gleaning
Diptera	Muscidae	Azeliinae	Potamia	littoralis	BOLD:ACQ0523	volant	Roff 1990, 1994	diurnal	Novella-Fernandez et al. 2020	gleaning
Diptera	Muscidae	Azeliinae	Thricops	diaphanus	BOLD:AAG1710	volant	Roff 1990, 1994	diurnal	Novella-Fernandez et al. 2020	gleaning
Diptera	Mycetophilidae	Mycetophilinae	Platurocypta		BOLD:ACC5656	volant	Roff 1990, 1994	nocturnal	Mikkola 1972	aerial-hawking
Diptera	Pediciidae	Uliinae	Ula		BOLD:AAV1814	volant	Roff 1990, 1994	nocturnal	Novella-Fernandez et al. 2020	aerial-hawking
Diptera	Pediciidae	Pediciinae	Dicranota		BOLD:ABW5265	volant	Roff 1990, 1994	nocturnal	Novella-Fernandez et al. 2020	aerial-hawking

Order	Family	Subfamily	Genus	Species	BIN	Flight capability	Reference	Diel activity	Reference	Mode of capture
Diptera	Pediciidae	Uliinae	Ula	bolitophila	BOLD:ABU5946	volant	Roff 1990, 1994	nocturnal	Novella-Fernandez et al. 2020	aerial-hawking
Diptera	Pediciidae	Uliinae	Ula		BOLD:ABA7462	volant	Roff 1990, 1994	nocturnal	Novella-Fernandez et al. 2020	aerial-hawking
Diptera	Periscelididae				BOLD:ADE5755	volant	Roff 1990, 1994	undecided		undecided
Diptera	Phoridae				BOLD:AAP6413	volant	Roff 1990, 1994	diurnal	Novella-Fernandez et al. 2020	gleaning
Diptera	Psychodidae				BOLD:ACO7340	volant	Roff 1990, 1994	nocturnal	Scudder & Cannings 2006 (2021/05/19)	aerial-hawking
Diptera	Psychodidae				BOLD:ADE7332	volant	Roff 1990, 1994	nocturnal	Scudder & Cannings 2006 (2021/05/19)	aerial-hawking
Diptera	Psychodidae				BOLD:ADE8063	volant	Roff 1990, 1994	nocturnal	Scudder & Cannings 2006 (2021/05/19)	aerial-hawking
Diptera	Psychodidae	Psychodinae	Psychoda		BOLD:AAL7819	volant	Roff 1990, 1994	nocturnal	Scudder & Cannings 2006 (2021/05/19)	aerial-hawking
Diptera	Psychodidae	Psychodinae	Psychoda		BOLD:ACD9559	volant	Roff 1990, 1994	nocturnal	Scudder & Cannings 2006 (2021/05/19)	aerial-hawking
Diptera	Sarcophagidae				BOLD:ACU3079	volant	Roff 1990, 1994	diurnal	Novella-Fernandez et al. 2020	gleaning
Diptera	Sciaridae				BOLD:ACC1760	volant	Roff 1990, 1994	nocturnal	Novella-Fernandez et al. 2020	aerial-hawking
Diptera	Sciaridae				BOLD:ACI7364	volant	Roff 1990, 1994	nocturnal	Novella-Fernandez et al. 2020	aerial-hawking
Diptera	Simuliidae	Simuliinae	Simulium		BOLD:ABA1846	volant	Roff 1990, 1994	nocturnal	Novella-Fernandez et al. 2020	aerial-hawking
Diptera	Sphaeroceridae	Limosininae	Opalimosina	mirabilis	BOLD:AAN6406	volant	Roff 1990, 1994	diurnal	Novella-Fernandez et al. 2020	gleaning
Diptera	Syrphidae	Eristalinae	Ferdinandea	cuprea	BOLD:AAJ0402	volant	Roff 1990, 1994	diurnal	Novella-Fernandez et al. 2020	gleaning
Diptera	Tachinidae	Exoristinae	Cyzenis		BOLD:AAP4829	volant	Roff 1990, 1994	diurnal	Novella-Fernandez et al. 2020	gleaning
Diptera	Tachinidae	Exoristinae	Drino		BOLD:AAB6198	volant	Roff 1990, 1994	diurnal	Novella-Fernandez et al. 2020	gleaning
Diptera	Tachinidae	Exoristinae			BOLD:AAN9266	volant	Roff 1990, 1994	diurnal	Novella-Fernandez et al. 2020	gleaning
Diptera	Tachinidae	Exoristinae	Cadurciella	tritaeniata	BOLD:AAN9651	volant	Roff 1990, 1994	diurnal	Novella-Fernandez et al. 2020	gleaning
Diptera	Tachinidae	Exoristinae	Phebellia	villica	BOLD:ACB0760	volant	Roff 1990, 1994	diurnal	Novella-Fernandez et al. 2020	gleaning
Diptera	Tachinidae				BOLD:AAZ8670	volant	Roff 1990, 1994	diurnal	Novella-Fernandez et al. 2020	gleaning
Diptera	Tachinidae	Exoristinae			BOLD:AAC1692	volant	Roff 1990, 1994	diurnal	Novella-Fernandez et al. 2020	gleaning
Diptera	Tipulidae	Tipulinae	Tipula	kuzuensis	BOLD:AAN9034	volant	Roff 1990, 1994	nocturnal	Mikkola 1972	aerial-hawking
Diptera	Tipulidae	Tipulinae	Tipula		BOLD:ABV3695	volant	Roff 1990, 1994	nocturnal	Mikkola 1972	aerial-hawking
Diptera	Tipulidae	Tipulinae	Tipula		BOLD:ABV4667	volant	Roff 1990, 1994	nocturnal	Mikkola 1972	aerial-hawking
Diptera	Tipulidae	Tipulinae	Tipula		TIPTW088-10	volant	Roff 1990, 1994	nocturnal	Mikkola 1972	aerial-hawking
Diptera	Tipulidae				TIPTW093-10	volant	Roff 1990, 1994	nocturnal	Mikkola 1972	aerial-hawking
Diptera	Trichoceridae	Trichocerinae	Trichocera		BOLD:ACF7745	volant	Roff 1990, 1994	diurnal	Scudder & Cannings 2006 (2021/05/19)	gleaning
Hemiptera	Pentatomidae				BOLD:AAP3528	volant	Roff 1994	diurnal	Capinera 2008	gleaning
Hemiptera	Cicadellidae	Deltocephalinae			BOLD:AAG8956	volant	Roff 1994	diurnal	Capinera 2008	gleaning
Hemiptera	Cicadidae	Cicadinae	Yezoterpnosia	nigricosta	BOLD:ACP9813	volant	Roff 1990	diurnal	Capinera 2008	gleaning
Hemiptera	Psyllidae				BOLD:ACM6872	volant	Roff 1994	diurnal	Capinera 2008	gleaning
Hymenoptera	Cimbicidae	Cimbicinae	Trichiosoma		BOLD:ACJ7002	volant	Roff 1990	diurnal		gleaning
Hymenoptera	Cimbicidae	Cimbicinae	Trichiosoma		BOLD:ABZ2163	volant	Roff 1990	diurnal		gleaning
Hymenoptera	Ichneumonidae				BOLD:ADI1279	volant	Roff 1990	nocturnal	Mikkola 1972	aerial-hawking
Hymenoptera	Pamphiliidae	Pamphiliinae	Neurotoma	nemoralis	BOLD:AAV6346	volant	Roff 1990	diurnal		gleaning
Hymenoptera	Pamphiliidae	Pamphiliinae	Pamphilius		BOLD:AAK4614	volant	Roff 1990	diurnal		gleaning
Hymenoptera					EII067-15	volant	Roff 1990	undecided		undecided
Hymenoptera					EII104-15	volant	Roff 1990	undecided		undecided
Hymenoptera	Tenthredinidae	Nematinae	Pristiphora		BOLD:AAK9450	volant	Roff 1990	diurnal	Novella-Fernandez et al. 2020	gleaning
Lepidoptera	Coleophoridae	Coleophorinae	Coleophora		BOLD:AAC8694	volant	Roff 1994	diurnal	Capinera 2008	gleaning
Lepidoptera	Crambidae	Scopariinae	Eudonia	persimilis	BOLD:AAF1478	volant	Roff 1994	nocturnal	Capinera 2008	aerial-hawking
Lepidoptera					BOLD:AAF8577	volant	Roff 1994	undecided		undecided

Order	Family	Subfamily	Genus	Species	BIN	Flight capability	Reference	Diel activity	Reference	Mode of capture
Lepidoptera					BOLD:AAG6034	volant	Roff 1994	undecided		undecided
Lepidoptera	Erebidae	Arctiinae			BOLD:AAH2626	volant	Roff 1994	diurnal	Kawahara et al. 2017	gleaning
Lepidoptera	Gelechiidae	Gelechiinae			BOLD:AAH5654	volant	Roff 1994	nocturnal	Kawahara et al. 2017	aerial-hawking
Lepidoptera	Noctuidae	Noctuinae			BOLD:AAI3519	volant	Roff 1994	nocturnal	Capinera 2008	aerial-hawking
Lepidoptera	Erebidae				BOLD:AAL4384	volant	Roff 1994	nocturnal	Kawahara et al. 2017	aerial-hawking
Lepidoptera	Crambidae	Spilomelinae	Pleuroptya	expictalis	BOLD:AAL4502	volant	Roff 1994	nocturnal	Capinera 2008	aerial-hawking
Lepidoptera	Erebidae	Boletobiinae	Enispa		BOLD:AAL7095	volant	Roff 1994	nocturnal	Kawahara et al. 2017	aerial-hawking
Lepidoptera	Noctuidae	Noctuinae			BOLD:AAM0480	volant	Roff 1994	nocturnal	Capinera 2008	aerial-hawking
Lepidoptera	Notodontidae	Notodontinae	Cerura		BOLD:AAM4552	volant	Roff 1994	nocturnal	Capinera 2008	aerial-hawking
Lepidoptera	Noctuidae				BOLD:AAM9055	volant	Roff 1994	nocturnal	Capinera 2008	aerial-hawking
Lepidoptera	Noctuidae	Noctuinae			BOLD:AAM9761	volant	Roff 1994	nocturnal	Capinera 2008	aerial-hawking
Lepidoptera	Crambidae	Pyraustinae	Goniorhynchus		BOLD:AAN3555	volant	Roff 1994	nocturnal	Capinera 2008	aerial-hawking
Lepidoptera	Hesperiidae				BOLD:AAN7248	volant	Roff 1994	diurnal	Capinera 2008	gleaning
Lepidoptera	Noctuidae				BOLD:AAP1728	volant	Roff 1994	nocturnal	Capinera 2008	aerial-hawking
Lepidoptera	Geometridae	Ennominae			BOLD:AAP2372	volant	Roff 1994	nocturnal	Capinera 2008	aerial-hawking
Lepidoptera	Gelechiidae				BOLD:AAQ1460	volant	Roff 1994	nocturnal	Kawahara et al. 2017	aerial-hawking
Lepidoptera	Crambidae	Spilomelinae			BOLD:AAQ2087	volant	Roff 1994	nocturnal	Capinera 2008	aerial-hawking
Lepidoptera	Geometridae	Sterrhinae	Scopula		BOLD:AAV8961	volant	Roff 1994	nocturnal	Capinera 2008	aerial-hawking
Lepidoptera	Erebidae	Arctiinae			BOLD:AAY5858	volant	Roff 1994	diurnal	Kawahara et al. 2017	gleaning
Lepidoptera	Limacodidae				BOLD:AAZ8422	volant	Roff 1994	nocturnal	Capinera 2008	aerial-hawking
Lepidoptera	Tortricidae	Olethreutinae	Rhopobota		BOLD:ABA8634	volant	Roff 1994	nocturnal	Kawahara et al. 2017	aerial-hawking
Lepidoptera	Geometridae	Larentiinae	Leptostegna		BOLD:ABU5788	volant	Roff 1994	nocturnal	Capinera 2008	aerial-hawking
Lepidoptera	Sphingidae	Smerinthinae	Marumba	gaschkewitschii	BOLD:ABU7231	volant	Roff 1994	nocturnal	Capinera 2008	aerial-hawking
Lepidoptera					BOLD:ABU9226	volant	Roff 1994	undecided		undecided
Lepidoptera	Tortricidae				BOLD:ABW0658	volant	Roff 1994	nocturnal	Kawahara et al. 2017	aerial-hawking
Lepidoptera	Erebidae	Arctiinae	Cyana		BOLD:ABX3752	volant	Roff 1994	diurnal	Kawahara et al. 2017	gleaning
Lepidoptera	Erebidae	Erebinae	Catocala		BOLD:ABZ3190	volant	Roff 1994	nocturnal	Kawahara et al. 2017	aerial-hawking
Lepidoptera	Erebidae	Arctiinae			BOLD:ACB1726	volant	Roff 1994	diurnal	Kawahara et al. 2017	gleaning
Lepidoptera	Geometridae	Geometrinae			BOLD:ACE9755	volant	Roff 1994	nocturnal	Capinera 2008	aerial-hawking
Lepidoptera	Autostichidae				BOLD:ACF7812	volant	Roff 1994	nocturnal	Kawahara et al. 2017	aerial-hawking
Lepidoptera	Cosmopterigidae				BOLD:ACJ1842	volant	Roff 1994	diurnal	Capinera 2008	gleaning
Lepidoptera	Gelechiidae	Gelechiinae	Sophronia		BOLD:ACL1953	volant	Roff 1994	nocturnal	Kawahara et al. 2017	aerial-hawking
Lepidoptera	Geometridae	Ennominae			BOLD:ACL6993	volant	Roff 1994	nocturnal	Capinera 2008	aerial-hawking
Lepidoptera					BOLD:ACM3459	volant	Roff 1994	undecided		undecided
Lepidoptera	Elachistidae				BOLD:ACM9615	volant	Roff 1994	nocturnal	Capinera 2008	aerial-hawking
Lepidoptera	Tortricidae				BOLD:ACN0142	volant	Roff 1994	nocturnal	Kawahara et al. 2017	aerial-hawking
Lepidoptera	Gelechiidae				BOLD:ACN0185	volant	Roff 1994	nocturnal	Kawahara et al. 2017	aerial-hawking
Lepidoptera	Tortricidae	Tortricinae	Choristoneura		BOLD:ACN0625	volant	Roff 1994	nocturnal	Kawahara et al. 2017	aerial-hawking
Lepidoptera	Erebidae	Herminiinae			BOLD:ACO1434	volant	Roff 1994	nocturnal	Kawahara et al. 2017	aerial-hawking
Lepidoptera	Lasiocampidae	Poecilocampinae			BOLD:ACP2255	volant	Roff 1994	nocturnal	Capinera 2008	aerial-hawking
Lepidoptera	Gelechiidae				BOLD:ACR6168	volant	Roff 1994	nocturnal	Kawahara et al. 2017	aerial-hawking
Lepidoptera	Stathmopodidae	NA	Stathmopoda		BOLD:ACV9615	volant	Roff 1994	diurnal	Kawahara et al. 2017	gleaning
Lepidoptera					BOLD:ACW1375	volant	Roff 1994	undecided		undecided

Order	Family	Subfamily	Genus	Species	BIN	Flight capability	Reference	Diel activity	Reference	Mode of capture
Lepidoptera					BOLD:ACX5590	volant	Roff 1994	undecided		undecided
Lepidoptera	Crambidae				BOLD:ACX6648	volant	Roff 1994	nocturnal	Capinera 2008	aerial-hawking
Lepidoptera					BOLD:ADF8756	volant	Roff 1994	undecided		undecided
Lepidoptera					BOLD:ADG3051	volant	Roff 1994	undecided		undecided
Lepidoptera					BOLD:ADI1550	volant	Roff 1994	undecided		undecided
Lepidoptera	Tortricidae	Tortricinae	Acleris	paradiseana	BOLD:ADI2826	volant	Roff 1994	nocturnal	Kawahara et al. 2017	aerial-hawking
Lepidoptera					BOLD:ADI2961	volant	Roff 1994	undecided		undecided
Lepidoptera					BOLD:ADI4331	volant	Roff 1994	undecided		undecided
Lepidoptera	Geometridae	Sterrhinae	Scopula		BOLD:ADI7455	volant	Roff 1994	nocturnal	Capinera 2008	aerial-hawking
Lepidoptera	Geometridae	Sterrhinae	Scopula		BOLD:ADJ2221	volant	Roff 1994	nocturnal	Capinera 2008	aerial-hawking
Lepidoptera					GWOSS664-11	volant	Roff 1994	undecided		undecided
Lepidoptera	Geometridae	Geometrinae			NAGEO164-09	volant	Roff 1994	nocturnal	Capinera 2008	aerial-hawking
Lepidoptera	Autostichidae	Autostichinae			BOLD:AAC7842	volant	Roff 1994	nocturnal	Kawahara et al. 2017	aerial-hawking
Lepidoptera	Blastobasidae	Blastobasinae	Hypatopa		BOLD:AAB9632	volant	Roff 1994	nocturnal	Capinera 2008	aerial-hawking
Lepidoptera	Blastobasidae	Blastobasinae			BOLD:AAX6193	volant	Roff 1994	nocturnal	Capinera 2008	aerial-hawking
Lepidoptera	Coleophoridae	Coleophorinae	Coleophora		BOLD:AAB8487	volant	Roff 1994	diurnal	Capinera 2008	gleaning
Lepidoptera	Crambidae	Pyraustinae	Pyrausta		BOLD:AAD0032	volant	Roff 1994	nocturnal	Capinera 2008	aerial-hawking
Lepidoptera					BOLD:ACM1938	volant	Roff 1994	undecided		undecided
Lepidoptera	Crambidae	Acentropinae	Nymphula		BOLD:AAF2874	volant	Roff 1994	nocturnal	Capinera 2008	aerial-hawking
Lepidoptera	Crambidae	Spilomelinae	Palpita		BOLD:AAD6468	volant	Roff 1994	nocturnal	Capinera 2008	aerial-hawking
Lepidoptera	Crambidae	Spilomelinae			BOLD:AAB6255	volant	Roff 1994	nocturnal	Capinera 2008	aerial-hawking
Lepidoptera	Crambidae	Spilomelinae	Pleuroptya		BOLD:AAB6257	volant	Roff 1994	nocturnal	Capinera 2008	aerial-hawking
Lepidoptera	Depressariidae				BOLD:AAY5033	volant	Roff 1994	nocturnal	Kawahara et al. 2017	aerial-hawking
Lepidoptera	Depressariidae	Depressariinae	Agonopterix		BOLD:AAF7176	volant	Roff 1994	nocturnal	Kawahara et al. 2017	aerial-hawking
Lepidoptera	Depressariidae	Depressariinae	Depressaria		BOLD:ACN1500	volant	Roff 1994	nocturnal	Kawahara et al. 2017	aerial-hawking
Lepidoptera	Depressariidae				BOLD:ACE7937	volant	Roff 1994	nocturnal	Kawahara et al. 2017	aerial-hawking
Lepidoptera	Drepanidae	Thyatirinae	Tetheella	fluctuosa	BOLD:AAD3647	volant	Roff 1994	nocturnal	Capinera 2008	aerial-hawking
Lepidoptera	Erebidae				BOLD:AAB7538	volant	Roff 1994	nocturnal	Kawahara et al. 2017	aerial-hawking
Lepidoptera	Erebidae	Erebinae	Catocala		BOLD:AAE9541	volant	Roff 1994	nocturnal	Kawahara et al. 2017	aerial-hawking
Lepidoptera	Erebidae	Arctiinae			BOLD:AAC3572	volant	Roff 1994	diurnal	Kawahara et al. 2017	gleaning
Lepidoptera	Erebidae	Hypeninae			BOLD:AAC7130	volant	Roff 1994	nocturnal	Kawahara et al. 2017	aerial-hawking
Lepidoptera	Erebidae	Erebinae	Ercheia		BOLD:AAD8285	volant	Roff 1994	nocturnal	Kawahara et al. 2017	aerial-hawking
Lepidoptera	Erebidae	Erebinae	Thyas	juno	BOLD:AAF1405	volant	Roff 1994	nocturnal	Kawahara et al. 2017	aerial-hawking
Lepidoptera	Erebidae	Erebinae			BOLD:ABX5513	volant	Roff 1994	nocturnal	Kawahara et al. 2017	aerial-hawking
Lepidoptera	Erebidae	Arctiinae			BOLD:ACE8889	volant	Roff 1994	diurnal	Kawahara et al. 2017	gleaning
Lepidoptera	Erebidae				BOLD:ACT9815	volant	Roff 1994	nocturnal	Kawahara et al. 2017	aerial-hawking
Lepidoptera	Erebidae				BOLD:ADF0678	volant	Roff 1994	nocturnal	Kawahara et al. 2017	aerial-hawking
Lepidoptera	Erebidae	Herminiinae	Hydrillodes		BOLD:ACU0270	volant	Roff 1994	nocturnal	Kawahara et al. 2017	aerial-hawking
Lepidoptera					BOLD:AAV8834	volant	Roff 1994	undecided		undecided
Lepidoptera	Erebidae	Lymantriinae	Lymantria	dispar	BOLD:AAA2052	volant	Roff 1994	diurnal	Tsukiji 2020 (2021/05/18)	gleaning
Lepidoptera	Erebidae	Lymantriinae	Lymantria	monacha	BOLD:AAA5537	volant	Roff 1994	nocturnal	Tsukiji 2020 (2021/05/18)	aerial-hawking
Lepidoptera	Erebidae	Pangraptinae	Pangrapta		BOLD:ADF1765	volant	Roff 1994	nocturnal	Kawahara et al. 2017	aerial-hawking
Lepidoptera	Gelechiidae	Gelechiinae	Gelechia	cuneatella	BOLD:AAF5086	volant	Roff 1994	nocturnal	Kawahara et al. 2017	aerial-hawking

Order	Family	Subfamily	Genus	Species	BIN	Flight capability	Reference	Diel activity	Reference	Mode of capture
Lepidoptera	Gelechiidae	Gelechiinae	Psoricoptera	gibbosella	BOLD:AAD0608	volant	Roff 1994	nocturnal	Kawahara et al. 2017	aerial-hawking
Lepidoptera	Gelechiidae				BOLD:ABA1140	volant	Roff 1994	nocturnal	Kawahara et al. 2017	aerial-hawking
Lepidoptera	Geometridae	Ennominae			BOLD:ACG8719	volant	Roff 1994	nocturnal	Capinera 2008	aerial-hawking
Lepidoptera	Geometridae	Ennominae	Cleora	insolita	BOLD:ACJ3813	volant	Roff 1994	nocturnal	Capinera 2008	aerial-hawking
Lepidoptera	Geometridae	Ennominae			BOLD:AAC6655	volant	Roff 1994	nocturnal	Capinera 2008	aerial-hawking
Lepidoptera	Geometridae	Ennominae	Deileptenia	ribeata	BOLD:AAC3800	volant	Roff 1994	nocturnal	Capinera 2008	aerial-hawking
Lepidoptera	Geometridae	Ennominae	Ectropis		BOLD:AAB5227	volant	Roff 1994	nocturnal	Capinera 2008	aerial-hawking
Lepidoptera	Geometridae	Ennominae	Ectropis	crepuscularia	BOLD:AAA2076	volant	Roff 1994	nocturnal	Capinera 2008	aerial-hawking
Lepidoptera	Geometridae	Ennominae	Endropiodes		BOLD:AAF7479	volant	Roff 1994	nocturnal	Capinera 2008	aerial-hawking
Lepidoptera	Geometridae	Larentiinae	Gandaritis	fixseni	BOLD:AAY1335	volant	Roff 1994	nocturnal	Capinera 2008	aerial-hawking
Lepidoptera	Geometridae	Larentiinae	Gandaritis		BOLD:AAB5983	volant	Roff 1994	nocturnal	Capinera 2008	aerial-hawking
Lepidoptera	Geometridae	Geometrinae	Geometra		BOLD:AAB2011	volant	Roff 1994	nocturnal	Capinera 2008	aerial-hawking
Lepidoptera	Geometridae	Ennominae	Protoboarmia		BOLD:AAA2077	volant	Roff 1994	nocturnal	Capinera 2008	aerial-hawking
Lepidoptera	Geometridae	Larentiinae	Eupithecia		BOLD:AAA2083	volant	Roff 1994	nocturnal	Capinera 2008	aerial-hawking
Lepidoptera	Geometridae	Sterrhinae	Cyclophora		BOLD:AAD6021	volant	Roff 1994	nocturnal	Capinera 2008	aerial-hawking
Lepidoptera	Geometridae	Geometrinae			BOLD:AAE5087	volant	Roff 1994	nocturnal	Capinera 2008	aerial-hawking
Lepidoptera	Geometridae	Ennominae			BOLD:AAE6522	volant	Roff 1994	nocturnal	Capinera 2008	aerial-hawking
Lepidoptera	Geometridae	Ennominae	Menophra	senilis	BOLD:AAF3823	volant	Roff 1994	nocturnal	Capinera 2008	aerial-hawking
Lepidoptera	Geometridae	Ennominae			BOLD:AAL8521	volant	Roff 1994	nocturnal	Capinera 2008	aerial-hawking
Lepidoptera	Geometridae	Ennominae			BOLD:AAP9222	volant	Roff 1994	nocturnal	Capinera 2008	aerial-hawking
Lepidoptera	Geometridae				BOLD:AAZ1901	volant	Roff 1994	nocturnal	Capinera 2008	aerial-hawking
Lepidoptera	Geometridae	Ennominae			BOLD:ABU6285	volant	Roff 1994	nocturnal	Capinera 2008	aerial-hawking
Lepidoptera	Geometridae	Geometrinae	Jodis	putata	BOLD:ABZ4040	volant	Roff 1994	nocturnal	Capinera 2008	aerial-hawking
Lepidoptera	Geometridae	Larentiinae			BOLD:ACB9627	volant	Roff 1994	nocturnal	Capinera 2008	aerial-hawking
Lepidoptera	Geometridae				BOLD:ADF1852	volant	Roff 1994	nocturnal	Capinera 2008	aerial-hawking
Lepidoptera	Geometridae	Sterrhinae	Scopula		LNAUS1341-13	volant	Roff 1994	nocturnal	Capinera 2008	aerial-hawking
Lepidoptera	Geometridae	Ennominae	Hypomecis	punctinalis	BOLD:ACA2461	volant	Roff 1994	nocturnal	Capinera 2008	aerial-hawking
Lepidoptera	Geometridae	Ennominae			BOLD:ACS9064	volant	Roff 1994	nocturnal	Capinera 2008	aerial-hawking
Lepidoptera	Geometridae	Ennominae			BOLD:AAB6560	volant	Roff 1994	nocturnal	Capinera 2008	aerial-hawking
Lepidoptera	Geometridae	Ennominae	Ourapteryx	maculicaudaria	BOLD:AAW9443	volant	Roff 1994	nocturnal	Capinera 2008	aerial-hawking
Lepidoptera	Geometridae	Ennominae			BOLD:AAA2521	volant	Roff 1994	nocturnal	Capinera 2008	aerial-hawking
Lepidoptera	Geometridae	Ennominae	Phthonosema	tendinosaria	BOLD:AAF6489	volant	Roff 1994	nocturnal	Capinera 2008	aerial-hawking
Lepidoptera	Gracillariidae	Gracillariinae	Caloptilia	cf. heringi	BOLD:ADK1669	volant	Roff 1994	nocturnal	Tsukiji 2020 (2021/05/18)	aerial-hawking
Lepidoptera	Gracillariidae	Gracillariinae	Caloptilia	hidakensis	BOLD:AAK1674	volant	Roff 1994	nocturnal	Tsukiji 2020 (2021/05/18)	aerial-hawking
Lepidoptera	Gracillariidae	Gracillariinae	Caloptilia		BOLD:ACL6329	volant	Roff 1994	nocturnal	Tsukiji 2020 (2021/05/18)	aerial-hawking
Lepidoptera	Gracillariidae	Gracillariinae			BOLD:ADK2510	volant	Roff 1994	diurnal	Capinera 2008	gleaning
Lepidoptera	Hepialidae	Hepialinae			BOLD:AAO2948	volant	Roff 1994	nocturnal	Capinera 2008	aerial-hawking
Lepidoptera	Hepialidae	Hepialinae			BOLD:ACN8703	volant	Roff 1994	nocturnal	Capinera 2008	aerial-hawking
Lepidoptera	Hesperiidae	Coeliadinae	Burara	aquilina	BOLD:ACD6545	volant	Roff 1994	diurnal	Capinera 2008	gleaning
Lepidoptera	Hesperiidae	Hesperiinae			LIMBC545-11	volant	Roff 1994	diurnal	Capinera 2008	gleaning
Lepidoptera	Hesperiidae	Hesperiinae	Thoressa	varia	BOLD:ADK0840	volant	Roff 1994	diurnal	Capinera 2008	gleaning
Lepidoptera	Lasiocampidae	Lasiocampinae	Euthrix	potatoria	BOLD:AAC1584	volant	Roff 1994	nocturnal	Capinera 2008	aerial-hawking
Lepidoptera	Lecithoceridae				BOLD:AAD6680	volant	Roff 1994	nocturnal	Kawahara et al. 2017	aerial-hawking

Order	Family	Subfamily	Genus	Species	BIN	Flight capability	Reference	Diel activity	Reference	Mode of capture
Lepidoptera	Limacodidae	Limacodinae			BOLD:ACE6221	volant	Roff 1994	nocturnal	Capinera 2008	aerial-hawking
Lepidoptera	Limacodidae	Limacodinae			BOLD:AAA9203	volant	Roff 1994	nocturnal	Capinera 2008	aerial-hawking
Lepidoptera	Limacodidae				BOLD:ACC2517	volant	Roff 1994	nocturnal	Capinera 2008	aerial-hawking
Lepidoptera	Limacodidae	Limacodinae	Parasa		BOLD:AAM0823	volant	Roff 1994	nocturnal	Capinera 2008	aerial-hawking
Lepidoptera	Limacodidae	Limacodinae	Phrixolepia		BOLD:AAV5028	volant	Roff 1994	nocturnal	Capinera 2008	aerial-hawking
Lepidoptera	Lycaenidae	Theclinae	Rapala		BOLD:AAL1375	volant	Roff 1994	diurnal	Tsukiji 2020 (2021/05/18)	gleaning
Lepidoptera	Noctuidae	Acronictinae	Acronicta		BOLD:ADK0044	volant	Roff 1994	nocturnal	Capinera 2008	aerial-hawking
Lepidoptera	Noctuidae	Pantheinae	Anacronicta		BOLD:AAK1458	volant	Roff 1994	nocturnal	Capinera 2008	aerial-hawking
Lepidoptera	Noctuidae	Noctuinae	Apamea	helva	BOLD:AAC5412	volant	Roff 1994	nocturnal	Capinera 2008	aerial-hawking
Lepidoptera	Noctuidae	Noctuinae	Athetis		BOLD:AAV0425	volant	Roff 1994	nocturnal	Capinera 2008	aerial-hawking
Lepidoptera	Noctuidae	Acronictinae	Belciades	niveola	BOLD:ACU0724	volant	Roff 1994	nocturnal	Capinera 2008	aerial-hawking
Lepidoptera	Noctuidae	Amphipyrinae	Brachionycha	nubeculosa	BOLD:AAE0860	volant	Roff 1994	nocturnal	Capinera 2008	aerial-hawking
Lepidoptera					BOLD:ADF8757	volant	Roff 1994	undecided		undecided
Lepidoptera	Noctuidae				BOLD:ACT9151	volant	Roff 1994	nocturnal	Capinera 2008	aerial-hawking
Lepidoptera	Noctuidae	Noctuinae			BOLD:ACJ1078	volant	Roff 1994	nocturnal	Capinera 2008	aerial-hawking
Lepidoptera	Noctuidae	Oncocnemidinae			BOLD:AAE4319	volant	Roff 1994	nocturnal	Capinera 2008	aerial-hawking
Lepidoptera	Noctuidae	Plusiinae			BOLD:AAE7504	volant	Roff 1994	nocturnal	Capinera 2008	aerial-hawking
Lepidoptera	Noctuidae	Amphipyrinae			BOLD:AAW4866	volant	Roff 1994	nocturnal	Capinera 2008	aerial-hawking
Lepidoptera	Noctuidae	Noctuinae	Leucania		BOLD:AAJ2503	volant	Roff 1994	nocturnal	Capinera 2008	aerial-hawking
Lepidoptera	Noctuidae	Noctuinae	Lithophane	socia	BOLD:AAE6607	volant	Roff 1994	nocturnal	Capinera 2008	aerial-hawking
Lepidoptera	Noctuidae	Noctuinae	Spodoptera	exigua	BOLD:AAA6644	volant	Roff 1994	nocturnal	Capinera 2008	aerial-hawking
Lepidoptera	Noctuidae	Noctuinae	Spodoptera	cilium	BOLD:AAC8279	volant	Roff 1994	nocturnal	Capinera 2008	aerial-hawking
Lepidoptera	Noctuidae	Amphipyrinae			BOLD:AAH5319	volant	Roff 1994	nocturnal	Capinera 2008	aerial-hawking
Lepidoptera					BOLD:ADG3545	volant	Roff 1994	undecided		undecided
Lepidoptera					BOLD:AAB6211	volant	Roff 1994	undecided		undecided
Lepidoptera	Noctuidae	Noctuinae			BOLD:AAB6980	volant	Roff 1994	nocturnal	Capinera 2008	aerial-hawking
Lepidoptera	Noctuidae	Noctuinae	Xylena		BOLD:AAE4735	volant	Roff 1994	nocturnal	Capinera 2008	aerial-hawking
Lepidoptera	Nolidae	Chloephorinae	Nycteola		BOLD:AAE2641	volant	Roff 1994	nocturnal	Tsukiji 2020 (2021/05/18)	aerial-hawking
Lepidoptera	Nolidae	Nolinae	Nola	confusalis	BOLD:AAB5563	volant	Roff 1994	nocturnal	Tsukiji 2020 (2021/05/18)	aerial-hawking
Lepidoptera	Nolidae	Nolinae			BOLD:AAL7275	volant	Roff 1994	nocturnal	Kawahara et al. 2017	aerial-hawking
Lepidoptera	Nolidae	Chloephorinae	Pseudoips	prasinana	BOLD:AAB8807	volant	Roff 1994	nocturnal	Tsukiji 2020 (2021/05/18)	aerial-hawking
Lepidoptera	Notodontidae				BOLD:AAM3801	volant	Roff 1994	nocturnal	Capinera 2008	aerial-hawking
Lepidoptera	Notodontidae	Notodontinae			BOLD:AAL6465	volant	Roff 1994	nocturnal	Capinera 2008	aerial-hawking
Lepidoptera	Notodontidae	Heterocampinae			BOLD:AAM4825	volant	Roff 1994	nocturnal	Capinera 2008	aerial-hawking
Lepidoptera	Notodontidae	Notodontinae	Notodonta		BOLD:AAC1146	volant	Roff 1994	nocturnal	Capinera 2008	aerial-hawking
Lepidoptera	Notodontidae	Notodontinae	Shaka		BOLD:ACJ1076	volant	Roff 1994	nocturnal	Capinera 2008	aerial-hawking
Lepidoptera	Notodontidae	Heterocampinae	Stauropus	fagi	BOLD:AAD0646	volant	Roff 1994	nocturnal	Capinera 2008	aerial-hawking
Lepidoptera	Nymphalidae	Heliconiinae	Boloria		BOLD:AAB9155	volant	Roff 1994	diurnal	Kawahara et al. 2017	gleaning
Lepidoptera	Oecophoridae	Oecophorinae			BOLD:AAF9734	volant	Roff 1994	nocturnal	Capinera 2008	aerial-hawking
Lepidoptera	Papilionidae	Papilioninae	Papilio		BOLD:AAI5285	volant	Roff 1994	diurnal	Capinera 2008	gleaning
Lepidoptera	Pyralidae	Pyralinae	Endotricha	olivacealis	BOLD:ACI9403	volant	Roff 1994	nocturnal	Tsukiji 2020 (2021/05/18)	aerial-hawking
Lepidoptera	Saturniidae				BOLD:AAA5428	volant	Roff 1994	nocturnal	Capinera 2008	aerial-hawking
Lepidoptera	Saturniidae	Saturniinae	Saturnia		BOLD:AAB9581	volant	Roff 1994	nocturnal	Capinera 2008	aerial-hawking

Order	Family	Subfamily	Genus	Species	BIN	Flight capability	Reference	Diel activity	Reference	Mode of capture
Lepidoptera	Saturniidae	Saturniinae	Saturnia	jonasii	BOLD:AAD1452	volant	Roff 1994	nocturnal	Capinera 2008	aerial-hawking
Lepidoptera	Sphingidae	Smerinthinae	Callambulyx		BOLD:ABY4871	volant	Roff 1994	nocturnal	Capinera 2008	aerial-hawking
Lepidoptera	Sphingidae	Macroglossinae			BOLD:AAB0931	volant	Roff 1994	nocturnal	Capinera 2008	aerial-hawking
Lepidoptera	Sphingidae	Sphinginae	Sphinx	ligustri	BOLD:AAB6107	volant	Roff 1994	nocturnal	Capinera 2008	aerial-hawking
Lepidoptera	Sphingidae	Sphinginae	Sphinx		BOLD:ACF5246	volant	Roff 1994	nocturnal	Capinera 2008	aerial-hawking
Lepidoptera	Stathmopodidae	NA	Stathmopoda	pedella	BOLD:AAD4282	volant	Roff 1994	diurnal	Kawahara et al. 2017	gleaning
Lepidoptera	Tineidae	Scardiinae	Morophaga	bucephala	BOLD:AAG8510	volant	Roff 1994	nocturnal	Capinera 2008	aerial-hawking
Lepidoptera	Tortricidae	Tortricinae			BOLD:AAC3136	volant	Roff 1994	nocturnal	Kawahara et al. 2017	aerial-hawking
Lepidoptera	Tortricidae	Tortricinae	Archips	betulana	BOLD:ACM3437	volant	Roff 1994	nocturnal	Kawahara et al. 2017	aerial-hawking
Lepidoptera	Tortricidae	Tortricinae	Archips		BOLD:AAB5839	volant	Roff 1994	nocturnal	Kawahara et al. 2017	aerial-hawking
Lepidoptera	Tortricidae	Tortricinae	Choristoneura	diversana	BOLD:AAD8048	volant	Roff 1994	nocturnal	Kawahara et al. 2017	aerial-hawking
Lepidoptera	Tortricidae	Tortricinae			BOLD:ACT2120	volant	Roff 1994	nocturnal	Kawahara et al. 2017	aerial-hawking
Lepidoptera	Tortricidae	Tortricinae	Dichelia		ANICU1645-11	volant	Roff 1994	nocturnal	Kawahara et al. 2017	aerial-hawking
Lepidoptera	Tortricidae	Tortricinae	Eana	incanana	BOLD:AAD7476	volant	Roff 1994	nocturnal	Kawahara et al. 2017	aerial-hawking
Lepidoptera	Tortricidae	Olethreutinae	Epinotia		BOLD:AAE1784	volant	Roff 1994	nocturnal	Kawahara et al. 2017	aerial-hawking
Lepidoptera	Tortricidae	Olethreutinae	Epinotia	nisella	BOLD:AAA7530	volant	Roff 1994	nocturnal	Kawahara et al. 2017	aerial-hawking
Lepidoptera	Tortricidae	Olethreutinae	Eudemis	porphyrana	BOLD:AAC6854	volant	Roff 1994	nocturnal	Kawahara et al. 2017	aerial-hawking
Lepidoptera	Tortricidae	Olethreutinae	Gypsonoma	dealbana	BOLD:AAB0380	volant	Roff 1994	nocturnal	Kawahara et al. 2017	aerial-hawking
Lepidoptera	Tortricidae	Olethreutinae	Lobesia		BOLD:ABV8007	volant	Roff 1994	nocturnal	Kawahara et al. 2017	aerial-hawking
Lepidoptera	Tortricidae	Olethreutinae			BOLD:ABZ7645	volant	Roff 1994	nocturnal	Kawahara et al. 2017	aerial-hawking
Lepidoptera	Tortricidae	Olethreutinae	Olethreutes		BOLD:ACS0054	volant	Roff 1994	nocturnal	Kawahara et al. 2017	aerial-hawking
Lepidoptera	Tortricidae	Tortricinae	Pandemis	cinnamomeana	BOLD:AAD0575	volant	Roff 1994	nocturnal	Kawahara et al. 2017	aerial-hawking
Lepidoptera	Tortricidae	Tortricinae	Pandemis	corylana	BOLD:AAC5400	volant	Roff 1994	nocturnal	Kawahara et al. 2017	aerial-hawking
Lepidoptera	Tortricidae	Olethreutinae	Phiaris		BOLD:AAJ2026	volant	Roff 1994	nocturnal	Kawahara et al. 2017	aerial-hawking
Lepidoptera	Tortricidae	Tortricinae	Ptycholoma	lecheanum	BOLD:AAD3264	volant	Roff 1994	nocturnal	Kawahara et al. 2017	aerial-hawking
Lepidoptera	Tortricidae	Olethreutinae	Rhopobota	naevana	BOLD:AAA9812	volant	Roff 1994	nocturnal	Kawahara et al. 2017	aerial-hawking
Lepidoptera	Tortricidae	Olethreutinae			BOLD:AAA0213	volant	Roff 1994	nocturnal	Kawahara et al. 2017	aerial-hawking
Lepidoptera	Tortricidae	Olethreutinae	Spilonota	laricana	BOLD:AAA7739	volant	Roff 1994	nocturnal	Kawahara et al. 2017	aerial-hawking
Lepidoptera	Tortricidae	Tortricinae	Archips	crataeganus	BOLD:AAD6620	volant	Roff 1994	nocturnal	Kawahara et al. 2017	aerial-hawking
Lepidoptera					BOLD:AAH4639	volant	Roff 1994	undecided		undecided
Lepidoptera	Tortricidae	Olethreutinae	Epinotia		BOLD:AAN7425	volant	Roff 1994	nocturnal	Kawahara et al. 2017	aerial-hawking
Lepidoptera	Tortricidae	Olethreutinae			BOLD:AAV9504	volant	Roff 1994	nocturnal	Kawahara et al. 2017	aerial-hawking
Lepidoptera	Tortricidae				BOLD:ADE9961	volant	Roff 1994	nocturnal	Kawahara et al. 2017	aerial-hawking
Lepidoptera	Tortricidae	Tortricinae			BOLD:ACP8754	volant	Roff 1994	nocturnal	Kawahara et al. 2017	aerial-hawking
Lepidoptera	Tortricidae	Olethreutinae	Zeiraphera	rufimitrana	BOLD:AAM3356	volant	Roff 1994	nocturnal	Kawahara et al. 2017	aerial-hawking
Lepidoptera	Uraniidae	Uraniinae			BOLD:ACF4699	volant	Roff 1994	nocturnal	Capinera 2008	aerial-hawking
Lepidoptera	Yponomeutidae	Yponomeutinae			BOLD:AAE4109	volant	Roff 1994	diurnal	Kawahara et al. 2017	gleaning
Lepidoptera	Ypsolophidae	Ypsolophinae	Ypsolopha	vittella	BOLD:AAD9548	volant	Roff 1994	diurnal	Kawahara et al. 2017	gleaning
Lepidoptera					BOLD:AAD3218	volant	Roff 1994	undecided		undecided
Mecoptera	Panorpidae	Panorpinae	Panorpa	pryeri	BOLD:ACA3726	volant		diurnal	Tsukiji 2020 (2021/05/19)	gleaning
Neuroptera	Hemerobiidae	Drepanepteryginae	Drepanepteryx		BOLD:ACT2374	volant	Roff 1990	undecided	Kawahara et al. 2017	undecided
Neuroptera	Hemerobiidae	Hemerobiinae	Hemerobius		BOLD:AAG0897	volant	Roff 1990	nocturnal	Tsukiji 2020 (2021/05/18)	aerial-hawking
Neuroptera	Hemerobiidae	Hemerobiinae	Hemerobius		BOLD:ABZ0149	volant	Roff 1990	nocturnal	Tsukiji 2020 (2021/05/18)	aerial-hawking

Order	Family	Subfamily	Genus	Species	BIN	Flight capability	Reference	Diel activity	Reference	Mode of capture
Neuroptera	Hemerobiidae	Hemerobiinae	Hemerobius	fenestratus	BOLD:AAU3559	volant	Roff 1990	nocturnal	Tsukiji 2020 (2021/05/18)	aerial-hawking
Neuroptera	Hemerobiidae	Hemerobiinae	Hemerobius	simulans	BOLD:ACM1832	volant	Roff 1990	nocturnal	Tsukiji 2020 (2021/05/18)	aerial-hawking
Neuroptera	Hemerobiidae	Hemerobiinae			BOLD:ABU9030	volant	Roff 1990	undecided	Tsukiji 2020 (2021/05/18)	undecided
Neuroptera	Osmylidae				INRMA502-12	volant	Roff 1990	undecided	Tsukiji 2020 (2021/05/18)	undecided
Plecoptera	Nemouridae	Amphinemurinae			BOLD:AAL6222	volant	Roff 1994	nocturnal	Yoshimura 2014	aerial-hawking
Psocodea	Psocidae	Psocinae	Psococerastis		BOLD:ACC5474	volant	Roff 1994	diurnal	Tsukiji 2020 (2021/05/19)	gleaning
Trichoptera	Lepidostomatidae	Lepidostomatinae	Lepidostoma	elongatum	RUSST089-12	volant	Roff 1990	nocturnal	Capinera 2008	aerial-hawking
Trichoptera	Psychomyiidae	Psychomyiinae	Tinodes	higashiyamanus	BOLD:ACD6706	volant	Roff 1990	nocturnal	Capinera 2008	aerial-hawking
Trichoptera	Thremmatidae	Thremmatinae	Neophylax	ussuriensis	BOLD:AAG9568	volant	Roff 1990	nocturnal	Capinera 2008	aerial-hawking
Opiliones	Sclerosomatidae	Leiobuninae	Leiobunum	tohokuense	NA	non-volant				gleaning
Coleoptera	Elateridae	Elaterinae	Agriotes		NA	volant	Roff 1994	nocturnal	Capinera 2008	aerial-hawking
Coleoptera	Scarabaeidae	Rutelinae	Anomala		NA	volant	Roff 1994	undecided	Tsukiji 2020 (2021/05/18)	undecided
Coleoptera	Curculionidae	Curculioninae	Curculio		NA	volant	Roff 1994	diurnal	Tsukiji 2020 (2021/05/18)	gleaning
Coleoptera	Cerambycidae	Lamiinae	Eutetrapha		NA	volant	Roff 1994	diurnal	Tsukiji 2020 (2021/05/18)	gleaning
Coleoptera	Tenebrionidae	Alleculinae	Hymenalia		NA	volant	Roff 1994	undecided	Capinera 2008	undecided
Coleoptera	Scarabaeidae				NA	volant	Roff 1994	diurnal	Gill et al. 2012	gleaning
Coleoptera	Scarabaeidae				NA	volant	Roff 1994	diurnal	Gill et al. 2012	gleaning
Coleoptera	Melandryidae	Malandryinae	Phloiotrya	planiuscula	NA	volant	Roff 1994	nocturnal	Thomas et al. 2002	aerial-hawking
Coleoptera	Scarabaeidae				NA	volant	Roff 1994	diurnal	Gill et al. 2012	gleaning
Coleoptera	Cantharidae	Cantharinae	Podabrus		NA	volant	Roff 1994	diurnal	Tsukiji 2020 (2021/05/18)	gleaning
Coleoptera	Chrysomelidae				NA	volant	Roff 1994	diurnal	Jolivet&Pierre 1981	gleaning
Dermaptera	Forficulidae	Anechurinae	Anechura	harmandi	NA	undecided	Roff 1994	diurnal	Novella-Fernandez et al. 2020	gleaning
Dermaptera	Forficulidae	Forficulinae	Forficula	mikado	NA	undecided	Roff 1994	diurnal	Novella-Fernandez et al. 2020	gleaning
Diptera	Mycetophilidae	Gnoristinae	Boletina		NA	volant	Roff 1990, 1994	nocturnal	Mikkola 1972	aerial-hawking
Diptera	Tachinidae	Exoristinae	Botria	japonica	NA	volant	Roff 1990, 1994	diurnal	Novella-Fernandez et al. 2020	gleaning
Diptera	Sciaridae	NA	Chaetosciara		NA	volant	Roff 1990, 1994	nocturnal	Novella-Fernandez et al. 2020	aerial-hawking
Diptera	Calliphoridae	Chrysomyinae	Chrysomya		NA	volant	Roff 1990, 1994		Novella-Fernandez et al. 2020	gleaning
Diptera	Empidoidea				NA	volant	Roff 1990, 1994	undecided		undecided
Diptera	Culicidae	Culicinae	Culex		NA	volant	Roff 1990, 1994	nocturnal	Mikkola 1972	aerial-hawking
Diptera	Culicidae	Culicinae	Culex	tritaeniorhynchus	NA	volant	Roff 1990, 1994	nocturnal	Mikkola 1972	aerial-hawking
Diptera	Limoniidae	Limoniinae	Dicranomyia		NA	volant	Roff 1990, 1994	nocturnal	Novella-Fernandez et al. 2020	aerial-hawking
Diptera	Limoniidae	Limoniinae	Dicranomyia		NA	volant	Roff 1990, 1994	nocturnal	Novella-Fernandez et al. 2020	aerial-hawking
Diptera	Limoniidae	Limoniinae	Dicranomyia		NA	volant	Roff 1990, 1994	nocturnal	Novella-Fernandez et al. 2020	aerial-hawking
Diptera	Limoniidae	Limoniinae	Dicranomyia		NA	volant	Roff 1990, 1994	nocturnal	Novella-Fernandez et al. 2020	aerial-hawking
Diptera	Tachinidae	Exoristinae	Eumea	linearicornis	NA	volant	Roff 1990, 1994	diurnal	Novella-Fernandez et al. 2020	gleaning
Diptera	Mycetophilidae	Mycetophilinae	Exechia		NA	volant	Roff 1990, 1994	nocturnal	Mikkola 1972	aerial-hawking
Diptera	Mycetophilidae	Mycetophilinae	Exechia		NA	volant	Roff 1990, 1994	nocturnal	Mikkola 1972	aerial-hawking
Diptera	Tachinidae	Exoristinae	Gonia	chinensis	NA	volant	Roff 1990, 1994	diurnal	Tsukiji 2020 (2021/05/19)	gleaning
Diptera	Limoniidae				NA	volant	Roff 1990, 1994	nocturnal	Novella-Fernandez et al. 2020	aerial-hawking
Diptera	Chironomidae	Chironominae	Micropsectra		NA	volant	Roff 1990, 1994	nocturnal	Scudder & Cannings 2006 (2021/05/19)	aerial-hawking
Diptera	Chironomidae	Chironominae	Micropsectra		NA	volant	Roff 1990, 1994	nocturnal	Scudder & Cannings 2006 (2021/05/19)	aerial-hawking
Diptera	Mycetophilidae	Mycetophilinae	Mycetophila		NA	volant	Roff 1990, 1994	nocturnal	Mikkola 1972	aerial-hawking
Diptera	Mycetophilidae	Mycetophilinae	Mycetophila	fungorum	NA	volant	Roff 1990, 1994	nocturnal	Mikkola 1972	aerial-hawking

Order	Family	Subfamily	Genus	Species	BIN	Flight capability	Reference	Diel activity	Reference	Mode of capture
Diptera	Mycetophilidae	Mycomyinae	Mycomya		NA	volant	Roff 1990, 1994	nocturnal	Mikkola 1972	aerial-hawking
Diptera					NA	volant	Roff 1990, 1994	undecided		undecided
Diptera	Empidoidea				NA	volant	Roff 1990, 1994	undecided		undecided
Diptera	Limoniidae				NA	volant	Roff 1990, 1994	nocturnal	Novella-Fernandez et al. 2020	aerial-hawking
Diptera	Psychodidae				NA	volant	Roff 1990, 1994	nocturnal	Scudder & Cannings 2006 (2021/05/19)	aerial-hawking
Diptera	Rhagionidae				NA	volant	Roff 1990, 1994	undecided		undecided
Diptera	Tipulidae				NA	volant	Roff 1990, 1994	nocturnal	Mikkola 1972	aerial-hawking
Diptera	Culicidae	Culicinae	Ochlerotatus		NA	volant	Roff 1990, 1994	nocturnal	Mikkola 1972	aerial-hawking
Diptera	Pediciidae	Pediciinae	Pedicia		NA	volant	Roff 1990, 1994	nocturnal	Novella-Fernandez et al. 2020	aerial-hawking
Diptera	Rhinophoridae				NA	volant	Roff 1990, 1994	diurnal	Novella-Fernandez et al. 2020	gleaning
Diptera	Mycetophilidae	Mycetophilinae	Pseudobrachypeza		NA	volant	Roff 1990, 1994	nocturnal	Mikkola 1972	aerial-hawking
Diptera	Phoridae	Phorinae	Stichillus		NA	volant	Roff 1990, 1994	diurnal	Novella-Fernandez et al. 2020	gleaning
Diptera	Tachinidae	Exoristinae	Suensonomyia	nudinerva	NA	volant	Roff 1990, 1994	diurnal	Novella-Fernandez et al. 2020	gleaning
Diptera	Mycetophilidae				NA	volant	Roff 1990, 1994	nocturnal	Mikkola 1972	aerial-hawking
Diptera	Muscidae	Azeliinae	Thricops		NA	volant	Roff 1990, 1994	diurnal	Novella-Fernandez et al. 2020	gleaning
Diptera	Tipulidae	Tipulinae	Tipula		NA	volant	Roff 1990, 1994	nocturnal	Mikkola 1972	aerial-hawking
Diptera	Tipulidae	Tipulinae	Tipula		NA	volant	Roff 1990, 1994	nocturnal	Mikkola 1972	aerial-hawking
Diptera	Mycetophilidae	Mycetophilinae			NA	volant	Roff 1990, 1994	nocturnal	Mikkola 1972	aerial-hawking
Ephemeroptera	Heptageniidae	Rhithrogeniinae	Rhithrogena		NA	volant	Roff 1994	nocturnal	Capinera 2008	aerial-hawking
Ephemeroptera	Heptageniidae	Rhithrogeniinae	Rhithrogena		NA	volant	Roff 1994	nocturnal	Capinera 2008	aerial-hawking
Hemiptera	Miridae	Mirinae	Adelphocoris		NA	volant	Roff 1994	diurnal	Capinera 2008	gleaning
Hemiptera	Miridae	Deraeocorinae	Alloeotomus	simplus	NA	volant	Roff 1994	diurnal	Capinera 2008	gleaning
Hemiptera	Miridae	Mirinae	Arbolygus	rubripes	NA	volant	Roff 1994	diurnal	Capinera 2008	gleaning
Hemiptera	Acanthosomatidae	Acanthosomatinae	Elasmucha	signoreti	NA	volant	Roff 1994	diurnal	Tsukiji 2020 (2021/05/19)	gleaning
Hemiptera	Miridae	Mirinae	Phytocoris		NA	volant	Roff 1994	diurnal	Capinera 2008	gleaning
Hemiptera	Psyllidae	Psyllinae	Psylla		NA	volant	Roff 1994	diurnal	Tsukiji 2020 (2021/05/19)	gleaning
Hemiptera	Psyllidae	Psyllinae	Psylla		NA	volant	Roff 1994	diurnal	Tsukiji 2020 (2021/05/19)	gleaning
Hemiptera	Aphididae	Calaphidinae	Symydobius	kabae	NA	volant	Roff 1994	diurnal	Tsukiji 2020 (2021/05/19)	gleaning
Hymenoptera	Braconidae	Rogadinae	Aleiodes		NA	volant	Roff 1990	nocturnal	Mikkola 1972	aerial-hawking
Hymenoptera	Ichneumonidae	Campopleginae	Hyposoter		NA	volant	Roff 1990	nocturnal	Mikkola 1972	aerial-hawking
Hymenoptera	Braconidae	Microgastrinae	Microplitis		NA	volant	Roff 1990	nocturnal	Mikkola 1972	aerial-hawking
Hymenoptera					NA	volant	Roff 1990	undecided		undecided
Hymenoptera	Formicidae				NA	volant	Roff 1990	nocturnal	Novella-Fernandez et al. 2020	aerial-hawking
Hymenoptera	Tenthredinidae	Nematinae	Nematinus	luteus	NA	volant	Roff 1990	diurnal	Novella-Fernandez et al. 2020	gleaning
Lepidoptera	Geometridae	Ennominae	Arichanna	melanaria	NA	volant	Roff 1994	nocturnal	Capinera 2008	aerial-hawking
Lepidoptera	Noctuidae				NA	volant	Roff 1994	nocturnal	Capinera 2008	aerial-hawking
Lepidoptera	Tortricidae	Tortricinae			NA	volant	Roff 1994	nocturnal	Kawahara et al. 2017	aerial-hawking
Trichoptera	Limnephilidae	Dicosmoecinae	Dicosmoecus		NA	volant	Roff 1990	nocturnal	Capinera 2008	aerial-hawking
Lepidoptera	Geometridae	Ennominae	Ectropis	obliqua	NA	volant	Roff 1994	nocturnal	Capinera 2008	aerial-hawking
Lepidoptera	Tortricidae	Tortricinae			NA	volant	Roff 1994	nocturnal	Kawahara et al. 2017	aerial-hawking
Lepidoptera	Geometridae	Ennominae	Jankowskia		NA	volant	Roff 1994	nocturnal	Capinera 2008	aerial-hawking
Lepidoptera	Geometridae	Larentiinae	Lobogonodes	erectaria	NA	volant	Roff 1994	nocturnal	Capinera 2008	aerial-hawking
Lepidoptera	Erebidae	Lymantriinae	Lymantria		NA	volant	Roff 1994	undecided	Tsukiji 2020 (2021/05/18)	undecided

Order	Family	Subfamily	Genus	Species	BIN	Flight capability	Reference	Diel activity	Reference	Mode of capture
Lepidoptera	Geometridae	Ennominae	Myrioblephara	cilicornaria	NA	volant	Roff 1994	nocturnal	Capinera 2008	aerial-hawking
Lepidoptera					NA	volant	Roff 1994	undecided		undecided
Lepidoptera	Noctuidae	Noctuinae	Panolis		NA	volant	Roff 1994	nocturnal	Capinera 2008	aerial-hawking
Lepidoptera	Noctuidae	Hadeninae	Panolis		NA	volant	Roff 1994	nocturnal	Capinera 2008	aerial-hawking
Lepidoptera	Noctuidae				NA	volant	Roff 1994	nocturnal	Capinera 2008	aerial-hawking
Lepidoptera	Noctuidae				NA	volant	Roff 1994	nocturnal	Capinera 2008	aerial-hawking
Lepidoptera	Thyrididae	Siculodinae	Pyrinioides	aurea	NA	volant	Roff 1994	diurnal	Capinera 2008	gleaning
Lepidoptera	Saturniidae	Saturniinae	Saturnia		NA	volant	Roff 1994	nocturnal	Capinera 2008	aerial-hawking
Mecoptera	Panorpidae	Panorpinae	Panorpa	takenouchii	NA	volant		diurnal	Tsukiji 2020 (2021/05/19)	gleaning
Neuroptera	Chrysopidae	Chrysopinae	Apertochrysa		NA	volant	Roff 1990	undecided	Kawahara et al. 2017	undecided
Neuroptera	Chrysopidae	Chrysopinae	Chrysoperla		NA	volant	Roff 1990	diurnal	Tsukiji 2020 (2021/05/18)	gleaning
Neuroptera	Chrysopidae	Chrysopinae	Chrysoperla		NA	volant	Roff 1990	diurnal	Tsukiji 2020 (2021/05/18)	gleaning
Neuroptera	Osmylidae	Osmylinae	Osmylus		NA	volant	Roff 1990	diurnal	Tsukiji 2020 (2021/05/18)	gleaning
Neuroptera	Hemerobiidae	Hemerobiinae			NA	volant	Roff 1990	undecided	Tsukiji 2020 (2021/05/18)	undecided
Odonata	Libellulidae	Sympetrinae	Sympetrum	frequens	NA	volant	Roff 1994	diurnal	Capinera 2008	gleaning
Odonata	Libellulidae	Sympetrinae	Sympetrum	infuscatum	NA	volant	Roff 1994	diurnal	Capinera 2008	gleaning
Orthoptera	Rhaphidophoridae	Aemodogryllinae			NA	non-volant	Leubner et al. 2016			gleaning
Orthoptera	Trigonidiidae	Nemobiinae	Dianemobius	nigrofasciatus	NA	non-volant	Leubner et al. 2016			gleaning
Orthoptera	Rhaphidophoridae	Aemodogryllinae	Diestrammena		NA	non-volant	Leubner et al. 2016			gleaning
Orthoptera	Rhaphidophoridae				NA	non-volant	Leubner et al. 2016			gleaning
Orthoptera	Rhaphidophoridae				NA	non-volant	Leubner et al. 2016			gleaning
Plecoptera	Perlidae	Acroneuriinae	Calineuria		NA	volant	Roff 1994	nocturnal	Yoshimura 2014	aerial-hawking
Plecoptera	Nemouridae				NA	volant	Roff 1994	nocturnal	Yoshimura 2014	aerial-hawking
Psocodea	Amphipsocidae	Amphipsocinae	Amphipsocus	japonicus	NA	volant	Roff 1994	diurnal		gleaning
Psocodea					NA	volant	Roff 1994	diurnal		gleaning
Psocodea	Mesopsocidae	NA	Mesopsocus	unipunctatus	NA	volant	Roff 1994	diurnal		gleaning
Psocodea	Psocidae	Psocinae	Psococerastis		NA	volant	Roff 1994	diurnal	Tsukiji 2020 (2021/05/19)	gleaning
Psocodea	Psocidae	Psocinae	Psococerastis	nubila	NA	volant	Roff 1994	diurnal	Tsukiji 2020 (2021/05/19)	gleaning
Psocodea	Stenopsocidae	NA	Stenopsocus	nigricellus	NA	volant	Roff 1994	diurnal	Tsukiji 2020 (2021/05/19)	gleaning
Psocodea	Psocidae	Psocinae	Trichadenotecnum		NA	volant	Roff 1994	diurnal		gleaning
Psocodea	Psocidae	Psocinae	Trichadenotecnum	incognitum	NA	volant	Roff 1994	diurnal		gleaning
Psocodea	Psocidae	Psocinae	Trichadenotecnum		NA	volant	Roff 1994	diurnal		gleaning
Thysanoptera	Thripidae	Thripinae	Thrips		NA	volant	Roff 1990	nocturnal	Sites et al. 1992	aerial-hawking
