# Supplementary material for: Dietary analysis reveals differences in the prey use of two sympatric bat species
Source: Ecol Evol. 2021 Dec 16;11(24):18651–61. doi: 10.1002/ece3.8472 (PMC8717349; doi:10.1002/ece3.8472)
Supplement: Supplementary file 4 — Appendix S4 [file ECE3-11-18651-s001.pdf]

Supplementary information S4 for *Dietary analysis reveals differences in the prey use of two sympatric bat species*

Olga Heim[1], Anna I.E. Puisto[2], Ilari Sääksjärvi[3], Dai Fukui[4] and Eero J. Vesterinen[5]*

1 Faculty of Life and Medical Sciences, Doshisha University, 610-0321 Kyotanabe, Japan

2 Centre for Population Health Research, University of Turku, 20014 Turku, Finland

3 Biodiversity Unit, University of Turku, 20014 Turku, Finland

4 The University of Tokyo Hokkaido Forest, The University of Tokyo, 079-1563 Furano, Japan

5 Department of Biology, University of Turku, 20014 Turku, Finland

List of checked species from the orders Coleoptea and Lepidoptera

Primer	Order	Family	Subfamily	Genus	Species	BIN	Pest [yes/no]	Crop/Host	Reference	Accessed
COI	Coleoptera	Carabidae	Harpalinae	Pterostichus	oblongopunctatus	BOLD:ABY4764	n	-	www.cabi.org	22-04-21
COI	Coleoptera	Cryptophagidae	Atomariinae	Atomaria	turgida	BOLD:AAJ9463	n	-	www.cabi.org	22-04-21
16S	Coleoptera	Melandryidae	Malandryinae	Phloiotrya	planiuscula		n	-	www.cabi.org	22-04-21
COI	Coleoptera	Nitidulidae	Nitidulinae	Cychramus	variegatus	BOLD:ABW4745	n	-	www.cabi.org	22-04-21
COI	Lepidoptera	Crambidae	Scopariinae	Eudonia	persimilis	BOLD:AAF1478	n	-	www.cabi.org	22-04-21
COI	Lepidoptera	Crambidae	Spilomelinae	Pleuroptya/Patania	expictalis	BOLD:AAL4502	n	-	www.cabi.org	22-04-21
COI	Lepidoptera	Drepanidae	Thyatirinae	Tetheella	fluctuosa	BOLD:AAD3647	n	-	www.cabi.org	22-04-21
COI	Lepidoptera	Erebidae	Erebinae	Thyas	juno	BOLD:AAF1405	y	nut and fruit trees	https://www.cabi.org/cpc/search/?q=Thyas+juno%0d%0a	22-04-21
COI	Lepidoptera	Erebidae	Lymantriinae	Lymantria	dispar	BOLD:AAA2052	y	deciduous and coniferous trees	https://www.cabi.org/isc/datasheet/31807#toimpact; http://www.ffpri-hkd.affrc.go.jp/group/konchu/Zukan/HTML/Lepi_Dokuga.htm	14-01-21
COI	Lepidoptera	Erebidae	Lymantriinae	Lymantria	monacha	BOLD:AAA5537	y	deciduous and coniferous trees	https://www.cabi.org/isc/datasheet/31811#todistribution; http://www.ffpri-hkd.affrc.go.jp/group/konchu/Zukan/HTML/Lepi_Dokuga.htm	14-01-21
COI	Lepidoptera	Gelechiidae	Gelechiinae	Gelechia	cuneatella	BOLD:AAF5086	n	-	www.cabi.org	22-04-21
COI	Lepidoptera	Gelechiidae	Gelechiinae	Psoricoptera	gibbosella	BOLD:AAD0608	y	oak	https://www.cabi.org/cpc/search/?q=Psoricoptera+gibbosella%0d%0a	14-01-21
16S	Lepidoptera	Geometridae	Ennominae	Arichanna	melanaria		n	-	www.cabi.org	22-04-21
COI	Lepidoptera	Geometridae	Ennominae	Cleora	insolita	BOLD:ACJ3813	n	-	www.cabi.org	22-04-21
COI	Lepidoptera	Geometridae	Ennominae	Deileptenia	ribeata	BOLD:AAC3800	n	-	www.cabi.org	22-04-21
COI	Lepidoptera	Geometridae	Ennominae	Ectropis	crepuscularia	BOLD:AAA2076	y	deciduous and coniferous trees	https://www.cabi.org/cpc/search/?q=Ectropis+crepuscularia%0d%0a	22-04-21
16S	Lepidoptera	Geometridae	Ennominae	Ectropis	obliqua		y	tea	1	
COI	Lepidoptera	Geometridae	Ennominae	Hypomecis	punctinalis	BOLD:ACA2461	n	-	www.cabi.org	22-04-21
COI	Lepidoptera	Geometridae	Ennominae	Menophra	senilis	BOLD:AAF3823	n	-	www.cabi.org	22-04-21
16S	Lepidoptera	Geometridae	Ennominae	Myrioblephara	cilicornaria		n	-	www.cabi.org	22-04-21
COI	Lepidoptera	Geometridae	Ennominae	Ourapteryx	maculicaudaria	BOLD:AAW9443	n	-	www.cabi.org	22-04-21
COI	Lepidoptera	Geometridae	Ennominae	Phthonosema	tendinosaria	BOLD:AAF6489	n	-	www.cabi.org	22-04-21
COI	Lepidoptera	Geometridae	Geometrinae	Jodis	putata	BOLD:ABZ4040	n	-	www.cabi.org	22-04-21
COI	Lepidoptera	Geometridae	Larentiinae	Gandaritis	fixseni	BOLD:AAY1335	n	-	www.cabi.org	22-04-21
16S	Lepidoptera	Geometridae	Larentiinae	Lobogonodes	erectaria		n	-	www.cabi.org	22-04-21
COI	Lepidoptera	Gracillariidae	Gracillariinae	Caloptilia	cf. heringi	BOLD:ADK1669	n	-	www.cabi.org	22-04-21

Primer	Order	Family	Subfamily	Genus	Species	BIN	Pest [yes/no]	Crop/Host	Reference	Accessed
COI	Lepidoptera	Gracillariidae	Gracillariinae	Caloptilia	hidakensis	BOLD:AAK1674	n	-	www.cabi.org	22-04-21
COI	Lepidoptera	Hesperiidae	Coeliadinae	Burara/Bibasis	aquilina	BOLD:ACD6545	y	deciduous and coniferous trees	www.ffpri-hkd.affrc.go.jp/group/konchu/Zukan/HTML/Lepi_Chou.htm	14-01-21
COI	Lepidoptera	Hesperiidae	Hesperiinae	Thoressa	varia	BOLD:ADK0840	n	-	www.cabi.org	22-04-21
COI	Lepidoptera	Lasiocampidae	Lasiocampinae	Euthrix	potatoria	BOLD:AAC1584	n	-	www.cabi.org	22-04-21
COI	Lepidoptera	Noctuidae	Acronictinae	Belciades	niveola	BOLD:ACU0724	n	-	www.cabi.org	22-04-21
COI	Lepidoptera	Noctuidae	Amphipyrinae	Brachionycha	nubeculosa	BOLD:AAE0860	n	-	www.cabi.org	22-04-21
COI	Lepidoptera	Noctuidae	Noctuinae	Apamea	helva	BOLD:AAC5412	n	-	www.cabi.org	22-04-21
COI	Lepidoptera	Noctuidae	Noctuinae	Lithophane	socia	BOLD:AAE6607	n	-	www.cabi.org	22-04-21
COI	Lepidoptera	Noctuidae	Noctuinae	Spodoptera	cilium	BOLD:AAC8279	y	vegtables	2	
COI	Lepidoptera	Noctuidae	Noctuinae	Spodoptera	exigua	BOLD:AAA6644	y	vegtables	3	
COI	Lepidoptera	Nolidae	Chloephorinae	Pseudoips	prasinana	BOLD:AAB8807	y	oak	https://www.cabi.org/cpc/search/?q=Pseudoips+prasinana%0d%0a	22-04-21
COI	Lepidoptera	Nolidae	Nolinae	Nola	confusalis	BOLD:AAB5563	n	-	www.cabi.org	22-04-21
COI	Lepidoptera	Notodontidae	Heterocampinae	Stauropus	fagi	BOLD:AAD0646	n	-	www.cabi.org	22-04-21
COI	Lepidoptera	Pyralidae	Pyralinae	Endotricha	olivacealis	BOLD:ACI9403	n	-	www.cabi.org	22-04-21
COI	Lepidoptera	Saturniidae	Saturniinae	Saturnia	jonasii	BOLD:AAD1452	n	-	www.cabi.org	22-04-21
COI	Lepidoptera	Sphingidae	Smerinthinae	Marumba	gaschkewitschii	BOLD:ABU7231	y	plum	https://www.agric.wa.gov.au/organisms/105875	14-01-21
COI	Lepidoptera	Sphingidae	Sphinginae	Sphinx	ligustri	BOLD:AAB6107	y	deciduous trees, shrubs	https://www.cabi.org/cpc/search/?q=Sphinx+ligustri%0d%0a	
COI	Lepidoptera	Stathmopodidae	NA	Stathmopoda	pedella	BOLD:AAD4282	n	-	www.cabi.org	22-04-21
16S	Lepidoptera	Thyrididae	Siculodinae	Pyrinioides	aurea		n	-	www.cabi.org	22-04-21
COI	Lepidoptera	Tineidae	Scardiinae	Morophaga	bucephala	BOLD:AAG8510	n	-	www.cabi.org	22-04-21
COI	Lepidoptera	Tortricidae	Olethreutinae	Epinotia	nisella	BOLD:AAA7530	n	-	www.cabi.org	22-04-21
COI	Lepidoptera	Tortricidae	Olethreutinae	Eudemis	porphyrana	BOLD:AAC6854	n	-	www.cabi.org	22-04-21
COI	Lepidoptera	Tortricidae	Olethreutinae	Gypsonoma	dealbana	BOLD:AAB0380	y	poplar species	https://www.cabi.org/isc/datasheet/26271#tohostsOrSpeciesAffected	22-04-21
COI	Lepidoptera	Tortricidae	Olethreutinae	Rhopobota	naevana	BOLD:AAA9812	y	fruit trees	4	
COI	Lepidoptera	Tortricidae	Olethreutinae	Spilonota	laricana	BOLD:AAA7739	y		https://www.cabi.org/isc/datasheet/51015#toplantTrade	22-04-21
COI	Lepidoptera	Tortricidae	Olethreutinae	Zeiraphera	rufimitrana	BOLD:AAM3356	y	fir species	https://www.cabi.org/cpc/search/?q=Zeiraphera+rufimitrana%0d%0a	22-04-21
COI	Lepidoptera	Tortricidae	Tortricinae	Acleris	paradiseana	BOLD:ADI2826	n	-	www.cabi.org	22-04-21
COI	Lepidoptera	Tortricidae	Tortricinae	Archips	betulana	BOLD:ACM3437	n	-	www.cabi.org	22-04-21
COI	Lepidoptera	Tortricidae	Tortricinae	Archips	crataeganus	BOLD:AAD6620	y	fruit trees	https://www.cabi.org/cpc/search/?q=Archips+crataeganus%0d%0a	22-04-21
COI	Lepidoptera	Tortricidae	Tortricinae	Choristoneura	diversana	BOLD:AAD8048	y	trees	北海道森林害虫図鑑 (affrc.go.jp)	14-01-21
COI	Lepidoptera	Tortricidae	Tortricinae	Eana	incanana	BOLD:AAD7476	n	-	www.cabi.org	22-04-21
COI	Lepidoptera	Tortricidae	Tortricinae	Pandemis	cinnamomeana	BOLD:AAD0575	y	fruit trees	https://www.cabi.org/cpc/search/?q=Pandemis+cinnamomeana%0d%0a	22-04-21
COI	Lepidoptera	Tortricidae	Tortricinae	Pandemis	corylana	BOLD:AAC5400	y	nut and fruit trees	https://www.cabi.org/cpc/search/?q=Pandemis+corylana	22-04-21
COI	Lepidoptera	Tortricidae	Tortricinae	Ptycholoma	lecheanum	BOLD:AAD3264	y	fruit trees	https://www.cabi.org/cpc/search/?q=Ptycholoma+lecheanum%0d%0a	22-04-21
COI	Lepidoptera	Ypsolophidae	Ypsolophinae	Ypsolopha	vittella	BOLD:AAD9548	n	-	www.cabi.org	22-04-21

References

1 YANG, Y., ZHANG, L., GUO, F., LONG, Y., WANG, Y. & WAN, X. 2016. Reidentification of sex pheromones of tea geometrid Ectropis obliqua Prout (Lepidoptera: Geometridae).*Journal of economic entomology,* 109**,** 167-175.

2 WATABIKI, D., YOSHIMATSU, S.-I., YOSHITAKE, H., BABA, Y., UESATO, T., SHIMATANI, M., IBUSUKI, H. & YUDA, T. 2013. Discrimination methods for Japanese pest species of Spodoptera Guenée (Lepidoptera: Noctuidae) lured by traps using a synthetic sex pheromone for Spodoptera exempta. *Japanese Journal of Applied Entomology and Zoology,* 57**,** 19-26.

3 XIA-LIN, Z., CONG, X.-P., WANG, X.-P. & LEI, C.-L. 2011. A review of geographic distribution, overwintering and migration in Spodoptera exigua Hübner (Lepidoptera: Noctuidae).*Journal of the Entomological Research Society,* 13**,** 39-48.

4 FITZPATRICK, S. M. & TROUBRIDGE, J. T. 1993. Fecundity, number of diapause eggs, and egg size of successive generations of the blackheaded fireworm (Lepidoptera: Tortricidae) on cranberries.*Environmental entomology,* 22**,** 818-823.
