# Supplementary material for: Dietary analysis reveals differences in the prey use of two sympatric bat species
Source: Ecol Evol. 2021 Dec 16;11(24):18651–61. doi: 10.1002/ece3.8472 (PMC8717349; doi:10.1002/ece3.8472)
Supplement: Supplementary file 5 — Appendix S5 [file ECE3-11-18651-s002.docx]

**Appendix**

***A1*** *Detailed laboratory methods*

We have collected some additional but significant information in this appendix to better enlighten the different steps in the laboratory and data analysis.

Our initial PCR setup to amplify bat species with primers SFF_145f and SFF_351r (Table A1.1) was carried out in 10 μl reaction volume containing 2 μl of DNA extract, 2.6 μl ddH_2_O, 5 μl MyTaq Red Mix polymerase mix (product number BIO-25048, Bioline, England), 200 nM forward primer, and 200 nM reverse primer. The cycling conditions were 10 min in 95°C, then 38 cycles of 60 s in 95°C, 30 s in 60°C and 30 s in 72°C, and a final extension stage for 10 min in 72°C. The PCR success was confirmed in subsequent electrophoresis, and successful PCR products were purified using A’SAP clean kit (product nr 80350, ArcticZymes, Trømssa, Norway). Purified samples were sent to Macrogen Inc. Europe (The Netherlands) for sequencing. The sequences were trimmed using Geneious version 6 (Kearse et al., 2012).

To amplify the potential arthropod prey, we used the two primer pairs ZBJ-ArtF1c and ZBJ-ArtR2c, and Ins16S-1F and Ins16S-1Rshort (Table A1.1) targeting the so-called DNA barcode region in the COI gene and the 16S gene region, respectively. Note that the fourth last nucleotide in the reverse primer targeting the 16S gene region was changed from G to R as used in Kaunisto et al. (2017). Reactions with these two primers were carried out in the same way as with the bat-specific primers except for the cycling conditions which are summarised in Table A1.2.

**Table A1.1** All the primers used in this study are listed in the following table. Annealing temperature and target fragment length (without primers) are given for each primer pair.

| Name | Sequence | Tm °C | Target length (bp) | Reference |
| --- | --- | --- | --- | --- |
| SFF_145f | GTHACHGCYCAYGCHTTYGTAATAAT | 60 | 202 | Walker et al. (2016) |
| SFF_351r | CTCCWGCRTGDGCWAGRTTTCC |  |  | Walker et al. (2016) |
| ZBJ-ArtF1c | AGATATTGGAACWTTATATTTTATTTTTGG | 53-61 | 157 | Zeale et al. (2011) |
| ZBJ-ArtR2c | WACTAATCAATTWCCAAATCCTCC |  |  | Zeale et al. (2011) |
| Ins16S-1F | TRRGACGAGAAGACCCTATA | 46, 56 | 156 | Clarke et al. (2014) |
| 16S-1Rshort | ACGCTGTTATCCCTAARGTA |  |  | Clarke et al. (2014) |

**Table A1.2** PCR programs for primers targeting the COI and 16S gene regions.

| **Primer**  **Process** |  | **ZBJ-ArtF1c and ZBJ-ArtR2c** | | |  | **Ins16S-1F and 16S-1Rshort** | | |
| --- | --- | --- | --- | --- | --- | --- | --- | --- |
|  |  | Temp.  [°C] | Time  [mm:ss] | No. of  cycles |  | Temp.  [°C] | Time  [mm:ss] | No. of  cycles |
| Initial denaturation |  | 95 | 03:00 |  |  | 95 | 03:00 |  |
| Denaturation |  | 95 | 00:30 | 16 |  | 95 | 00:15 | 5 |
| Annealing |  | 61  decreased by  0.5 per cycle | 00:30 |  |  | 46 | 00:30 |  |
| Extension |  | 72 | 00:30 |  |  | 72 | 00:15 |  |
| Denaturation |  | 95 | 00:30 | 24 |  | 95 | 00:15 | 35 |
| Annealing |  | 53 | 00:30 |  |  | 56 | 00:30 |  |
| Extension |  | 72 | 00:30 |  |  | 72 | 00:15 |  |
| Final extension |  | 72 | 10:00 |  |  | 72 | 10:00 |  |

## References

CLARKE, L. J., SOUBRIER, J., WEYRICH, L. S. & COOPER, A. 2014. Environmental metabarcodes for insects: in silicoPCR reveals potential for taxonomic bias. *Molecular Ecology Resources,* 14**,** 1160-1170.

KAUNISTO, K. M., ROSLIN, T., SÄÄKSJÄRVI, I. E. & VESTERINEN, E. J. 2017. Pellets of proof: First glimpse of the dietary composition of adult odonates as revealed by metabarcoding of feces. *Ecology and Evolution***,** n/a-n/a.

*A2* *Prey species assignation*

***COI data set***

ZOTUs were assigned to biological species based on all the sequences on BOLD systems. In cases, where BINs contained several species or genera names, we selected the next higher unique taxonomic level to be assigned to a given ZOTU. When high percentage identity (≥98) was retained, but species level name and BIN were missing (n=8), we first

1. tried to assign species manually by BLASTing ZOTUs to BOLD
2. and then to GenBank
3. and finally using knowledge of the local fauna in Hokkaido, Japan, to choose the most potential species.

Overall, 98.2% of total reads could be identified to species or higher prey taxa.

***16S data set***

ZOTUs were assigned to biological species based on all the sequences on GenBank nt database. When high percentage identity (≥98) was retained, but species name was missing (n=5), we used ProcessID and BOLD API tools from Linux command line to retrieve all the possible taxonomy for these records. When no species name was found using scripts, we tried to assign species manually by BLASTing ZOTUs to BOLD or GenBank and then using knowledge of the local fauna in Hokkaido, Japan.

Overall, 21.4% of reads could be identified to species or higher prey taxa, and 65.1% to bats themselves.

***Negative extraction controls and PCR blanks***

We checked all the reads in the control samples, finding only 7 (negative extraction controls) and 21 (PCR blanks) reads in the COI data set. Any sample having fewer reads in the shared ZOTUs with controls, were excluded from the analysis.

*A3 Prey species verification*

Based on the results of both markers (COI and 16S), we found that *Murina ussuriensis* consumed 234 prey items, of which 61 have been identified to the species level (with 59 in Hokkaido and 2 in Japan including Hokkaido), 78 to the genus level (with 72 in Hokkaido and 6 in Japan), 37 to the subfamily level (with 35 in Hokkaido and 2 in Japan), 45 to the family level (with 43 in Hokkaido and 2 in Japan) and 13 to the order level. *Myotis ikonnikovi* consumed 340 prey items of which 68 have been identified to the species level (with 49 in Hokkaido and 19 in Japan), 122 to the genus level (with 114 in Hokkaido and 8 in Japan), 55 to the subfamily level (with 52 in Hokkaido and 3 in Japan), 76 to the family level (with 67 in Hokkaido and 9 in Japan) and 19 to the order level.

Please find all the literature sources for each prey item in the PDF file of the Supplementary Information S1. This reference list is a summary of all the cited literature therein:

ABE, J., GANAHA-KIKUMURA, T. & YUKAWA, J. 2011. Morphological features, distribution, prey mites, and life history traits of Feltiella acarisuga (Vallot) (Diptera: Cecidomyiidae) in Japan. *Applied Entomology and Zoology,* 46**,** 271-279.

ADLER, P. H. & CROSSKEY, R. W. 2008. World blackflies (Diptera: Simuliidae): a fully revised edition of the taxonomic and geographical inventory. *Department of Entomology, Clemson University, South Carolina, 105p*.

AKITA, K. & MASUMOTO, K. 2012. New or Little-Known Tenebrionid Species (Coleoptera) from Japan.

BECCALONI, G. W. 2014. Cockroach Species File Online.

BEPPU, K. 2004. Seasonal change in microdistribution and adult age structure of Thricops diaphanus (Wiedemann, 1817) in central Japan (Diptera: Muscidae). *衛生動物,* 55**,** 39-45.

BONG-KYU, B., SHAN-CHUN, Y. & CHENG-DE, L. 2003. Revision of Tribe Archipini (Tortricidae: Tortricinae) in Northeast China. *Journal of Forestry Research,* 14**,** 93-102.

BUCHNER, P. & STĂNESCU, M. 2019. A revision of Agonopterix lacteella (Lepidoptera: Depressariidae) and establishing A. pallidior as a junior synonym. *Travaux du Muséum National d’Histoire Naturelle “Grigore Antipa”,* 62**,** 87.

BURNS, M., HEDIN, M. & TSURUSAKI, N. 2018. Population genomics and geographical parthenogenesis in Japanese harvestmen (Opiliones, Sclerosomatidae, Leiobunum). *Ecology and Evolution,* 8**,** 36-52.

BUTCHER, B. A., QUICKE, D. L. J., SHREEVIHAR, S. & RANJITH, A. P. 2016. Major range extensions for two genera of the parasitoid subtribe Facitorina, with a new generic synonymy (Braconidae, Rogadinae, Yeliconini). *ZooKeys***,** 109-120.

CHEN, F., WU, C. & XUE, D. 2010. A review of the genus Elophila Hübner, 1822 in China (Lepidoptera: Crambidae: Acentropinae). *Aquatic Insects,* 32**,** 35-60.

CHEN, H.-W. & AOTSUKA, T. 2003. Survey of the genus Leucophenga (Diptera, Drosophilidae) from Iriomote-jima, Japan, with descriptions of three new species. *The Canadian Entomologist,* 135**,** 143-158.

CHO, G., INOUE, H. & LEE, S. 2019. On Cacopsylla moiwasana (Kuwayama, 1908)(Hemiptera: Psylloidea: Psyllidae) associated with Sorbus (Rosaceae) with new synonymies. *Zootaxa,* 4571**,** 147-150.

CHO, H.-W. & ŚWIĘTOJAŃSKA, J. 2017. Larval morphology of Plagiosterna adamsii (Baly) and P. aenea (Linnaeus) with resurrection of Gastrolinoides Chûjô & Kimoto and P. formosana (Bates)(Coleoptera: Chrysomelidae: Chrysomelinae). *Zootaxa,* 4236**,** 381-391.

DEWALT, R. E., MAEHR, M. D., HOPKINS, H., NEU-BECKER, U. & STUEBER, G. 2020. Plecoptera Species File Online. .

DOBOSZ, R., MAKARKIN, V. N. & SERGEYEV, M. E. 2019. Contributions to the knowledge of the entomofauna of the Sikhote-Alin Biosphere Reserve. I. Neuropteroid insects: alderflies (Megaloptera: Sialidae), snake-flies (Raphidioptera) and lacewings (Neuroptera). *Annals of the Upper Silesian Museum in Bytom Entomology,* 28**,** 1-30.

ERZINCLIOGLU, Z. 1990. The larvae of two closely-related blowfly species of the genus Chrysomya (Diptera, Calliphoridae). *Entomologica Fennica,* 1**,** 151-153.

FONSECA, D. M., SMITH, J. L., KIM, H.-C. & MOGI, M. 2009. Population genetics of the mosquito Culex pipiens pallens reveals sex-linked asymmetric introgression by Culex quinquefasciatus. *Infection, Genetics and Evolution,* 9**,** 1197-1203.

HAYASHI, N. 1978. A contribution to the knowledge of the larvae of Nitidulidae occurring in Japan (Coleoptera: Cucujoidea). *Insecta matsumurana. New series: journal of the Faculty of Agriculture Hokkaido University, series entomology.,* 14**,** 1-97.

HAYASHI, T. 1986. Studies on the sphaerocerid flies of synanthropy and hygienic importance in Japan (Diptera): II. Records of the forty species. *Medical entomology and zoology,* 37**,** 193-204.

HAYASHI, T. 2010. The genus Opalimosina Rohacek (Diptera, Sphaeroceridae) from Japan. *Medical Entomology and Zoology,* 61**,** 309-319.

HORI, K., IWASA, M. & OGAWA, R. 1990. Biology of two species of the Protocalliphora (Diptera: calliphoridae) in Tokachi, Hokkaido, Japan: feeding behaviour of larvae, larval and pupal durations, voltinism and host specificity. *Applied Entomology and Zoology,* 25**,** 475-482.

IMADA, Y. & KATO, M. 2016. Bryophyte-feeding of Litoleptis (Diptera: Rhagionidae) with descriptions of new species from Japan. *Zootaxa,* 4097**,** 41-58.

ISHIHAMA, N. & HARA, H. 2006. The relationship between diversity of insect assemblages and stand condition in deciduous broad-leaved forests in Hokkaido, northern Japan. *Bulletin of the Hokkaido Forestry Research Institute (Japan)*.

ISHIKAWA, T. & MORIYA, S. 2019. A review of the stink bug genus Plautia Stål from Japan (Hemiptera, Heteroptera, Pentatomidae). *Zootaxa,* 4564**,** 470-490.

ISHIWATA, S. 2001. A Checklist of Japanese Ephemeroptera. *In:* BAE, Y. J. (ed.) *The 21" Century and Aquatic Entomology in East Asia.* Korea: Proc. 1• Symp. AESEA. Korean Soc. Aquatic Entomol.

ISONO, M., TAKEDA, S. & SAKURAI, H. 1986. The species composition and diversity of tree-dwelling beetles in deciduous oak and evergreen forests in central Japan; Relationship between the diversity index and sample size. *Ecological research,* 1**,** 269-278.

ITO, T. 2000. Caddisfly fauna of northernmost part of Japan. *Biol Inland Waters,* 15**,** 20-31.

ITO, T. 2017. Caddisfly (Trichoptera) fauna of Shumarinai-gawa River, Horokanai-cho, Hokkaido, northern Japan. *Biology of Inland Waters,* 32**,** 37-47.

IWASA, M. 2007. Review of the flies (Diptera) associated with animal dung and human feces in Japan. *Medical Entomology and Zoology,* 58**,** 155-166.

IWASA, M. & HORI, K. 1990. The calliphorid larvae parasitic on birds in Japan (Diptera: Calliphoridae). *Medical and veterinary entomology,* 4**,** 141-146.

IWASA, M., HORI, K. & AOKI, N. 1995. Fly fauna of bird nests in Hokkaido, Japan (Diptera). *The Canadian Entomologist,* 127**,** 613-621.

IWASAKI, A., HORI, Y. & YASUOKA, S. 2005. Biology of onion thrips (Thysanoptera: Thripidae) on welsh onion [Allium fistulosum] in Hokkaido [Japan] and control program of the pest based on economic thresholds. *Bulletin of Hokkaido Prefectural Agricultural Experiment Stations (Japan)*.

JIA, F. & WANG, Y. 2010. A revision of the species of Enochrus (Coleoptera: Hydrophilidae) from China. *Oriental Insects,* 44**,** 361-385.

KAIZUKA, J. & IWASA, M. 2015. Carabid beetles (Coleoptera: Carabidae) in coniferous plantations in Hokkaido, Japan: effects of tree species and environmental factors. *Entomological Science,* 18**,** 245-253.

KANEKO, J. 1993. Parasitic wasps of the silver Y moth, Autographa gamma (L.)(Noctuidae: Plusiinae) and the Asiatic common looper, A. nigrisigna (Walker) in Hokkaido, Japan. *Japanese Journal of Applied Entomology and Zoology,* 37**,** 22-24.

KANG, S. & SUH, S. J. 2017. A New Record of Scatella calida (Diptera: Ephydridae) to Korea, with a Key and a Checklist for the Genus. *Animal Systematics, Evolution and Diversity,* 33**,** 262-266.

KASHIZAKI, A. & HISAMATSU, S. 2011. New distribution records of two sap beetles (Coleoptera, Nitidulidae) from Hokkaido, Japan. *Elytra,* 1**,** 163-165.

KATO, D. 2019. Taxonomic notes on the genus Teucholabis Osten Sacken, 1860 (Diptera, Limoniidae) of Japan. *Euroasian Entomological Journal* 18**,** 248-254.

KATO, D. & TACHI, T. 2016. Revision of the Rhinophoridae (Diptera: Calyptratae) of Japan. *Zootaxa,* 4158**,** 81-92.

KAZANTSEV, S. 2001. Eight new species of the genus Rhagonycha (Cantharidae, Coleoptera) from Hokkaido, Japan. *Jpn. J. syst. Ent.,* 7**,** 269-277.

KAZANTSEV, S. V. 2011. An annotated checklist of Cantharoidea (Coleoptera) of Russia and adjacent territories. *Russian Entomological Journal,* 20**,** 387-410.

KHAGHANINIA, S., SHAKERYANI, A. & HAYAT, R. 2014. First record of two genera of hoverflies (Diptera: Syrphidae) from East Azerbaijan Province, Iran. *Entomofauna,* 35**,** 21-28.

KIYOKU, M. 1958. 松茸害虫の種類と被害. *岡山大学農学部学術報告,* 11**,** 49-59.

KOBAYASHI, H. & MATSUMOTO, T. 2011. Atlas of Japanese Scarabaeoidea, Volume 3: Phytophagous Group II. *Roppon-Ashi Entomological Books, Tokyo*.

KORSHUNOV, Y. P. & GORBUNOV, P. Y. 1995. Diurnal Butterflies of the Asian Part of Russia. *Ural National University,* 202.

KOSUDA, S., SASAKAWA, K. & IKEDA, H. 2016. Directional mitochondrial introgression and character displacement due to reproductive interference in two closely related Pterostichus ground beetle species. *Journal of evolutionary biology,* 29**,** 1121-1130.

KRZEMINSKA, E. 2001. New additions to the subgenus Trichocera (Trichocera) Meigen (Diptera: Trichoceridae). *Acta zoologica cracoviensia,* 44**,** 391-399.

LAFONTAINE, J. D. & SCHMIDT, B. C. 2013. Additions and corrections to the check list of the Noctuoidea (Insecta, Lepidoptera) of North America north of Mexico. *ZooKeys***,** 227.

LEE, S., HONG, K.-J., CHO, Y. S., CHOI, Y. S., YOO, M.-S. & LEE, S. 2017. Review of the subgenus Aethina Erichson s. str. (Coleoptera: Nitidulidae: Nitidulinae) in Korea, reporting recent invasion of small hive beetle, Aethina tumida. *Journal of Asia-Pacific Entomology,* 20**,** 553-558.

LIN, M.-Y., BI, W.-X. & YANG, X.-K. 2017. A revision of the genus Eutetrapha Bates (Coleoptera: Cerambycidae: Lamiinae: Saperdini). *Zootaxa,* 4238**,** 151-202.

LÖBL, I. & LÖBL, D. 2017. *Archostemata-Myxophaga-Adephaga*, Brill.

LÖBL, I. & SMETANA, A. 2008. *Tenebrionoidea*, BRILL.

LUO, Q., YAO, Y.-L., YANG, L. & CHEN, X.-S. 2019. A key to the bamboo-feeding genus Bambusana Anufriev (Hemiptera, Cicadellidae, Deltocephalinae, Athysanini), with description of one new species from China. *ZooKeys,* 861**,** 53-61.

LYUBARSKY, G. Y. 2014. Cryptophagidae (Coleoptera: Clavicornia) from China and adjacent regions. *Russian Entomological Journal,* 23**,** 19-40.

MAEDA, T. 2011. New species of Chersodromia from the Russian Far East (Diptera: Empidoidea: Hybotidae: Tachydromiinae). *Zootaxa,* 2979**,** 1-24.

MATSUMOTO, R. 2006. A list of the holotype-specimens of Diptera described by Dr. M. Sasakawa and co-workers, and deposited in the Osaka Museum of Natural History. *BULLETIN-OSAKA MUSEUM OF NATURAL HISTORY,* 60**,** 13.

MEN, Q., PODENAS, S. & STARKEVICH, P. 2019. Redescription of male and female genitalia of Tipula (Emodotipula) holoteles (Diptera), with notes on the structure of the semen pump in the subgenus Emodotipula.

MENZEL, F. 1999. Revision der paläarktischen Trauermücke (Diptera, Sciaridae) unter besonderer Berücksichtigung der deutschen Fauna.

MENZEL, F. & MOHRIG, W. 2000. *Revision der paläarktischen Trauermücken (Diptera: Sciaridae)*, Ampyx-Verlag.

MICHAILOVSKAYA, M. 1998. Phorid flies (Diptera, Phoridae) of the Kuril Islands. *Far Eastern Entomologist***,** 1-8.

MIHÁLYI, F. 1976. Ergebnisse der Albanien-Expedition 1961 des Deutschen Entomologischen Institutes. 91. Beitrag.(Diptera: Muscidae). *Beiträge zur Entomologie= Contributions to Entomology,* 26**,** 197-210.

MINOSHIMA, Y. N. 2019. Taxonomic status of Enoshrus vilis (Sharp) and E. uniformis (Sharp)(Coleoptera, Hydrophilidae). *Insecta matsumurana. New series: journal of the Faculty of Agriculture Hokkaido University, series entomology.,* 75**,** 1-18.

MIZUKOSHI, T. 2000. Damage by Three Pest Species, <I>Amara (Amara) chalcites, Phyllotreta striolata</I>, and <I>Delia platura</I>, to the Roots of the Japanese Radish, <I>Raphanus sativus</I>, in Hokkaido. *ANNUAL REPORT OF THE SOCIETY OF PLANT PROTECTION OF NORTH JAPAN,* 2000**,** 231-233.

MORIMOTO, K., NAKAMURA, T. & KANNÔ, K. 2015. The Insects of Japan, 4. Curculionidae: Entiminae (Part 2)(Coleoptera). Touka Shobo, Fukuoka.

NA, S.-M., LEE, D.-J. & BAE, Y.-S. 2018. Taxonomic review of Yponomeuta evonymella group in Korea, with a newly recorded species (Lepidoptera, Yponomeutidae, Yponomeutinae). *Journal of Asia-Pacific Biodiversity,* 11**,** 538-543.

NADOLNY, A. A., OMELKO, M. M., MARUSIK, Y. M. & BLAGOEV, G. 2016. A new species of spider belonging to the Pardosa lugubris-group (Araneae: Lycosidae) from Far East Asia. *Zootaxa,* 4072**,** 263-281.

NAGATOMI, A. 1968. MIYAGI, I. Notes on Korean species of the Canaceidae, with descriptions. *Insecta matsumurana,* 30**,** 129-142.

NAKAYAMA, H. & SHIMA, H. 2004. Revision of the genus Stichillus Enderlein of Japan (Diptera: Phoridae). *Entomological Science,* 7**,** 85-95.

NARTSHUK, E. 2017. First record of the grass flies (Diptera: Chloropidae) from Khabarovskii krai and Jewish Autonomous Region. *Far Eastern Entomologist***,** 21-28.

NISHIKAWA, M. 2009. Record of earwigs on Rishiri and Rebun Islands, northern Hokkaido, with references on dermaptera of Hokkaido, Japan. *Rishiri Studies***,** 61-65.

NIWAKA, G. & JIMBO, U. 2018. An Identification Guide of Japanese Moths Compiled by Everyone.: Niwaka, Gamania

Jimbo, Utsuji.

NOTSU, Y. 1994. Two new species of the genus Curculio from Japan (Coleoptera: Curculionidae). *Trans. Shikoku ent. Soc.,* 20**,** 265-267.

NOZAKI, T. 2020. A catalogue of Japanese Trichoptera Family Uenoidae Iwata.

O’HARA, J., HENDERSON, S. & WOOD, D. 2019. Preliminary checklist of the Tachinidae of the world. Version.

OBA, Y., ÔHIRA, H., MURASE, Y., MORIYAMA, A. & KUMAZAWA, Y. 2015. DNA Barcoding of Japanese Click Beetles (Coleoptera, Elateridae). *PLOS ONE,* 10**,** e0116612.

OH, K.-S. & CHO, Y.-B. 2015. Eight Stenus Latreille species (Coleoptera, Staphylinidae, Steninae) in Korea. *Korean J. Appl. Entomol,* 54**,** 317-326.

OKADA, I. 1939. Eine kleine Fungivoriden-Liste (Diptera) vom Berge Daisen in der Provinz Hoki (Honshu). *Insecta matsumurana,* 13**,** 97-100.

OKUDA, T. & HODEK, I. 1994. Diapause and Photoperiodic Response in Coccinella septempunctata brucki MULSANT (Coleoptera: Coccinellidae) in Hokkaido, Japan. *Applied Entomology and Zoology,* 29**,** 549-554.

ONO, H., IKEDA, H. & KONO, R. 2009. Salticidae. *In:* ONO, H. (ed.) *The Spiders of Japan with keys to the families and genera and illustrations of the species.*: Tokai Univ. Press.

OTERO, J. C., GHAHARI, H. & ANGELINI, F. 2017. Contribution to the knowledge of cryptophagids (Coleoptera: Cryptophagidae) from Iran. *Redia,* 100**,** 45-51.

PARK, K.-T. & KIM, S. 2017. Faunistic data of micromoths (Lepidoptera) in North Korea. *Journal of Asia-Pacific Biodiversity,* 10**,** 73-80.

PARK, K.-T., PARK, Y.-M. & KIM, J.-D. 2020. A new species and two unrecorded species of Lecithoceridae (Lepidoptera, Gelechioidea) from Korea, with a tentative checklist of the family. *Journal of Asia-Pacific Biodiversity*.

PENNY, N. D. & BYERS, G. W. 1979. A check-list of the Mecoptera of the world. *Acta Amazonica,* 9**,** 365-388.

POLEVOI, A. & BARKALOV, A. 2017. Fungus gnats (Diptera: Bolitophilidae, Diadocidiidae, Keroplatidae, Mycetophilidae) of the lower course of Anadyr River, Chukotskii Autonomnyi Okrug, Russia. *Евразиатский энтомологический журнал,* 16**,** 119-128.

POORANI, J., PADMANABAN, B. & THANIGAIRAJ, R. 2019. NATURAL ENEMIES OF BANANA LACEWING BUG, STEPHANITIS TYPICA (DISTANT) IN INDIA, INCLUDING FIRST REPORT OF ANAGRUS SP.(HYMENOPTERA: MYMARIDAE) AS ITS EGG PARASITOID. *Munis Entomology & Zoology,* 14**,** 83-87.

PROKHOROV, A., POPOV, G. & ZAIKA, M. 2018. b. Rare species of hoverflies (Diptera: Syrphidae) from Ukraine. II. Callicerini, Cerioidini and Milesiini. *Ukrainska Entomofaunistyka,* 9**,** 15-22.

PUTHZ, V. 2010. A new species of the genus Euaesthetus Gravenhorst (Coleoptera: Staphylinidae) from Japan. *Entomological Review of Japan,* 65**,** 11-14.

QIAO, G. X. & ZHANG, G. X. 2002. A review of the genus Symydobius Mordvilko (Homoptera: Aphididae: Myzocallidinae) from China, with descriptions of one new subgenus and three new species. *Journal of the Kansas Entomological Society***,** 241-251.

RATNASINGHAM, S. & HEBERT, P. D. 2007. BOLD: The Barcode of Life Data System (http://www. barcodinglife. org). *Molecular ecology notes,* 7**,** 355-364.

ROSKOV, Y., OWER, G., ORRELL, T., NICOLSON, D., BAILLY, N., KIRK, P. M., BOURGOIN, T., DEWALT, R. E., DECOCK, W., VAN NIEUKERKEN, E., ZARUCCHI, J. & PENEV, L. 2019. Species 2000 & ITIS Catalogue of Life, 2019 Annual Checklist. Leiden, the Netherlands: Species 2000: Naturalis.

SAMIN, N., VAN ACHTERBERG, C. & ERDOĞAN, Ö. Ç. 2016. A faunistic study on some subfamilies of Braconidae (Hymenoptera: Ichneumonoidea) from Iran. *Arquivos entomolóxicos***,** 153-161.

SANJOBA, C., ÖZBEL, Y., ASADA, M., OSADA, Y., GANTUYA, S. & MATSUMOTO, Y. 2011. Recent collections of Sergentomyia squamirostris (Diptera: Psychodidae) in Japan, with descriptions and illustrations. *Medical Entomology and Zoology,* 62**,** 71-77.

SASA, M. & SUZUKI, H. 2000. Studies on the chironomid species collected at five localities in Hokkaido in September, 1998 (Diptera, Chironomidae). *熱帯医学 Tropical medicine,* 42**,** 175-199.

SASA, M. & SUZUKI, H. 2001. Studies on the chironomid species collected in Hokkaido in September, 2000. *熱帯医学 Tropical medicine,* 43**,** 1-38.

SASAKAWA, M. 2015. Notes on the Japanese Agromyzidae (Diptera), 6. *Scientific Reports of Kyoto Prefectural University, Life and Environmental Sciences***,** 9-48.

SAVCHENKO, E., OOSTERBROEK, P. & STARY, J. 1992. Family Limoniidae. *Catalogue of palaearctic diptera,* 1**,** 183-369.

SAWABE, K., HOSHINO, K., ISAWA, H., SASAKI, T., HAYASHI, T., TSUDA, Y., KURAHASHI, H., TANABAYASHI, K., HOTTA, A. & SAITO, T. 2006. Detection and isolation of highly pathogenic H5N1 avian influenza A viruses from blow flies collected in the vicinity of an infected poultry farm in Kyoto, Japan, 2004. *The American journal of tropical medicine and hygiene,* 75**,** 327-332.

SAYAMA, K., ITO, M., TABUCHI, K., UEDA, A., OZAKI, K. & HIRONAGA, T. 2012. Seasonal trends of forest moth assemblages in Central Hokkaido, Northern Japan. *The Journal of the Lepidopterists' Society,* 66**,** 11-26.

SEONG, J. & LEE, S. 2007. Taxonomic Notes on Alloeotomus Species (Heteroptera: Miridae: Deraeocorinae) in Korea. *Journal of Asia-Pacific Entomology,* 10**,** 317-322.

ŠEVČÍK, J. & PAPP, L. 2004. Bolitophilidae (Diptera) from Taiwan: a family new to the Oriental region. *Acta Zoologica Academiae Scientiarum Hungaricae,* 50**,** 55-62.

SIMBAQUEBA, R., SERNA, F. & MILLER, G. 2016. First record of Takecallis taiwana (Takahashi) and T. arundinariae (Essig)(Hemiptera: Aphididae) in Colombia. *Agronomía Colombiana,* 34**,** 295-299.

SIMONSEN, T. J. 2005. Boloria phylogeny (Lepidoptera: Nymphalidae): tentatively reconstructed on the basis of male and female genitalic morphology. *Systematic Entomology,* 30**,** 653-665.

SOROKINA, V. S., VIKHREV, N. E. & TRIDRIKH, N. N. A preliminary list of the Muscidae (Diptera) of the Magadan region, Russia. Annales de la Société entomologique de France (NS), 2018. Taylor & Francis, 318-334.

SUEYOSHI, M. & MATHIS, W. N. 2004. A new species of Cyamops Melander 1913 (Diptera: Periscelididae) from Japan and a review of Japanese Periscelididae. *Proceedings of the Entomological Society of Washington,* 106**,** 74-84.

SUWA, M. 1999. JAPANESE RECORDS OF ANTHOMYIID FILES (DIPTERA: ANTHOMYIIDAE). *Insecta matsumurana. New series: journal of the Faculty of Agriculture Hokkaido University, series entomology.,* 55**,** 203-244.

SUWA, M. 2018. Supplementary notes on the family Anthomyiidae of Japan (Diptera), VIII. *Insecta matsumurana. New series: journal of the Faculty of Agriculture Hokkaido University, series entomology.,* 74**,** 1-36.

SUZUKI, S. 1998. The distribution of chironomidis (Diptera: Chironomidae) on the foot of Mt. Yotei, Hokkaido, Japan. *Japanese Journal of Veterinary Research,* 46**,** 140-140.

TAKASHIMA, I., HASHIMOTO, N., WATANABE, T. & ROSEN, L. 1989. Mosquito Collection in Endemic Areas of Japanese Encephalitis in Hokkaido, Japan. *The Japanese Journal of Veterinary Science,* 51**,** 947-953.

TANAKA, K. 1999. Studies on the pupal mosquitoes (Diptera, Culicidae) of Japan (1) Aedes (Ochlerotatus). *The Japanese Journal of Systematic Entomology,* 5**,** 105-124.

TODA, M. J. 1987. Vertical Microdistribution of Drosophilidae (Diptera) within Various Forests in Hokkaido: Ⅲ. The Tomakomai Experiment Forest, Hokkaido University. *北海道大學農學部 演習林研究報告,* 44**,** 611-632.

TOGASHI, I. 2007. Sawflies of the Nematinus luteus group (Insecta: Hymenoptera: Tenthredinidae) from Japan. *Bulletin of the National Museum of Nature and Science, Series A (Zoology). Tokyo,* 33**,** 85-92.

TOGASHI, K., KASUGA, H., YAMASHITA, H. & IGUCHI, K. 2010. Larval diapause of Monochamus urussovi and photoperiodic effects on larval development in tree bolts. *Journal of applied entomology,* 134**,** 672-674.

TSUKAGUCHI, S. & TAGO, T. 2018. Kuwayamachrysa, a new genus of lacewings (Insecta, Neuroptera, Chrysopidae, Chrysopinae, Chrysopini) with markedly divergent adult and larval features. *Bulletin of the National Museum of Nature and Science, Series A,* 44**,** 69-85.

TSUKIJI, T. 2020. Mushi Navi. Tsukiji Takuro.

UCHIDA, S. 1983. A New Species of Calineuria (Plecoptera, Perlidae) from Japan, with Notes on the Japanse Species of the Genus. *昆蟲,* 51**,** 622-627.

VERVES, Y. G. & KHROKALO, L. 2006. Review of Macronychiinae (Diptera, Sarcophagidae) of the world.

VIKHREV, N. 2009. A new species of Coenosia Meigen (Diptera, Muscidae) from Kunashir Island. *ZooKeys,* 8**,** 35.

VIKHREV, N. 2015. Review of the Hydrotaea parva and Hydrotaea glabricula groups (Diptera: Muscidae). *Russian Entomological Journal,* 24**,** 93-102.

WAGNER, R., BARTÁK, M., BORKENT, A., COURTNEY, G., GODDEERIS, B., HAENNI, J.-P., KNUTSON, L., PONT, A., ROTHERAY, G. E., ROZKOŠNÝ, R., SINCLAIR, B., WOODLEY, N., ZATWARNICKI, T. & ZWICK, P. 2008. Global diversity of dipteran families (Insecta Diptera) in freshwater (excluding Simulidae, Culicidae, Chironomidae, Tipulidae and Tabanidae). *Hydrobiologia,* 595**,** 489-519.

WATANABE, K. 2015. Notes on Three Japanese Species of the Genus Cidaphus Förster, 1869 (Hymenoptera: Ichneumonidae: Mesochorinae). *Japanese Journal of Systematic Entomology,* 21**,** 61-64.

WATANABE, K. 2019. Revision of the Genus Pyracmon (Insecta: Hymenoptera: Ichneumonidae) from Japan, with Description of a New Species. *Species Diversity,* 24**,** 281-285.

YAGINUMA, T. 1972. Spiders of the Hidaka mountain range, Hokkaido, Japan. *Nat Sci Mus Tokyo*.

YAMAMOTO, M. 1996. Redescription of two species of the genus Glyptotendipes (Diptera, Chironomidae) from Japan. *Japanese Journal of Entomology,* 64**,** 465-472.

YAMAMOTO, M. 2004. A catalog of Japanese Orthocladiinae (Diptera : Chironomidae). *Makunagi,* 21**,** 1-121.

YAMAMOTO, M., YAMAMOTO, N. & KIMURA, M. 2015. Taxonomic notes on Chironomidae (Diptera) from Okinawa Island, Japan, with the description of three new species. *European Journal of Environmental Sciences,* 5**,** 101-115.

YANG, L. & WEAVER III, J. 2002. The Chinese Lepidostomatidae (Trichoptera). *Tijdschrift voor Entomologie,* 145**,** 267-352.

YASUNAGA, T. & SCHWARTZ, M. D. 2015. Review of the mirine plant bug genus Phytocoris Fallén in Japan (Hemiptera: Heteroptera: Miridae: Mirinae), with descriptions of eight new species. *Tijdschrift voor Entomologie,* 158**,** 21-47.

YONES, D. A., BAKIR, H. Y. & HAMEED, D. A. 2013. Human urogenital myiasis caused by Psychoda species larvae: report of five cases and morphological studies. *History*.

YOSHIDA, K. 1991. Biogeographic Perspective of Macrolepidopterous Fauna from the Tomakomai Experiment Forest, Southern Hokkaido. *Lepidoptera Science,* 42**,** 95-105.

YOSHIZAWA, K. 1998. Taxonomic Study of the Genus Mesopsocus Kolbe. *Entomological Science,* 1**,** 265-269.

YOSHIZAWA, K. 2001. Systematic study of Amphipsocidae in Japan (Psocodea:'Psocoptera': Caeciliusetae), with comments on higher classification within the family. *Insecta matsumurana. New series: journal of the Faculty of Agriculture Hokkaido University, series entomology.,* 58**,** 1-25.

YOSHIZAWA, K. 2002. Type specimens of Psocoptera described by H. Okamoto in Hokkaido University Insect Collection. *Insecta matsumurana. New series: journal of the Faculty of Agriculture Hokkaido University, series entomology.,* 59**,** 29-38.

YOSHIZAWA, K. 2004. Molecular phylogeny of major lineages of Trichadenotecnum and a review of diagnostic morphological characters (Psocoptera: Psocidae). *Systematic Entomology,* 29**,** 383-394.

YOSII, R. 1977. Critical check list of the Japanese species of Collembola.

YOUNG, C. W., LI, Y., CHU, W.-C. & FANG, H.-S. 2013. Review of Acutipula Crane Flies of Taiwan, with Descriptions of New Species and Immature Instars (Diptera: Tipulidae: Tipulinae: Tipula). *Annals of Carnegie Museum,* 82**,** 115-148.

ZHANG, C. & KURAHASHI, H. 2000. Two new species of the genus Helina from Japan: Diptera: Muscidae. *Medical Entomology and Zoology,* 51**,** 21-26.

ZHANG, L., LI, H., LI, S., ZHANG, A., KOU, F., XUN, H., WANG, P., WANG, Y., SONG, F., CUI, J., CUI, J., GOUGE, D. H. & CAI, W. 2015. Phylogeographic structure of cotton pest Adelphocoris suturalis (Hemiptera: Miridae): strong subdivision in China inferred from mtDNA and rDNA ITS markers. *Scientific Reports,* 5**,** 14009.

ВИХРЕВ, Н. Е. & ЕРОФЕЕВА, Е. А. 2018. Обзор видов группы Phaonia pallida (Diptera: Muscidae). *Russian Entomological Journal,* 27**,** 315-322.

ЛЕЛЕЙ, А., АЛЕКСЕЕВ, В., БЕЛОКОБЫЛЬСКИЙ, С., ГУМОВСКИЙ, А., ДАВИДЬЯН, Е., ЗЕРОВА, М., КАСПАРЯН, Д., КОЛЯДА, В., КОНОНОВА, С. & КОТЕНКО, А. 2012. Аннотированный каталог насекомых Дальнего Востока России. Том I. Перепончатокрылые.–. *Владивосток: Дальнаука*.

ЛЕЛЕЙ, А., АЛЕКСЕЕВ, В., БЕЛОКОБЫЛЬСКИЙ, С., ГУМОВСКИЙ, А., ДАВИДЬЯН, Е., ЗЕРОВА, М., КАСПАРЯН, Д., КОЛЯДА, В., КОНОНОВА, С. & КОТЕНКО, А. 2016. Аннотированный каталог насекомых Дальнего Востока России. Том II. Lepidoptera – Чешуекрылые. – Владивосток: Дальнаука, ISBN 978–5–8044–1576–2

中田和義, 中岡利泰 & 五嶋聖治 2006. 移入種ブラウントラウトが淡水産甲殻類に及ぼす影響: 絶滅危惧種ニホンザリガニへの捕食. *日本水産学会誌,* 72**,** 447-449.

寺山守, 富岡康浩, 神戸嘉一, 木村悟朗 & 谷川力 2020. 本土各地で確認された家屋害虫フシナガニセハリアリ. *衛生動物,* 71**,** 51-54.

小松謙之 2019. 本州で定着が確認されたサツマツチゴキブリ. *ペストロジー,* 34**,** 37-38.

小松謙之, 川上泰, 坂西梓里 & 内田明彦 2015. ビル内におけるトビイロゴキブリの分布とその季節消長に関する研究. *ペストロジー,* 30**,** 1-5.

杉浦真治, 深澤遊 & 山崎一夫 2002. 変形菌子実体から羽化したキノコバエ Platurocypta punctum (Stannius). *昆蟲. ニューシリーズ,* 5**,** 143-144.

笹川滿廣 2003. 日本産双翅目ノート 2. *昆蟲. ニューシリーズ,* 6**,** 119-133.

*A4 Classification of prey items*

This is a list of literature and online-resources used to classify prey items into categories of likely flight capability (*volant*, *non-volant*) and diel activity (*nocturnal*, *diurnal*):

CAPINERA, J. L. 2008. *Encyclopedia of Entomology*, Springer Netherlands.

GILL, H. K., GOYAL, G. & MCSORLEY, R. 2012. Diel activity of fauna in different habitats sampled at the autumnal equinox. *Florida Entomologist***,** 319-325.

HONĚK, A. & HODEK, I. 1996. Distribution in Habitats. *Ecology of Coccinellidae.* Dordrecht: Springer Netherlands.

JOLIVET, P. 1981. Biology of" Chrysomelidae""(Coleoptera)". *Butlletí de la Institució Catalana d'Història Natural***,** 105-138.

KAWAHARA, A., PLOTKIN, D., HAMILTON, C., GOUGH, H., ST LAURENT, R., OWENS, H., HOMZIAK, N. & BARBER, J. 2017. Diel behavior in moths and butterflies: a synthesis of data illuminates the evolution of temporal activity. *Organisms Diversity & Evolution,* 18.

LEUBNER, F., HÖRNSCHEMEYER, T. & BRADLER, S. 2016. The thorax of the cave cricket Troglophilus neglectus: anatomical adaptations in an ancient wingless insect lineage (Orthoptera: Rhaphidophoridae). *BMC Evolutionary Biology,* 16.

MIKKOLA, K. Behavioural and electrophysiological responses of night-flying insects, especially Lepidoptera, to near-ultraviolet and visible light. Annales zoologici fennici, 1972. JSTOR, 225-254.

NOVELLA‐FERNANDEZ, R., IBAÑEZ, C., JUSTE, J., CLARE, E. L., DONCASTER, C. P. & RAZGOUR, O. 2020. Trophic resource partitioning drives fine‐scale coexistence in cryptic bat species. *Ecology and evolution*.

ROFF, D. A. 1990. The evolution of flightlessness in insects. *Ecological Monographs,* 60**,** 389-421.

ROFF, D. A. 1994. The evolution of flightlessness: is history important? *Evolutionary Ecology,* 8**,** 639-657.

SCUDDER, G. G. E. & CANNINGS, R. A. 2006. Diptera Families of British Columbia.

SITES, R. W., CHAMBERS, W. S. & NICHOL, B. J. 1992. Diel Periodicity of Thrips (Thysanoptera: Thripidae) Dispersion and the Occurrence of Frankliniella williamsi on Onions. *Journal of Economic Entomology,* 85**,** 100-105.

THARANDT, J. L. 2010. Beetles in light trap by-catches (Coleoptera). *Entomologische Nachrichten und Berichte,* 54**,** 1-20.

THOMAS, M. C., SKELLEY, P. E. & FRANK, J. H. 2002. *American Beetles, Volume II: Polyphaga: Scarabaeoidea through Curculionoidea*, CRC Press.

TSUKIJI, T. 2020. Mushi Navi. Tsukiji Takuro.

WEINTRAUB, P. G. & HOROWITZ, A. R. 1996. Spatial and Diel Activity of the Pea Leafminer (Diptera: Agromyzidae) in Potatoes, Solanum tuberosum. *Environmental Entomology,* 25**,** 722-726.

YEE, D. A. & KEHL, S. 2015. Chapter 39 - Order Coleoptera. *In:* THORP, J. H. & ROGERS, D. C. (eds.) *Thorp and Covich's Freshwater Invertebrates (Fourth Edition).* Boston: Academic Press.

YOSHIMURA, M. 2014. Diel response of EPT families to light traps in broad-leaved and planted coniferous forest basins, Japan. *Biological Rhythm Research,* 45**,** 143-156.

***A5*** *Summary of phenological data on prey items from the order Lepidoptera that were identified to species level and were consumed more than once by either of the investigated bat species. The majority of Lepidoptera appear to have been captured as adults and not as caterpillars.*

| **Species** | **Japanese name** | **Adult** | **Distribution in Japan** | **Source*** | **Remark** | **Wingspan (Male)** | **Wingspan (Female)** | **Last instar larva size** | **Occurred in *M. ussuriensis* diet in** | **Occurred in *M. ikonnikovi* diet in** | **Captured as** |
| --- | --- | --- | --- | --- | --- | --- | --- | --- | --- | --- | --- |
| *Archips betulana* | コアトキハマキ | Jun-Aug | Hokkaido | 1 | *Archips betulanus* | 18-21 | 24-26 |  | Aug | Jul | adult |
| *Archips crataeganus* | クロカクモンハマキ | Jul-early Sep | Hokkaido, Honshu | 2 | synonym of *Archips endoi* | 22-24 | 26-29 |  | - | Jul, Aug | adult |
| *Burara aquilina* | キバネセセリ | Jul-Aug | Japan | 3 |  | 21-26 | 21-26 | 25 | Jun, Jul | - | caterpillar in June |
| *Caloptilia cf. heringi* | ヘリングハマキホソガ | May?-Oct | Hokkaido, Honshu | 4 |  | 12-13 | 12-13 |  | - | May, Jul | caterpillar in May |
| *Choristoneura diversana* | コスジオビハマキ | Jun-Jul | Hokkaido, Honshu | 5 |  | 16-20 | 20-25 | 18 | Jul | Jul | adult |
| *Cleora insolita* | ルリモンエダシャク | May-Jul | Japan | 6 |  | 29-32 | 29-32 | 35 | Jul, Aug | - | adult |
| *Ectropis obliqua* | ウスジロエダシャク | Apr-Aug | Japan | 7 |  | 22-30 | 22-30 | 25-30 | Jul, Sep | Jul | adult |
| *Eudemis porphyrana* | サクラマルモンヒメハマキ | Jun-Sep | Honshu | 8 |  | <10mm? | <10mm? |  | - | Jul | adult |
| *Euthrix potatoria* | ヨシカレハ | Jul-Aug | Japan | 9 |  | 45-60 | 50-80 |  | Jul | Jul, Aug | adult |
| *Gelechia cuneatella* | ゴマダラハイキバガ | Jul-Oct | Hokkaido, Honshu | 10 |  | 17 | 17 |  | - | Sep | adult |
| *Hypomecis punctinalis* | ウスバミスジエダシャク | May-Aug | Japan | 11 |  | 32-44 | 32-44 | 35-45 | Aug, Sep | - | adult |
| *Lymantria monacha* | ノンネマイマイ | Jul-Aug | Japan | 12 |  | 37-42 | 41-52 | 20 | Jul | Jul, Sep | adult |
| *Phthonosema tendinosaria* | リンゴツノエダシャク | May-Aug | Japan | 13 |  | 45-58 | 45-58 | 55 | Sep | - | adult |
| *Pseudoips prasinana* | アオスジアオリンガ | Jul-Sep | Japan | 14 | *Pseudoips prasinanus* | 34-39 | 34-39 |  | - | Jul | adult |
| *Psoricoptera gibbosella* | ミツコブキバガ | Jun-Aug | Hokkaido, Honshu | 15 |  | 17-20 | 17-20 |  | Sep | Aug, Sep | adult |
| *Ptycholoma lecheanum* | オオギンスジハマキ | May-Jul | Japan | 16 |  | 17-20 | 22-24 | 18 | - | Jul | adult |
| *Rhopobota naevana* | クロネハイイロヒメハマキ | May-Oct | Japan | 17 |  | 11-23 | 11-23 | 9 | - | Aug, Sep | adult |
| *Spodoptera cilium* | クシナシスジキリヨトウ | Jun-Nov | Japan | 18 | invasive species | 22-24 | 22-24 |  | - | Jul | adult |
| *Spodoptera exigua* | シロイチモジヨトウ | May-Oct | Japan | 19 |  | 25 | 25 | 30 | - | Jul, Sep | adult |
| *Tetheella fluctuosa* | ヒトテントガリバ | Jul-Aug | Hokkaido, Honshu, Shikoku | 20 |  | 32-36 | 32-36 |  | - | Jul, Aug | adult |
| *Zeiraphera rufimitrana* | トドマツアミメヒメハマキ | Jul | Hokkaido, Honshu | 21 |  | 13-15 | 13-15 | 12 | - | Jul | adult |
| *Saturnia japonica* | クスサン屋久島以北亜種 | Sep-Oct | Hokkaido, Honshu, Shikoku, Kyushu, Tsushima, Yakushima | 22 | Appears as a genus in the analysis, as species-level identification in BOLD was uncertain | 100-130 | 100-130 | 100 | Sep | Sep | adult |

* Detailed references:

1. http://www.jpmoth.org/~dmoth/51_Tortricidae/5001%20Tortricinae/framepage_tortricinae1.htm
2. http://www.jpmoth.org/~dmoth/51_Tortricidae/5001%20Tortricinae/framepage_tortricinae1.htm
3. https://www.hro.or.jp/list/forest/research/fri/kanko/fukyu/jumoku/konchu/data/cho-ga/cho/kibanese/kaisetu.pdf
4. http://www.jpmoth.org/Gracillariidae/Gracillariinae/Caloptilia_heringi.html
5. http://www.jpmoth.org/~dmoth/51_Tortricidae/5001%20Tortricinae/framepage_tortricinae2.htm
6. http://www.jpmoth.org/~dmoth/69_Geometridae/72.2_Ennominae/framepage_ennominae3.html
7. http://www.jpmoth.org/Geometridae/Ennominae/Ectropis_obliqua.html
8. http://www.jpmoth.org/Tortricidae/Olethreutinae/Eudemis_porphyrana.html
9. http://www.jpmoth.org/Lasiocampidae/Lasiocampinae/Euthrix_potatoria_bergmani.html
10. http://www.jpmoth.org/Gelechiidae/Gelechiinae/Gelechia_cuneatella.html
11. http://www.jpmoth.org/Geometridae/Ennominae/Hypomecis_punctinalis_conferenda.html
12. http://www.jpmoth.org/Lymantriidae/Lymantria_monacha.html, https://www.hro.or.jp/list/forest/research/fri/kanko/fukyu/jumoku/konchu/data/cho-ga/dokuga/nonnema/kaisetu.pdf
13. http://www.jpmoth.org/Geometridae/Ennominae/Phthonosema_tendinosarium.html
14. http://www.jpmoth.org/Nolidae/Chloephorinae/Pseudoips_prasinanus.html
15. http://www.jpmoth.org/Gelechiidae/Gelechiinae/Psoricoptera_gibbosella.html
16. http://www.jpmoth.org/Tortricidae/Tortricinae/Ptycholoma_lecheanum_circumclusanum.html
17. http://www.jpmoth.org/Tortricidae/Olethreutinae/Rhopobota_naevana.html
18. http://www.jpmoth.org/Noctuidae/Hadeninae/Spodoptera_cilium.html invasive species
19. http://www.jpmoth.org/Noctuidae/Hadeninae/Spodoptera_exigua.html
20. http://www.jpmoth.org/Drepanidae/Thyatirinae/Tetheella_fluctuosa_isshikii.html
21. http://www.jpmoth.org/Tortricidae/Olethreutinae/Zeiraphera_rufimitrana_truncata.html
22. http://www.jpmoth.org/Saturniidae/Saturniinae/Saturnia_japonica_japonica.html

*A6 PCR replicates*

Each PCR for each sample was carried out in two separate replicates (replicate 1 and 2). We plotted the replicates as a function of each other and fitted a linear curve to the scatter plot (Figure A6.1). We further tested the correlation between replicates by Pearson linear correlation test (COI: *r*=0.849, P<0.0001; 16S: *r* = 0.995, P<0.0001).

**Figure A6.1** Read count for each sample PCR replicates for A) COI and B) 16S data.

*A7 Accumulation curves*

**Figure A7.1** Accumulation curves for (**A**) *Murina ussuriensis* (n=53) and (**B**) *Myotis ikonnikovi* (n=45) depict the degree to which our present sampling effort covers the richness of the prey at four taxonomic levels (species, genus, family and order). Estimates were computed based on 100 randomisations, and the number of prey species, genera, families and orders were extrapolated to a total of 200 samples.

*A8 List of families depicted in Figure 6*

**Araneae:** **1**Lycosidae, **2**Salticidae;

**Coleoptera:** **3**Cantharidae, **4**Carabidae, **5**Cerambycidae, **6**Chrysomelidae, **7**Melandryidae, **8**Nitidulidae, **9**Scarabaeidae, **10**Tenebrionidae;

**Dermaptera:** **11**Forficulidae;

**Diptera:** **12**Anthomyiidae, **13**Cecidomyiidae, **14**Chironomidae, **15**Chloropidae, **16**Culicidae, **17**Drosophilidae, **18**Empidoidea (Superfamily), **19**Limoniidae, **20**Muscidae, **21**Mycetophilidae, **22**Pediciidae, **23**Periscelididae, **24**Phoridae, **25**Psychodidae, **26**Sarcophagidae, **27**Sciaridae, **28**Simuliidae, **29**Sphaeroceridae, **30**Syrphidae, **31**Tachinidae, **32**Tipulidae, **33**Unknown;

**Ephemeroptera:** **34**Heptageniidae;

**Hemiptera:** **35**Miridae, **36**Psyllidae;

**Hymenoptera:** **37**Braconidae, **38**Cimbicidae, **39**Pamphiliidae, **40**Tenthredinidae, **41**Unknown;

**Lepidoptera:** **42**Autostichidae, **43**Blastobasidae, **44**Coleophoridae, **45**Crambidae, **46**Depressariidae, **47**Drepanidae, **48**Elachistidae, **49**Erebidae, **50**Gelechiidae, **51**Geometridae, **52**Gracillariidae, **53**Hesperiidae, **54**Lasiocampidae, **55**Limacodidae, **56**Noctuidae, **57**Nolidae, **58**Notodontidae, **59**Nymphalidae, **60**Oecophoridae, **61**Saturniidae, **62**Sphingidae, **63**Tortricidae, **64**Uraniidae, **65**Unknown;

**Mecoptera:** **66**Panorpidae;

**Neuroptera:** **67**Chrysopidae, **68**Hemerobiidae, **69**Osmylidae;

**Odonata:** **70**Libellulidae;

**Orthoptera:** **71**Rhaphidophoridae;

**Plecoptera:** **72**Nemouridae, **73**Perlidae;

**Psocodea:** **74**Psocidae, **75**Stenopsocidae;

**Thysanoptera:** **76**Thripidae;

**Trichoptera:** **77**Limnephilidae, **78**Thremmatidae
